# Synthesis of thiopyran derivatives *via* [4 + 2] cycloaddition reactions

**DOI:** 10.1039/d5ra01222h

**Published:** 2025-04-09

**Authors:** Maryam Mousavi-Ebadia, Javad Safaei-Ghomi, Masoumeh Jadidi Nejad

**Affiliations:** a Department of Organic Chemistry, Faculty of Chemistry, University of Kashan Kashan 51167 I. R. Iran safaei@kashanu.ac.ir; b Department of Chemistry, Isfahan University of Technology Isfahan 84156-83111 Iran

## Abstract

In this review, we provide a comprehensive overview of the synthesis of thiopyran family compounds *via* cycloaddition reactions, with examples spanning from the year 2000 to the present. We have categorized the [4 + 2] cycloaddition processes using several criteria, particularly distinguishing between intermolecular and intramolecular types based on the Diels–Alder partners. Additionally, from a mechanism standpoint, we differentiate between concerted and stepwise [4 + 2] processes, offering an analysis of these mechanisms based on the current literature.

## Abstract 2

In this review, we provide a comprehensive overview of the synthesis of thiopyran family compounds *via* cycloaddition reactions, with examples spanning from the year 2000 to the present.

## Graphical abstract

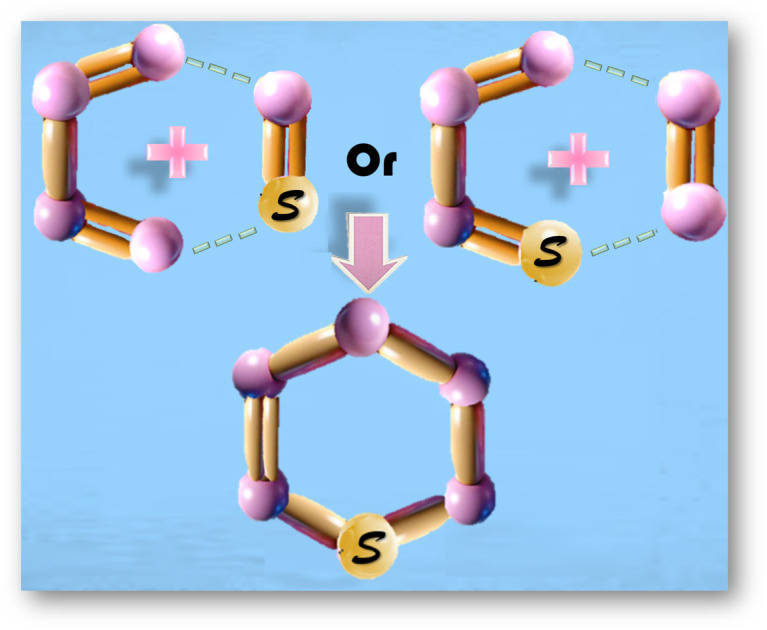

## 1 Introduction

**Fig. 1:**
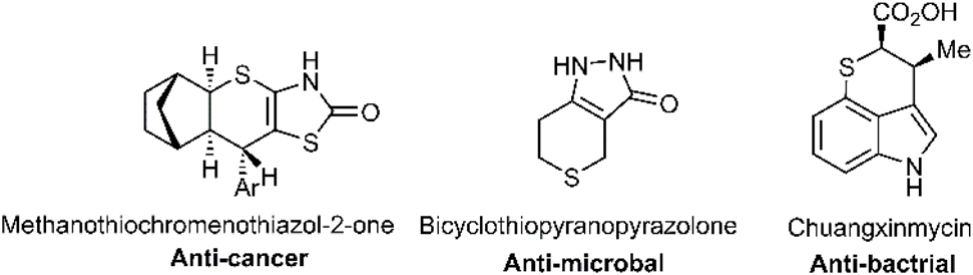
Pharmaceutical structures based on thiopyran.

For decades, sulfur-containing heterocycles have been a central focus of research in various branches of chemistry due to their unique properties, which facilitate the conversion of functional groups for the synthesis of bioactive compounds.^1–8^ These heterocycles are integral to everyday life, as many are key components of natural products, such as penicillin, one of the greatest achievements of the twentieth century. Over twenty sulphur derivatives of amino acids have been identified, and examples can be found in fossil fuels and petroleum products. Additionally, sulphur-containing heterocycles have broad applications in the synthesis of disinfectants, antibiotics, antioxidants, dyes, pigments, and especially in the pharmaceutical industry.^9,10^ Thiocarbonyl compounds act as versatile intermediates in the synthesis of polycyclic compounds. The increasing interest in thiocarbonyl chemistry highlights their potential to generate a wide range of products. The electronegativity of sulphur is only slightly higher than that of carbon, resulting in a C=S bond polarity opposite to that of the carbonyl bond, with a lower overall polarity. As a result, the positive charge on the carbon atom is reduced, making the C=S bond less stable than the carbonyl bond and favouring sp^3^ hybridization.^11,12^ Thiocarbonyls can function both as dienes and dienophiles, allowing for the formation of [4 + 2] cycloadducts depending on their molecular positioning.^13,14^ Due to their elevated HOMO and reduced LUMO energy levels compared to carbonyls, these compounds are often activated as dienophiles through electron acceptor binding to the carbon atom, enhancing orbital overlap in the Diels–Alder reaction.^15,16^ The D–A reaction is considered one of the most transformative processes in organic chemistry, known for its efficiency in forming two carbon–carbon bonds and up to four new stereocenters, which underscores its versatility in natural product and pharmaceutical synthesis.^17–19^ In classical literature, D–A reactions are generally described as concerted. Lewis's acids, such as BF_3_·OEt_2_, are commonly used as catalysts to activate cycloaddition partners by narrowing the energy gap between frontier orbitals.^20,21^ Based on DFT and the ELF calculations, Domingo has proposed a detailed mechanism for [4 + 2] cycloaddition reactions, distinguishing between concerted and stepwise types depending on intermolecular interactions. Both polar and non-polar stepwise reactions can occur; however, a zwitterionic intermediate is favoured in polar interactions, while a diradical intermediate predominates in non-polar conditions.^22–29^ Understanding reaction mechanisms offers valuable insights into reaction dynamics and molecular stability, which are crucial for advancing synthetic methodologies and understanding fundamental chemical processes. Pericyclic reactions are celebrated for their ability to synthesize complex compounds under mild conditions with high atom economy, making them essential tools in synthetic chemistry.^30–34^ Despite more than 80 years since the discovery of the Diels–Alder reaction as one of the most renowned pericyclic reactions, it remains a key concept in organic chemistry. The Hetero-Diels–Alder reaction offers a straightforward approach to synthesizing important six-membered heterocycles with high stereoselectivity.^35–41^ While oxo D–A and aza D–A reactions have received significant attention, the thio D–A reaction is fundamental in forming various medicinal polycyclic compounds based on the thiopyran scaffold.^42^ The therapeutic potential of thiopyrans has recently gained recognition for their importance in medicinal chemistry.^43^ For instance, chlorprothixene, a member of the thioxanthene class, serves as both an antidepressant and an antipsychotic drug. Additionally, tricyclic thiopyran-2-ones have proven effective in combating viral infections, while fragrant compounds containing thiopyran-carbaldehyde further demonstrate the practical significance of these compounds in various real-world applications.^44^ Woodward's groundbreaking work in 1982 demonstrated the utility of thiopyrans in simplifying the preparation of polypropionates during the total synthesis of rythromycin A.^45,46^ Thiopyrans are crucial building blocks in the structures of various bioactive agents, including antibacterial, anti-inflammatory, anticancer, and anti-atherosclerotic structures.^47,48^ Several biologically active derivatives of these compounds are shown in Fig. 1.^49–52^

Over time, there has been growing interest in developing innovative strategies to transform organosulfur precursors into thiopyran motifs for pharmaceutical applications. Numerous studies on thiopyrans have been conducted to date. Existing reviews typically explore topics such as Diels–Alder reactions, the clinical applications of thiopyrans, and the synthesis and utility of thiopyran-linked polycyclic systems.^53–58^ These comprehensive reviews offer valuable insights into the advancements in thiopyran research, highlighting their chemical synthesis and diverse applications. By shedding light on these developments, they inspire researchers to further explore new frontiers in the field. However, we have noted that these advancements have been somewhat fragmented in the synthesis literature. To address this gap, we have organized the synthesis methods of thiopyran structures *via* cycloaddition into a coherent framework, reviewing them from multiple perspectives. This review focuses on the synthesis of six-membered sulfur heterocycles through the [4 + 2] cycloaddition strategy, utilizing thiocarbonyl-functionalized precursors, which can act as either dienophiles or diene components. The review covers a broad spectrum of organosulfur compounds, ranging from common to rare, both cyclic and linear. We aim to provide a comprehensive overview of unique studies published from 2000 to 2025 in this area.

### 1.1 Intermolecular cycloaddition reactions

Intermolecular cycloaddition reactions involve the formation of rings from reactants derived from separate molecules. The conversion of two distinct entities into a cycloadduct typically occurs in opposition to entropy, resulting in a reaction rate and efficiency that are generally lower than those observed in intramolecular processes.^59^

#### 1.1.1 Concerted reactions

**Fig. 2:**
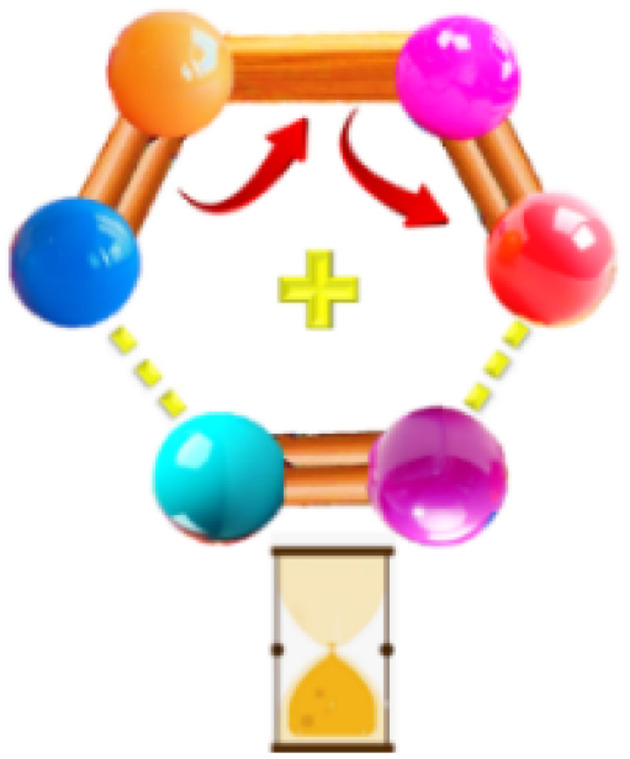
General schematic of concerted [4 + 2] cycloaddition reactions.

Concerted reactions are characterized by the simultaneous rearrangement of π bonds and the formation of new σ bonds in a single step (see Fig. 2). These processes are driven by the movement of delocalized electrons, as seen in pericyclic reactions like the D–A reaction, 1,3-dipolar cycloadditions, and sigmatropic rearrangements.^60^ A key feature of concerted reactions is the absence of separable intermediates; instead, they proceed through a transient transition state (TS). Symmetrical molecular structures often favor a concerted pathway.^61,62^ If the transition state is stabilized through effective orbital overlap, the reaction can proceed in a concerted manner.^63^ By analyzing the stereochemistry of the TS and employing DFT calculations, researchers can predict the structure of the final product, along with its stereochemical and regioselective outcomes.

#### 1.1.2 Thione-containing dienophiles

**Fig. 3:**
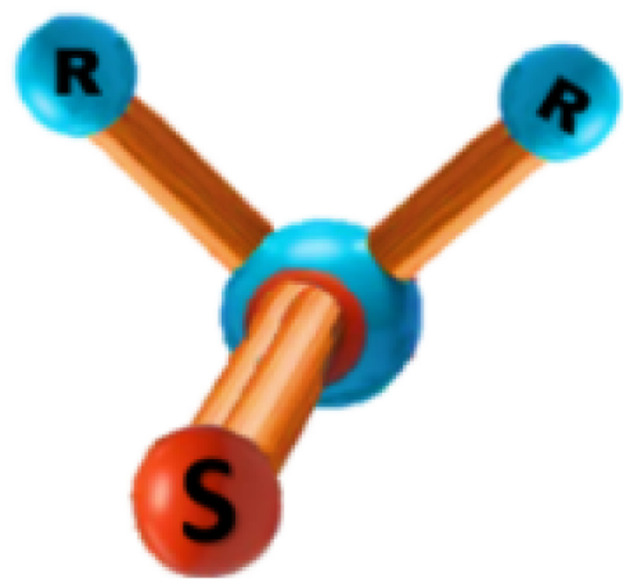
Schematic of thiodieneophiles.

Within the framework of the D–A reaction, a dienophile serves as the acceptor component. These dienophiles typically feature double bonds that are activated by substituents, allowing them to contribute electrons to the [4 + 2] cycloaddition process. Notably, sulfur-containing dienophiles are highly reactive, readily donating their electrons in these transformations (Fig. 3).^64^

##### 1.1.2.1 Dithioesters

Dithioesters are unique esters in which sulfur atoms replace the oxygen atoms typically found in conventional structures. Among thiocarbonyl derivatives, dithioesters exhibit remarkable nucleophilic strength, a characteristic attributed to the high polarizability of the sulfur groups. Numerous studies have documented the involvement of dithioesters in nucleophilic substitution, cycloaddition and radical reactions and polymerization processes.^65–68^

**Scheme 1:**
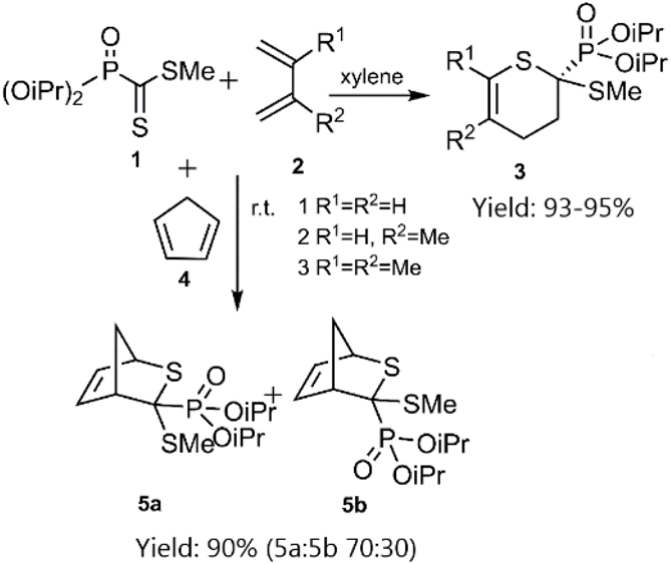
Thio D–A cycloadditions of phosphanecarbodithioate 1 with usual dienes.

**Scheme 2:**
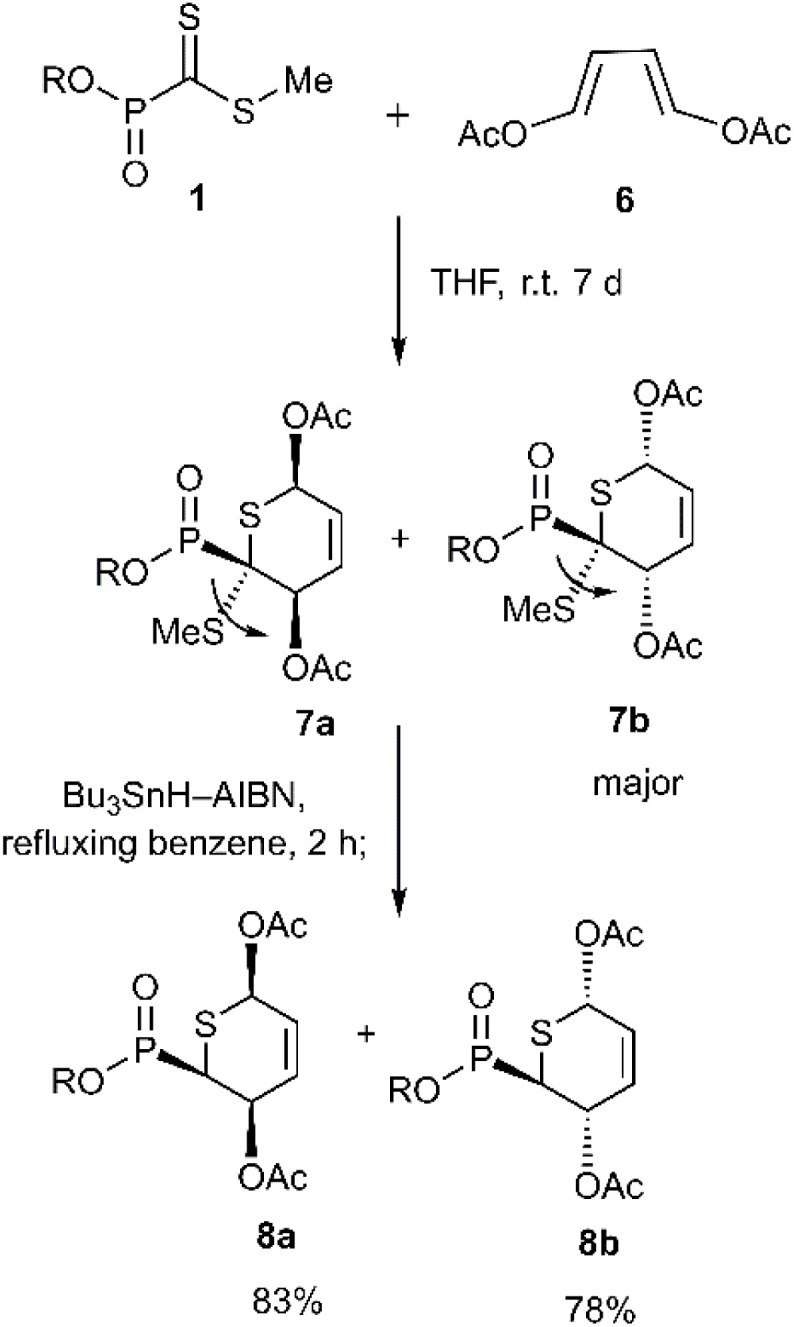
Synthesis of a phosphonothiashikimic acid derivatives 8a-b.

**Scheme 3:**
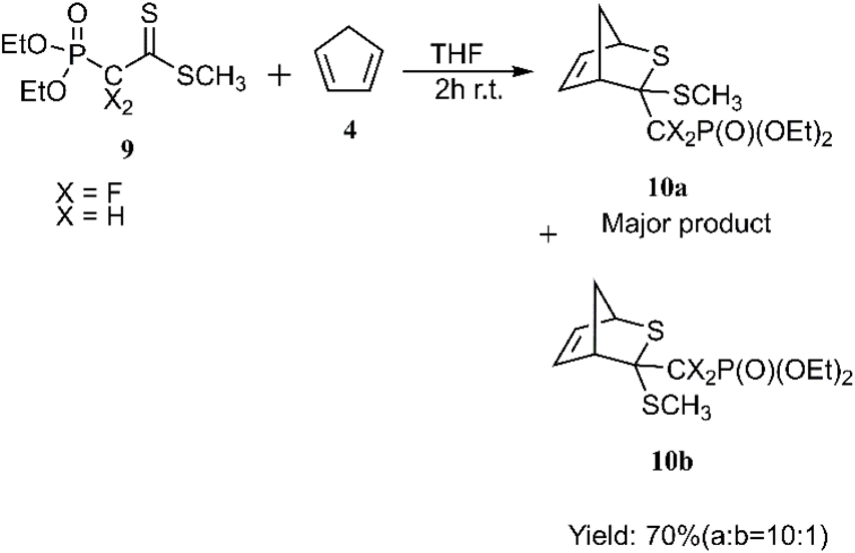
Cycloaddition reaction of phosphonodifluorodithioacetate 9.

In a notable investigation, Masson *et al.* demonstrated the application of the D–A reaction for synthesizing phosphonic-substituted dihydrothiopyran derivatives (Schemes 1–3). In this account, phosphonic-substituted dithioformates 1, 9 were reacted with usual dienes such as functionalized butadiene 2, 6 and cyclopentadiene 4, yielding a with high yield. It is noteworthy that the product featuring an *exo*-phosphonate group 5a was the preferred cycloadduct (Scheme 1), and the use of Bu_3_SnH as a Lewis acid facilitated the selective desulfanylation of the thio D–A products without disrupting the thiopyran ring (Scheme 2).^69,70^

**Scheme 4:**
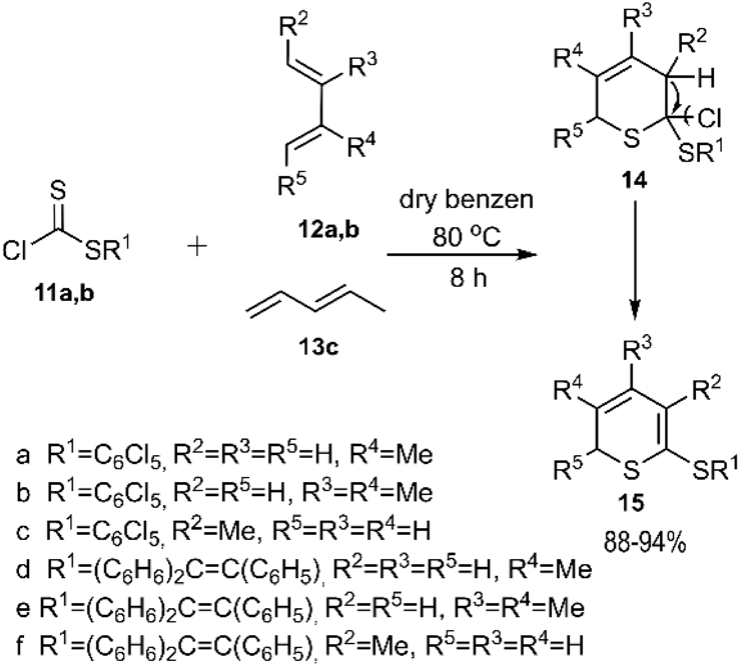
D–A reaction of alkylcarbonochloridodithioate 11a-b with acyclic dienes 12a, 12b and 13.

In that same year, El-Sayed and colleagues unveiled an intriguing pathway for thio D–A reactions aimed at the regioselective synthesis of 2*H*-thiopyran derivatives. The authors noted that the observed regioselectivity of the products was unexpected, yet it could be rationalized by steric effects. In their study, thioesters 11a-b reacted with dienes 12a-b and 13 to yield compound 15, which was subsequently transformed into the final product after spontaneous elimination and the removal of HCl. Dithioester 14b followed a similar pathway under identical conditions, leading to the formation of heterocycles 14e-f and 15e-f (Scheme 4).^71^

**Scheme 5:**
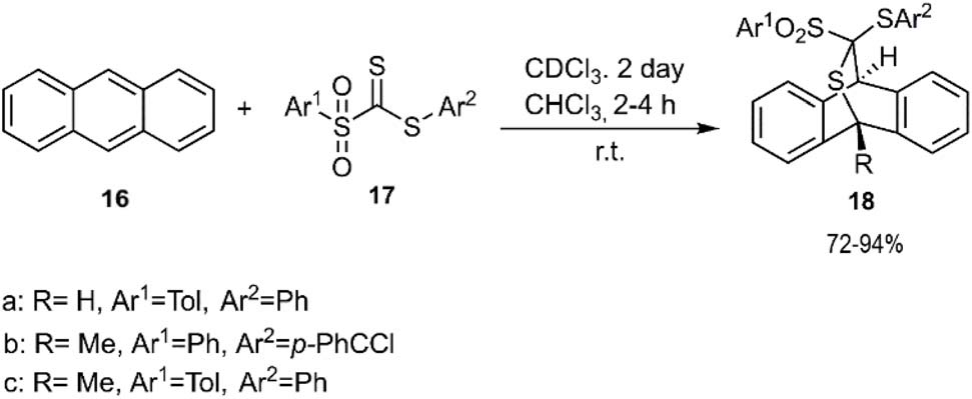
D–A reaction of dienophile 17 with anthracene 16.

Shortly after this groundbreaking work, El-Sayed *et al.* reported a similar synthetic route for the preparation of (epithiomethano)anthracene sulfanediones 18 (Scheme 5). In their study, two anthracene derivatives (R^3^= H, methyl) were employed as 1,3-diene species, reacting with various *C*-sulfonyldithioformate derivatives 17 to achieve excellent stereoselectivity. This reaction subsequently led to the elimination of arenesulfinic acid from the D–A cycloadducts.^72^

**Scheme 6:**
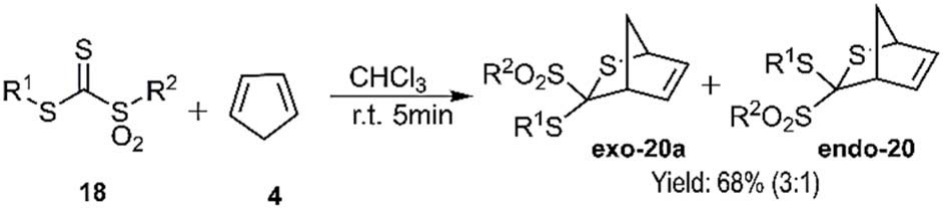
D–A reaction of compound 19 with 1, 3-pentadiene 4.

El-Sayed and colleagues proposed a hetero D–A reaction that facilitated the construction of dihydrothiopyran derivatives. This innovative approach utilized *C*-sulfonyldithioformates 19 as super hetero dienophiles in combination with 1,3-dienes 4, 22, 24, and 13. Notably, when employing 1,3-pentadiene 21, the resulting products exhibited an *endo* preference at room temperature (Scheme 6).

**Scheme 7:**
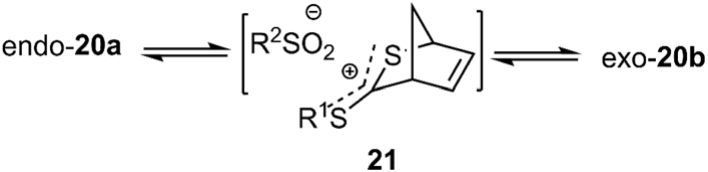
Intermediate 21 in conversion *endo*-20a to *exo*-20b.

**Scheme 8:**
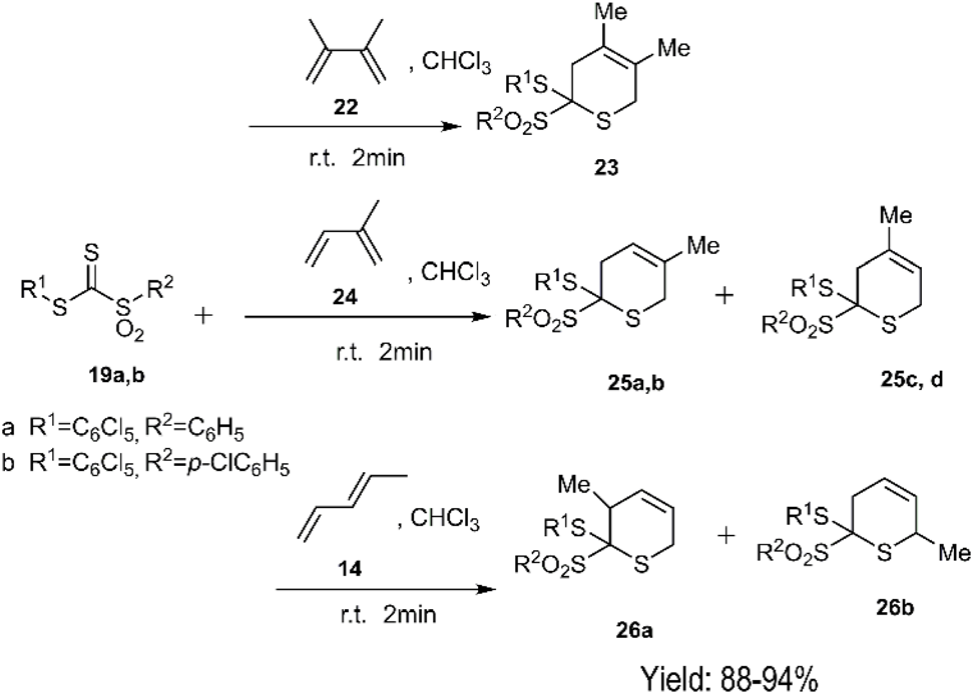
D–A reaction of *C*-sulfonyldithioformates 19 with dienes 22, 24, 13.

It is worth mentioning that upon chromatography on silica gel using methylene chloride as the eluent, *endo*-20a was quantitatively converted to *exo*-20b. This transformation highlights the stability of *exo*-22b due to its lower steric hindrance, facilitated through ion pair 21 (Scheme 7). The products 25a, 25b, and 26a demonstrated favourable regioselectivity (Scheme 8).^73^

**Scheme 9:**
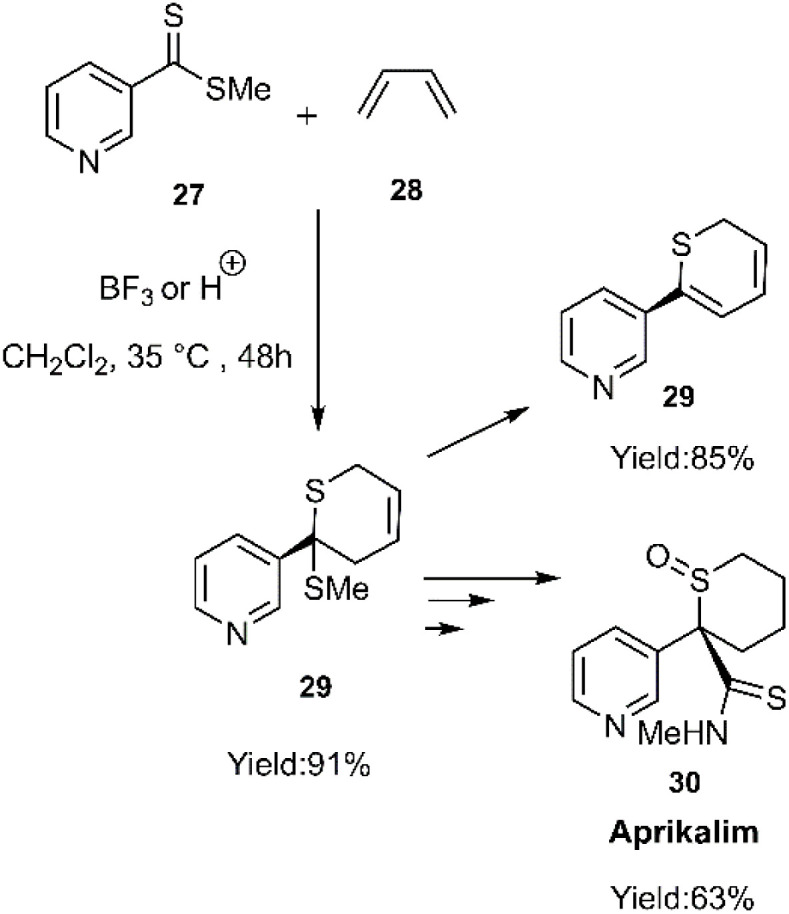
HD–A reaction of butadiene with pyridinedithioester 27.

**Scheme 10:**
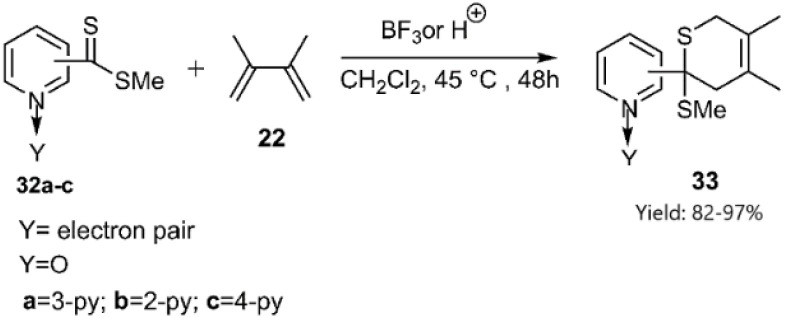
HD–A reaction of butadiene derivative 22 with dithioesters 32a–c.

Gulea and her team explored the impact of pyridinedithioesters 27 on enhancing the reaction progress with common dienes, ultimately yielding new derivatives of the biologically relevant aprikalim 31 scaffold (Scheme 9). To further increase the reactivity of the hetero dienophile, *N*-oxide pyridine thioester 32 was employed in place of 27, resulting in satisfactory outcomes (Scheme 10).^74^

**Scheme 11:**
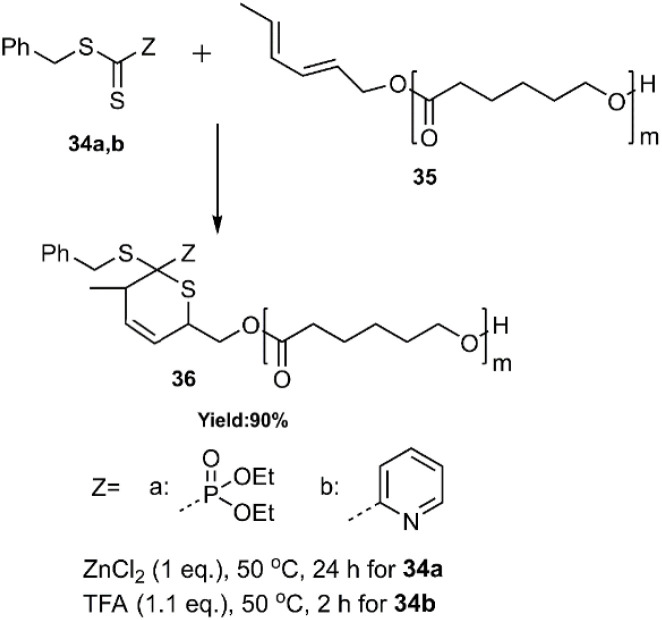
Preparation of dihydro-2*H*-thiopyran functionalized poly (ethylene glycol) 36*via* HD–A reaction.

In 2008, Sinnwell and colleagues introduced a novel approach by employing the thio D–A reaction to synthesize dihydro-2*H*-thiopyran functionalized poly ethylene glycol 36. In this innovative reaction, the terminal diene group is situated on the polyethylene glycol, facilitating RAFT polymerization with functionalized polystyrene (see Scheme 11).^75^

**Scheme 12:**
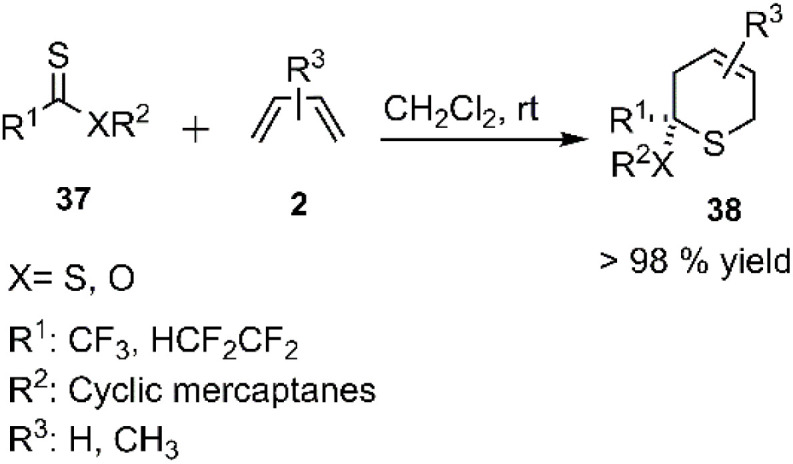
Thio D–A reaction of polyfluoroalkylthionocarboxylates 37.

Timoshenko *et al.* reported the thio D–A reaction involving chiral polyfluoroalkylthionocarboxylates 37 and butadiene derivatives 2. The resulting compounds exhibited moderate diastereoselectivity in the Diels–Alder reactions of thioesters (refer to Scheme 12).^76^

**Scheme 13:**
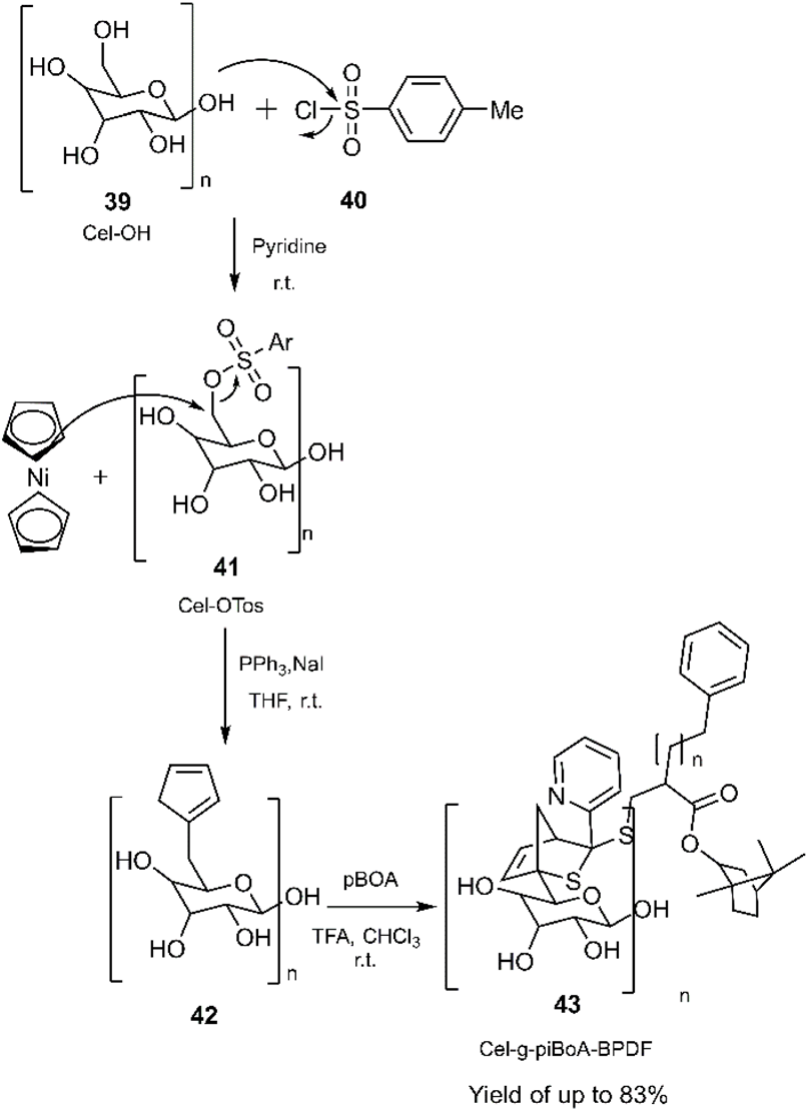
A sample of HD–A reaction in order to modification of macromolecules.

Goldmann and collaborators successfully combined RAFT polymerization with a hetero D–A reaction involving a 2-pyridinedithioester derivative 42 and cyclopentadiene grafted onto cellulose 43 (see Scheme 13).^77^

**Scheme 14:**
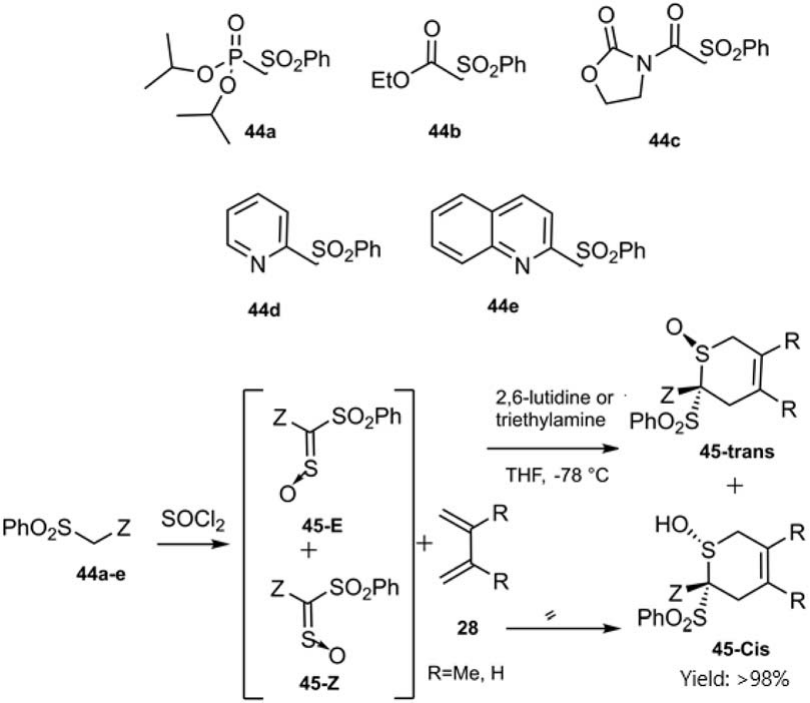
HD–A reaction of butadiene derivatives 28 with dithioesters 44a–e.

**Scheme 15:**
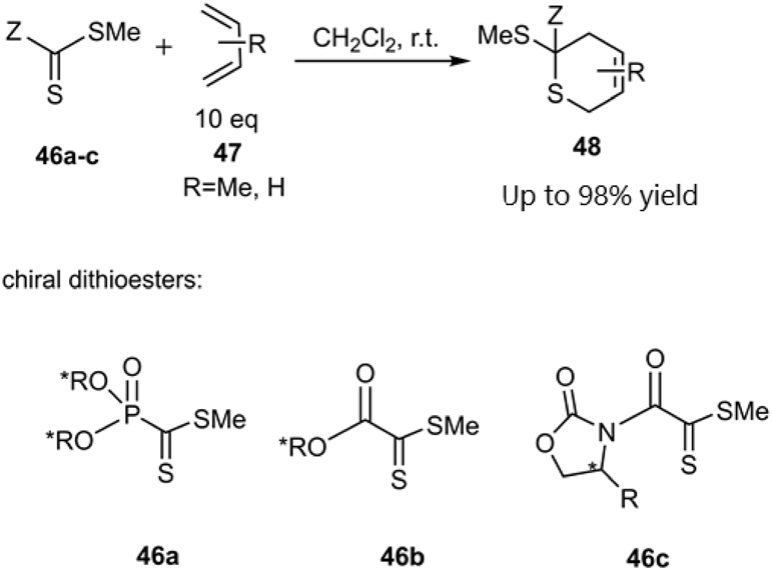
HD–A reaction of butadiene derivatives with dithioesters 47a–c.

**Scheme 16:**
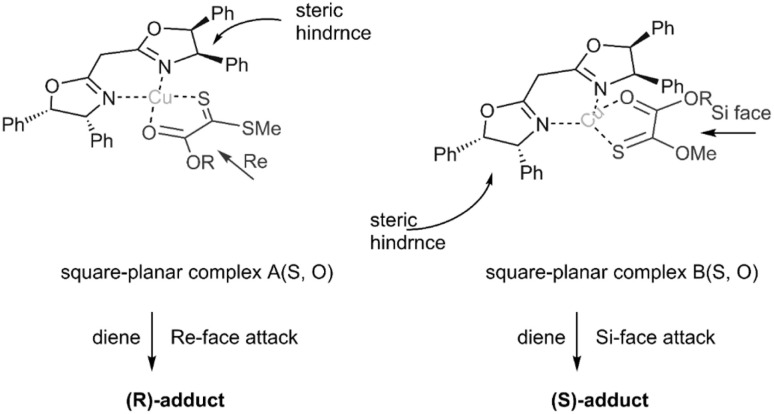
Stereo chemical outcome in thio D–A reaction of dithiooxalates 47.

Gulea's research team unveiled novel dithioester substrates as dienophiles, derived from the addition–elimination of a sulfonyl molecule (Scheme 14). The observed *trans*-selectivity at room temperature can be attributed to the significant electron-withdrawing effect of the pyridyl or quinolyl group on the sulfonyl moiety, as other thioester substrates yielded a mixture of both isomers. Expanding upon their previous work, this research team explored the reaction of three distinct chiral dithioesters with simple dienes to synthesize valuable compounds bearing novel chiral stereocenters (Scheme 15). The employment of an asymmetric Cu(ii)-bis(oxazoline) catalyst facilitated chelate formation during the reaction, leveraging its impressive stereo control to achieve high diastereomeric excess (90% de). DFT studies indicated that the Si-face approach was preferred due to reduced steric hindrance (Scheme 16).^78,79^

**Scheme 17:**
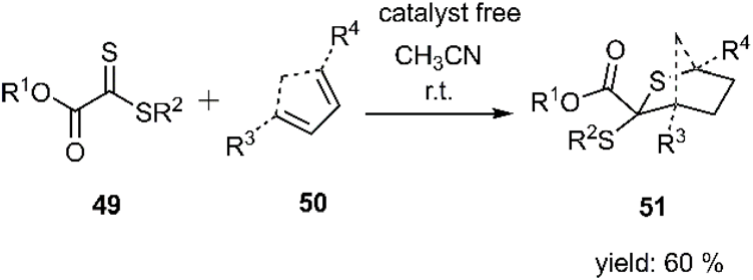
HD–A reaction of thioxoacetate derivative 49 with different dienes.

Lederer and colleagues reported a thermo-sensitive thio D–A reaction, utilizing the synthesis and application of polyfunctional dithiooxalate derivatives 49 as thiophilic dienophiles under ambient conditions. This reaction has been explored with a variety of dienes, encompassing cyclic and acyclic structures, as well as those exhibiting s-*cis* or s-*trans* conformations (Scheme 17).^80^

**Scheme 18:**
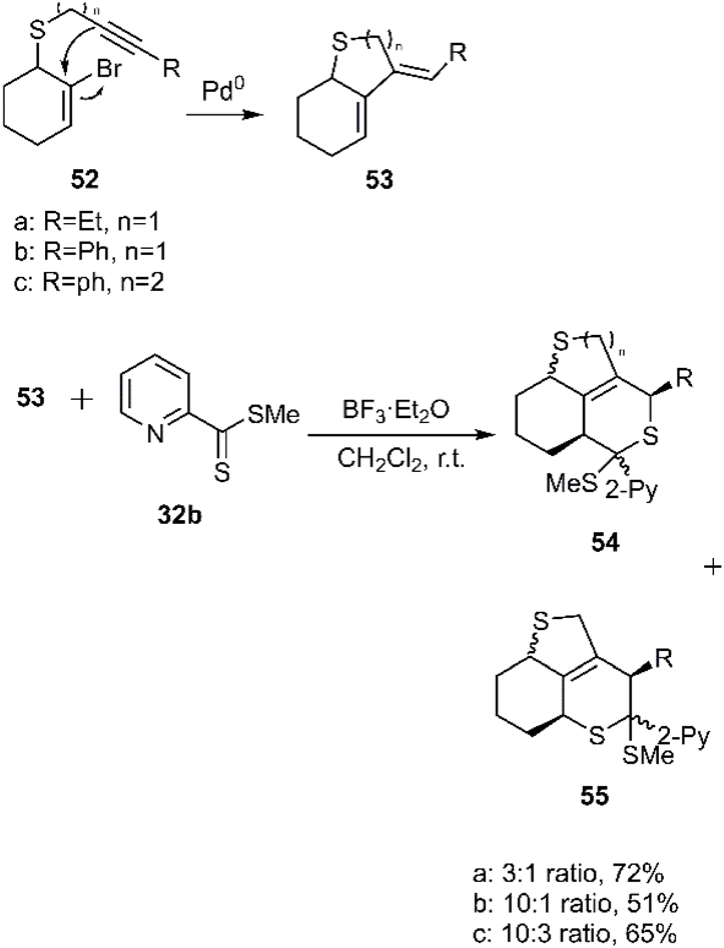
H D–A reaction of methyl pyridine-2-carbodithioate 32b and 2, 3-*exo*-bicyclic dienes 53a–c.

Gulea *et al.* described a distinct synthetic pathway for the preparation of organosulfur compounds. *Exo*-bicyclic 1,3-dienes were accessed *via* a metal-catalyzed intermolecular Sonogashira coupling of 2-bromocyclohex-2-en-1-ylsulfane derivatives 52a–c. Subsequently, the *exo*-bicyclic thio dienes 53a–c were subjected to cycloaddition reactions with various dienophiles to investigate their reactivity The thio D–A reaction of methyl pyridine-2-carbodithioate 32b yielded regioisomeric products 54 and 55 (Scheme 18).^3^

**Scheme 19:**
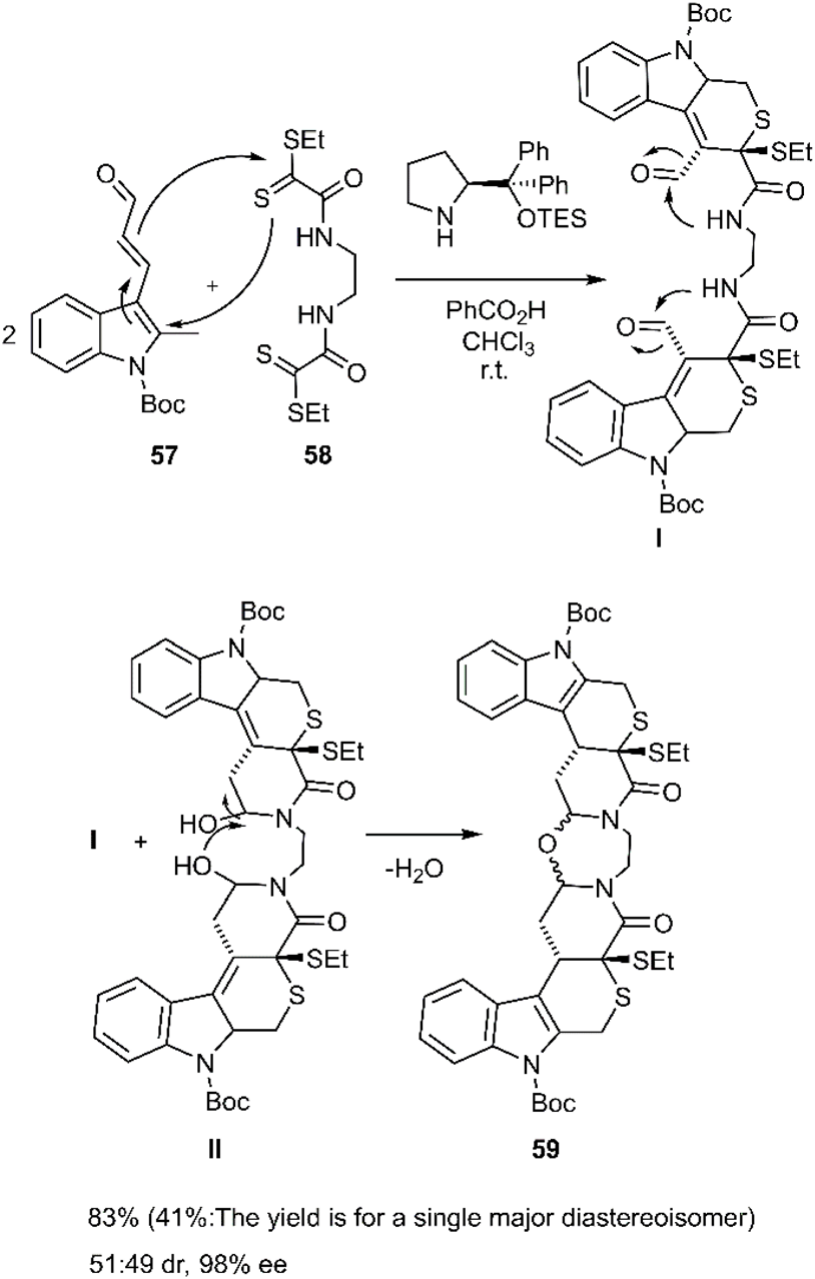
D–A reaction in order to synthesize thio pyranopiperidone 59.

Cruz Cruz *et al.* broadened the scope of polycyclic synthesis by demonstrating a hetero-Diels–Alder/intramolecular cycloaddition cascade catalyzed by trienamines, affording nine-membered ring systems with excellent enantioselectivity. This strategy involved a double [4 + 2] cycloaddition reaction between (*E*)-oxopropenylindole carboxylate derivative 57 and bis-dithioamide 58 (Scheme 19). The group further extended this methodology to the synthesis of diverse polycycles, employing dienals and bis 2-oxoethanedithioates 58 as starting materials.^81^

**Scheme 20:**
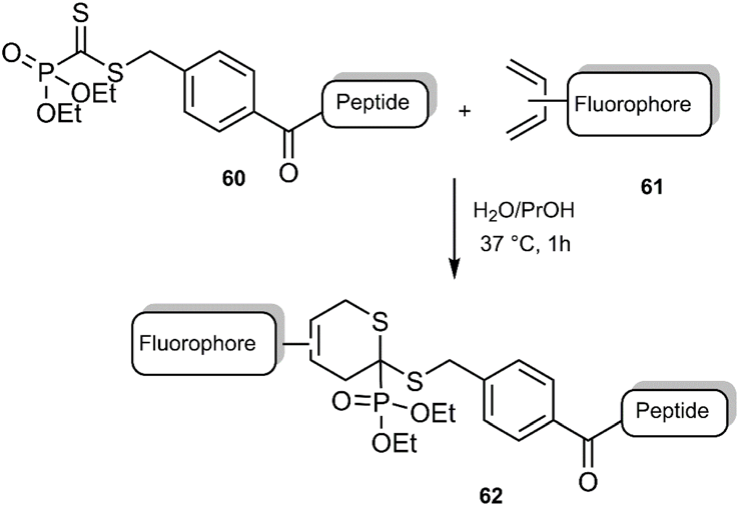
Click thio D–A reaction in order to synthesize the peptides 62.

The Gulea research group has recently demonstrated a novel and practical application of the thio D–A reaction, extending its utility into the realm of biological imaging. This work leverages the D–A reaction for the synthesis of novel fluorescent peptides, enabling the visualization of living cells. The approach involves a straightforward, uncatalyzed click reaction between phosphodiester peptides 60 and fluorophore-decorated dienes 61*via* a thio D–A cycloaddition (Scheme 20).^82^

##### 1.1.2.2 Thioaldehydes

Thioaldehydes, featuring the H–C=S functional group, are sulfur analogs of conventional aldehydes. They serve as valuable intermediates in the construction of complex polycyclic systems, particularly within the context of cycloaddition reactions. However, thioaldehydes are inherently unstable, readily undergoing dimerization or polymerization, and often yielding a mixture of products in their reactions. Isolation of thioaldehydes is challenging, leading to their prevalent use as *in situ* generated and consumed reagents. Nevertheless, research has demonstrated that electron-donating substituents can enhance the stability of thioaldehydes, thereby facilitating their isolation.^83–86^

**Scheme 21:**
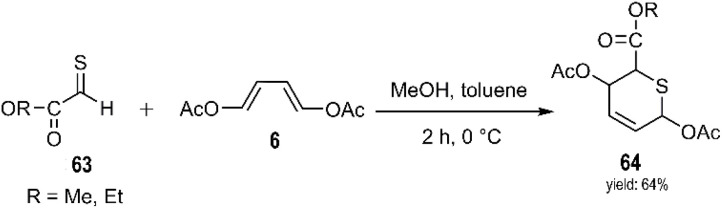
Thio D–A reaction of thioaldehydes derivatives 63 and buta-1, 3-dienederivative 6.

Sakakibara and Watanabe reported an efficient and straightforward synthesis of dihydro-2*H*-thiopyran-2,5-diyl diacetate 64 through a thio-D–A reaction between thioaldehydes 63 and 1,3-butadiene derivatives (Scheme 21).^87^

**Scheme 22:**
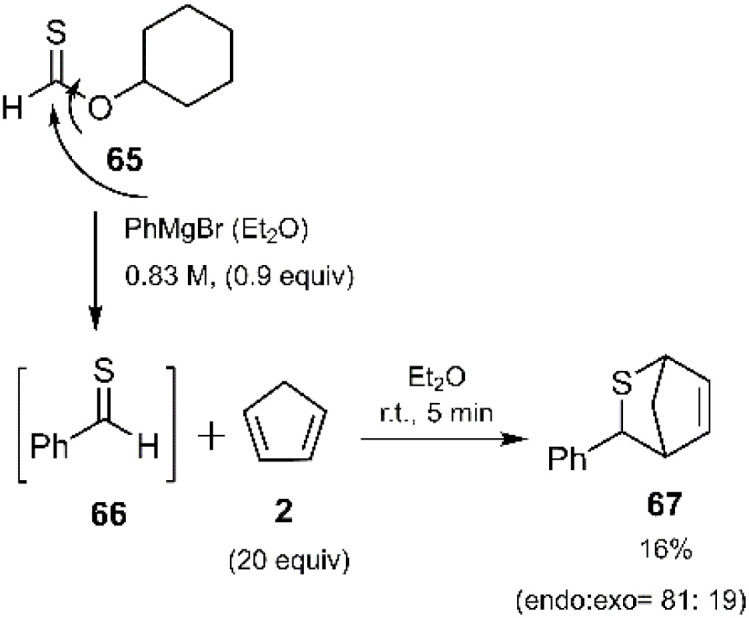
D–A reaction of *in situ* generated thioaldehyde 66 with cyclopentadiene.

*In situ* generated thiobenzaldehyde 66, prepared *via* reaction with phenyl Grignard reagent, underwent a highly selective *endo*-thio D–A cycloaddition with cyclopentadiene, affording the corresponding cycloadducts as major products (Scheme 22). This rapid and innovative reaction, reported by Murai and coworkers, enabled the efficient synthesis of valuable heterocyclic compounds.^88^

**Scheme 23:**
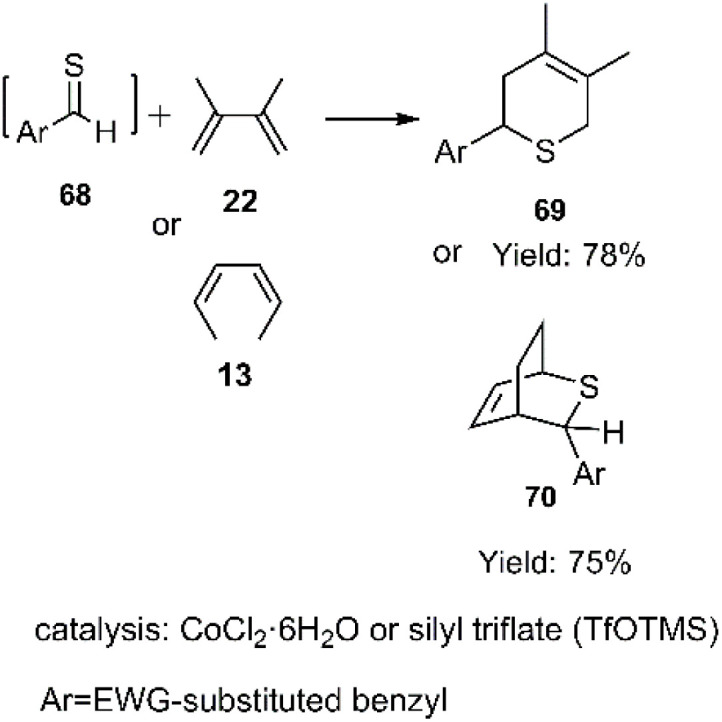
D–A reaction of *in situ* generated thiodienophiles 69 with dienes 22 and 13.

Tanini and coworkers reported the thionation of aldehydes through mild conditions with different catalyst systems. Thio D–A cycloadducts were produced *in situ* by trapping the corresponding thioaldehydes by diene using cobalt salt in ionic liquids or silylated catalysts (Scheme 23). Finally, *endo*-isomer dihydrothiopyran derivatives 69 and 70 were obtained with good yields.^89^

**Scheme 24:**
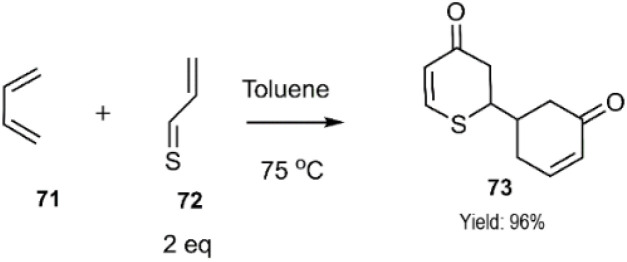
Double cycloaddition of thioacrolein 72 with Danishfsky's diene.

Furthermore, the Nakamura research group explored the *in situ* generation of thioacrolein 72 from alysin, leveraging this reactive intermediate in a thio D–A reaction to synthesize antitumor dihydrothiopyran derivatives 73. Notably, this highly reactive intermediate, when reacted with a pair of Danishefsky's diene molecules 71, exhibited dual nucleophilic character, participating in a two-fold D–A cycloaddition (Scheme 24).^90^

##### 1.1.2.3 Thioamides

Thioamides, also known as thioureylenes, are characterized by the presence of the N–C=S functional group. These compounds have found applications in both drug development and biosynthesis. The enhanced resonance stabilization of thioamides compared to their amide counterparts results in a higher rotational barrier. This unique property has enabled synthetic chemists to utilize thioamides as versatile building blocks in the construction of diverse heterocyclic frameworks.^91–94^

**Scheme 25:**
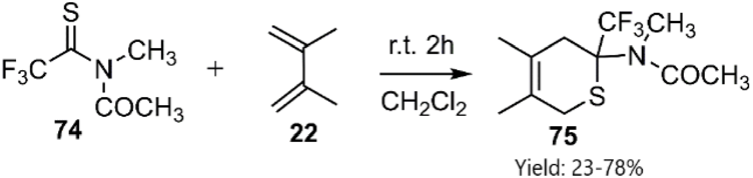
D–A reaction of thioamides 78 and 79 and diene 22.

In a pioneering study conducted Bouillon *et al.* reported the first successful synthesis of novel dihydrothiopyran derivatives *via* a highly efficient thio-D–A reaction employing a polyfluorothioamide derivative 74. While conventional approaches, such as increasing pressure or temperature, and the use of basic catalysts, were explored for analogous thioamides, these methods proved ineffective in achieving comparable outcomes (Scheme 25).^95^

##### 1.1.2.4 Thioketones

Thioketones constitute a prominent class of organosulfur compounds. Characteristically exhibiting orange or brown coloration, these highly reactive species serve as valuable dienophiles in cycloaddition reactions. Notable examples of thioketones include thiobenzophenone, thioxanthone, and thioisatin.^96,97^

**Scheme 26:**
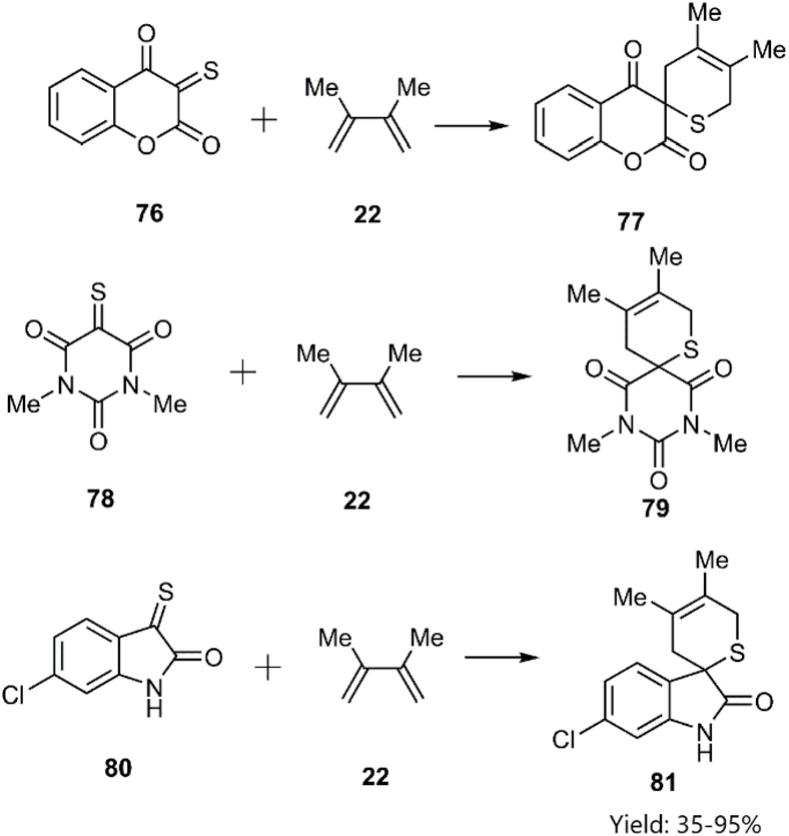
D–A reaction of thiodienophiles 76, 78 and 80 with 2, 3-methyle butadiene 22.

Shermolovich *et al.* reported the synthesis of a novel series of heterocyclic compounds featuring spirocyclic thione substituents through a thio D–A cycloaddition strategy. Their approach involved the initial selection of heterocycles bearing carbonyl functional groups. Subsequent *via* thionation of these precursors yielded α-oxo thioketones (*e.g.*, compounds 76, 78, and 80), which were subsequently reacted with 2,3-dimethylbutadiene 22 (Scheme 26).^98^

**Scheme 27:**
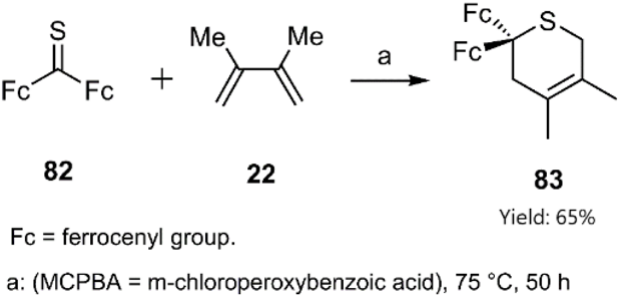
HD–A reaction of thioketone 82 and diene 22.

Mlostoń, Kowalski, and their colleagues developed a protocol for the synthesis of heterocycles integrated within multi-redox systems. These multi-ferrocenyl-functionalized organometallic structures were synthesized *via* a hetero D–A reaction between diferrocenyl-substituted thioketone 82 and diene 22 in the presence of an organic acid (Scheme 27).^99^

**Scheme 28:**
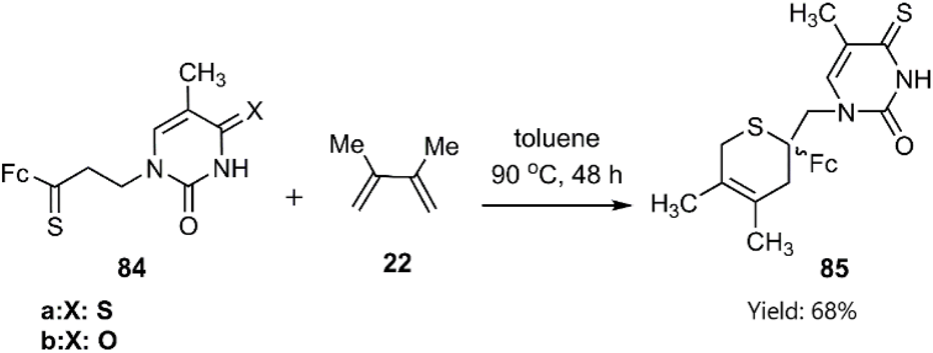
D–A reaction of thioketones 84 with diene 22.

Skiba and co-workers reported a facile synthetic route to two ferrocenyl-thymine-3,6-dihydro-2*H*-thiopyran derivatives 85, exhibiting anticancer activity, through a thio D–A cycloaddition. This approach involved the reaction of reagent 84 with diene 22 (Scheme 28).^100^

**Scheme 29:**
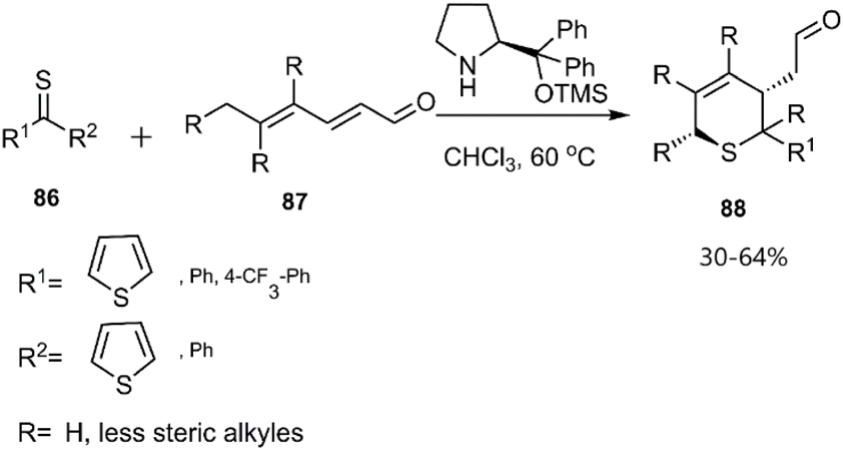
Asymmetric D–A reaction of thioketones 86 with dienals 87.

Mlostoń, Albrecht, and other coworkers reported enantioselective amine catalyzed thio D–A cycloaddition of thioketones 86 with dienales 87 (Scheme 29).^101,102^

**Scheme 30:**
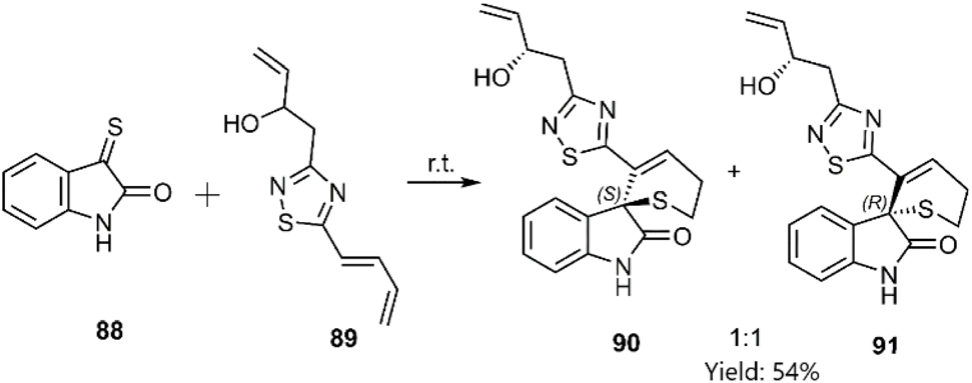
D–A reaction of 3-thioxoindolin-2-one 88 with diene 89.

Sperry research group reported a total synthesis of Alkaloids derived from isatine indigotica *via* thio D–A reaction. Insatindigothiadiazole-derived 89 as diene synthesized in several consecutive steps, starting from natural sources, and 3-thioxoindolin-2-one 88 was generated from oxindole by the sulfonation and performed *in situ* cycloaddition. The structures of the products and TSs were investigated using DFT. The interaction of π–π in the transition state has caused the high selectivity of the final product (Scheme 30).^103^

**Scheme 31:**
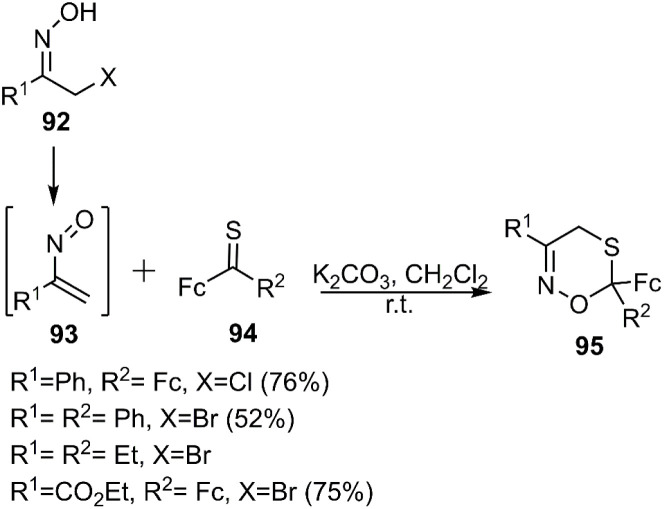
D–A reaction of ferrocenyl thiones 94 with 2-nitroalkyl-1-enes 93.

In 2018, Mlostoń's research group achieved the successful synthesis of novel oxathiazine derivatives 95, demonstrating promising potential for future applications. The presence of nitrogen, oxygen, and sulfur atoms within their structure renders these compounds attractive candidates for development as novel ligands. This synthetic pathway involved the cyclization of ferrocenyl thiones 94 with 1-nitroso-1-arylethene derivatives 93, originating from α-halogenacetophenone oxime substrate 92 (Scheme 31).

**Scheme 32:**
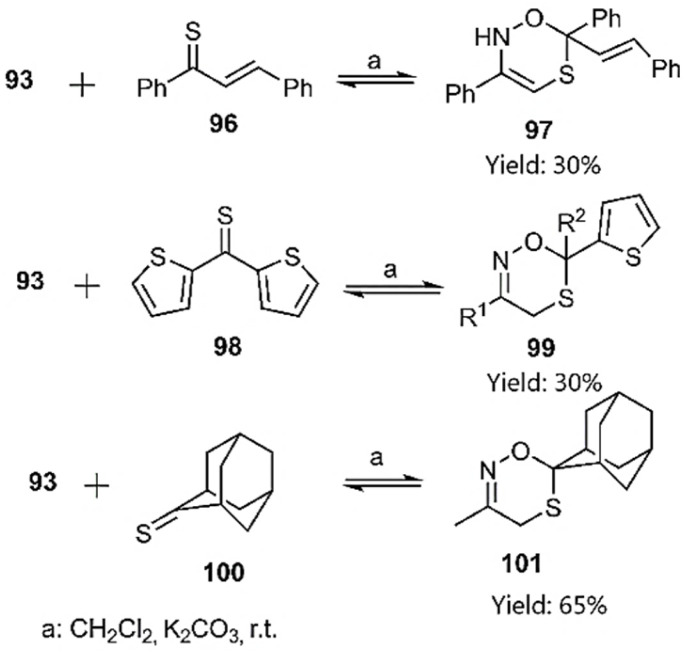
Synthesis of the oxathiazine derivatives *via* D–A reactions.

To demonstrate the versatility of this methodology, the reaction was further investigated with a range of thioketones, including thiochalcones 96, 5-(thiophene-2-carbonothioyl)thiophen-2-ylium 98, and adamantine-2-thione 100 (Scheme 32).^104^

**Scheme 33:**
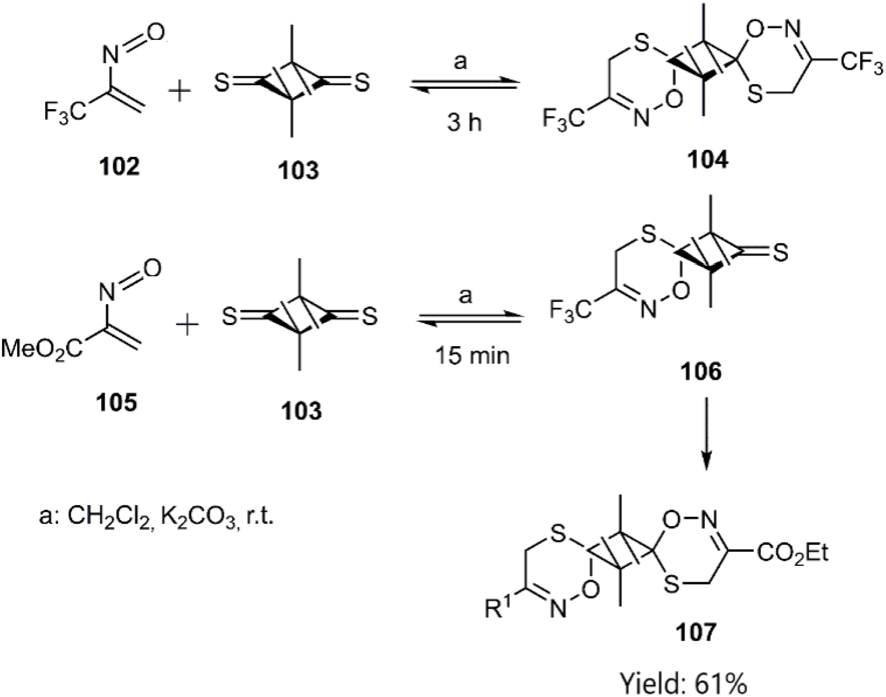
D–A reaction of 2-nitroalkyl-1-enes with thiodienophile 103.

The scope of this reaction was further expanded by employing α-nitrosoalkenes 102, 105 and thioxo cyclobutanone derivative 103. Symmetrical products 107 were synthesized *via* a novel double hetero D–A reaction between dithione 103 and α-nitroso alkene 105. Mechanistic studies revealed that steric effects govern the reaction pathway, with *trans*-selectivity favoured due to minimized steric hindrance. Notably, all reactions proceeded under mild conditions (Scheme 33).^105^

**Scheme 34:**
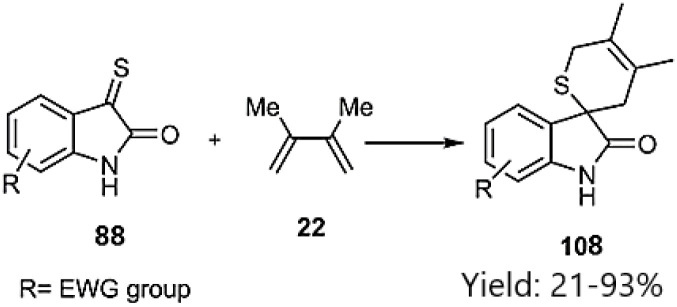
D–A reaction of 3-thioxoindolin-2-one 88 with diene 22.

Zhou *et al.* reported the utilization of carbonyl sulfide for the organocatalytic sulfonation of isatine derivatives. *In situ* generated thioxoindolin-2-ones 88 underwent thio D–A reactions with diene 22, leading to the formation of spiro compounds featuring indoline thiopyran-2-one skeletons 108. A key aspect of this work was the judicious selection of solvent. DMSO proved to be an effective solvent, preventing the formation of dimeric products (Scheme 34).^106^

##### 1.1.2.5 *N*-Sulfinyl dienophiles

*N*-Sulfinyl dienophiles, highly reactive dipolar ionic compounds, have garnered significant attention from synthetic chemists as versatile substrates for cycloaddition reactions, particularly [3 + 2] and [4 + 2] cycloaddition reactions.^102,107,108^

**Scheme 35:**
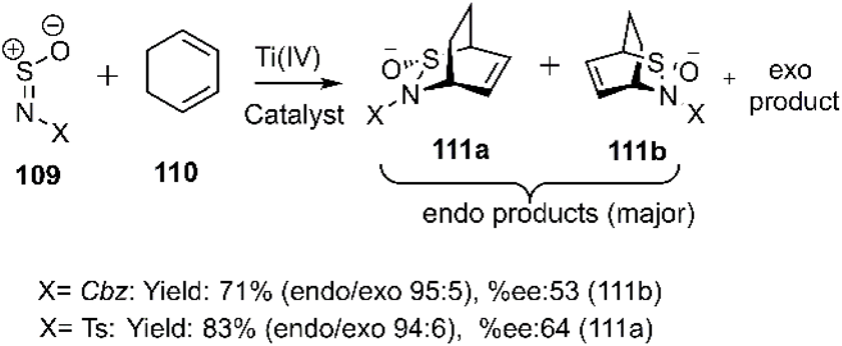
Thio D–A reaction of dienophile 109 with cyclohexadiene.

Gautun *et al.* reported efficient D–A cycloadditions employing *N*-sulfinyl compounds 109 as highly activating heterodienophiles with 1,3-cyclohexadiene 110 in the presence of chiral Ti(iv)-based Lewis's acids. These reactions afforded predominantly *endo*-isomers 111a-b with high enantioselectivity (Scheme 35). Subsequently, the same group explored the use of chiral organometallic catalysts to promote this reaction.^109,110^

**Fig. 4:**
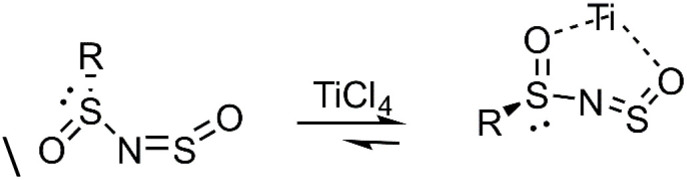
Ti^2^ chelate of *N*-sulfinyl dienophile.

Wang and coworkers developed a diastereoselective and regioselective [4 + 2] cycloaddition of dipolar *N*-sulfinyl dienophiles 109 with various dienes. A key aspect of this reaction is the ability of the substrate to chelate to Ti^2+^ or Sn^2+^, which significantly enhances the regioselectivity of the cycloaddition (Fig. 4).^111^

##### 1.1.2.6 Thiophosgene

Thiophosgene is a highly reactive and versatile precursor characterized by two labile carbon–chlorine bonds. Its toxicity arises from its role as a toxicophore, similar to those found in fungicides like Captan and Folpet. While thiophosgene hydrolyzes more slowly than its oxygen analogue, phosgene, its pronounced reactivity makes it a valuable reagent in organic synthesis. Notably, the Diels–Alder reaction offers an efficient and practical route for converting toxic thiophosgene into useful thiopyran derivatives.^112–114^

**Scheme 36:**
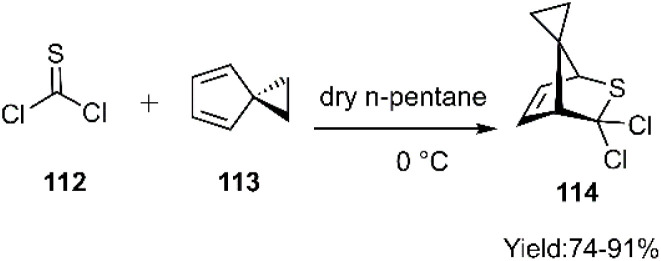
Thio D–A reaction of thiophosgene 112 and diene 113.

Föhlisch and colleagues successfully synthesized a novel thio-tricyclic scaffold through a [4 + 2] cycloaddition reaction between thiophosgene 112 and spiro[2.4]hepta-4,6-diene 113, yielding the corresponding adduct 114 (Scheme 36).^115^

**Scheme 37:**
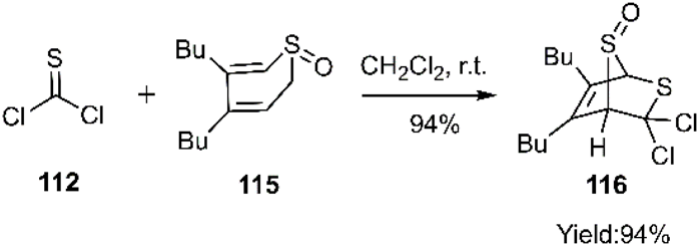
Thio D–A reaction of thiophosgene 112 to synthesis of the bicyclo structure 116.

Subsequently, Nakayama and coworkers demonstrated the utility of (*R*)-4,5-dibutyl-2*H*-thiopyran 1-oxide 115 as an efficient trapping agent in thio D–A reactions with thiophosgene 112. This reaction exhibited excellent π-face selectivity, with cycloaddition occurring exclusively from the *syn*-π-face of the diene relative to the S=O bond, leading to the formation of the *endo*-cycloadduct 116*via* attack at the less hindered face (Scheme 37).^116^

#### 1.1.3 Thio-dienes

**Fig. 5:**
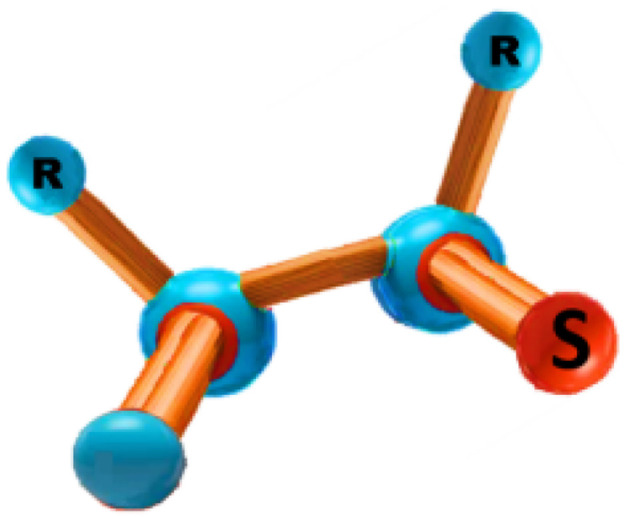
Schematic of thiodienes.

Conjugated dienes constitute an essential component of [4 + 2] cycloaddition reactions, serving either as direct reactants or as intermediates generated *in situ* (Fig. 5). Dienes can be categorized in various ways, with one primary classification based on their structural features, dividing them into cyclic and acyclic dienes.

##### 1.1.3.1 Reactions of acyclic thiodines

Acyclic thiodienes encompass a diverse range of examples, including unsaturated thioketones, thioesters, thioaldehydes, and thioamides, among others.

**Scheme 38:**
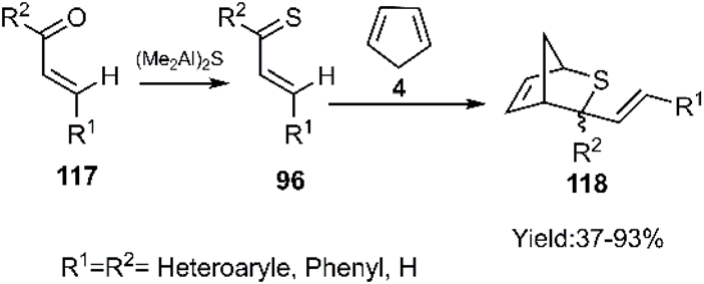
D–A of unsaturated thioketones 96.

Li *et al.* investigated the thio D–A reactions of thiochalcone derivatives 96 with cyclopentadiene (Scheme 38). Notably, the substitution pattern at R^1^ and R^2^ was found to exert spatial control over the dimerization process. For instance, when R^1^ and R^2^ are hydrogen, head-to-head dimerization is favoured. However, when R^1^ is phenyl and R^2^ is hydrogen, a preference for head-to-tail orientation is observed.^117^

**Scheme 39:**
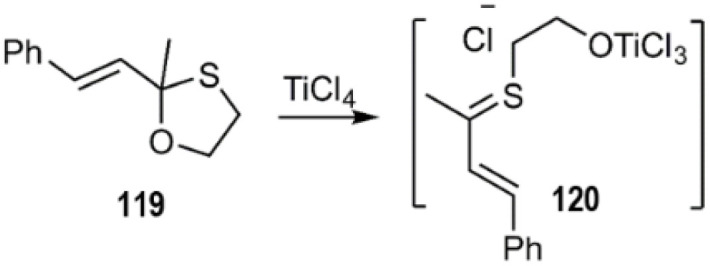
*In situ* generation of thiodiene 120.

**Scheme 40:**
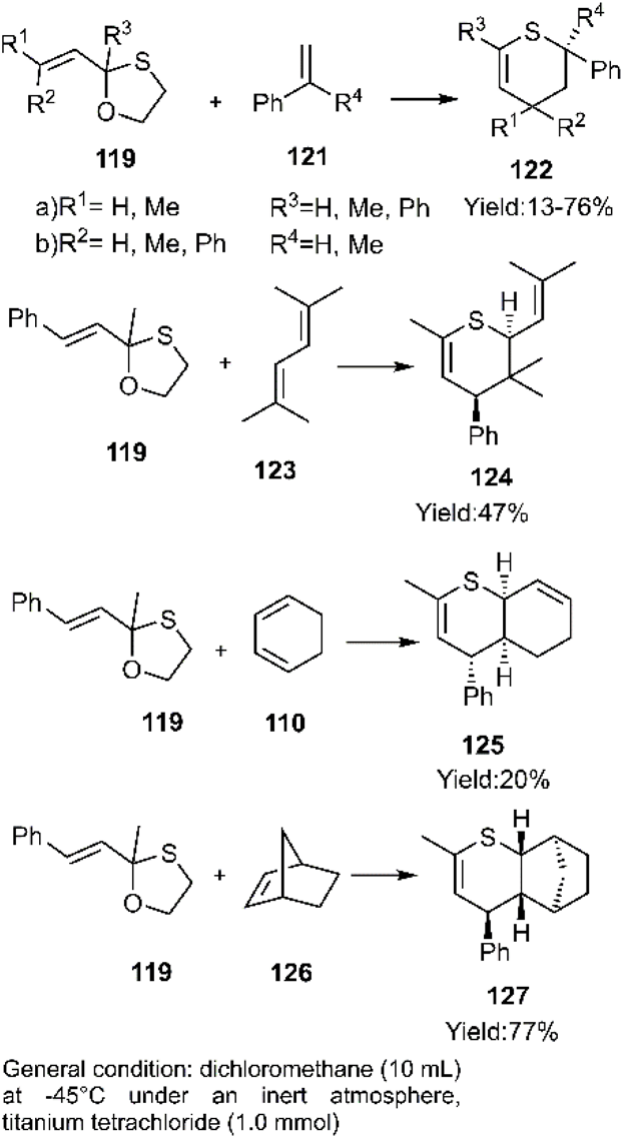
Applications of 1, 3-oxathiolane derivative 119 as masked thio diene in D–A.

D–A reactions of unsaturated 1, 3-oxathiolane derivative 117 and aliphatic and aromatic dienophiles were examined by Kerverdo and coworkers. The Lewis acidic TiCl_4_ facilitated the regioselective ring-opening of (*E*)-2-methyl-2-styryl-1,3-oxathiolane 129*via* coordination, generating a reactive thio-dienophile intermediate (Schemes 39 and 40). NOESY data indicated that the major products were *cis*-cycloadducts. Notably, the masked thioketone 119 effectively served as a thiodiene in these reactions with various alkenes, as depicted in Scheme 40.^118,119^

**Scheme 41:**
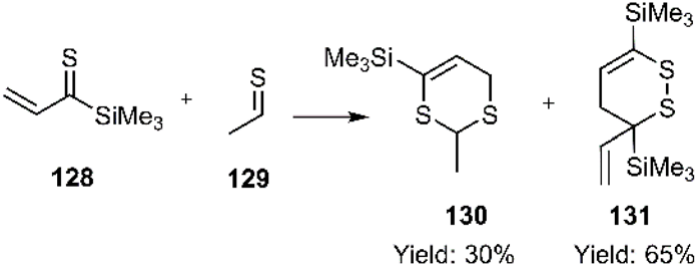
Thio D–A reaction of thio diene 128 and ethanethial 129.

According to report of Capperucci and coworkers, the cycloadducts 130 and 131 were obtained *via* the D–A reaction of thio diene 128 and ethanethial 129 which *in situ* formed from allene silanes (Scheme 41). Notably, self-dimerization of 131 was observed as the major product under these conditions.^120^

**Scheme 42:**
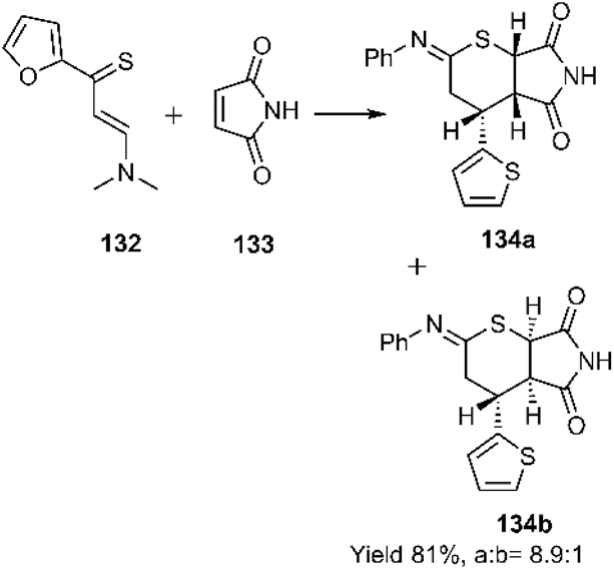
Thio D–A reaction of unsaturated amino thioketones 132 with dienophile 133.

Subsequently, Bogdanowicz-Szwed and Budzowski successfully developed a diastereoselective thio D–A reaction between the unsaturated amino thioketone 161 and 1*H*-pyrrole-2,5-dione 162 to afford tetrahydrothiopyrans 163 (Scheme 42). NOESY analysis was employed to determine the stereochemistry of the products. Interestingly, previous studies by this research group had demonstrated the ineffectiveness of this approach with EWG-substituted alkenes, including maleic acid, fumaric acid, maleimide, oxo-4-(phenylamino)but-2-enoic acid, and furan-2,5-dione.^121–123^

**Scheme 43:**
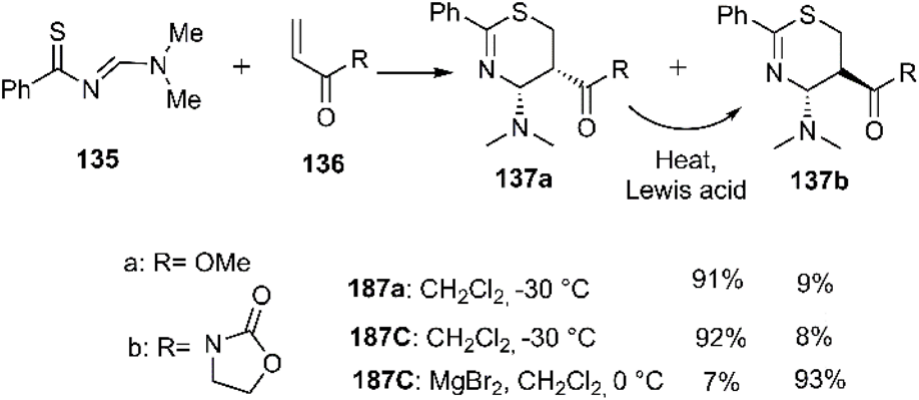
D–A reactions of benzo thioamide derivative 135.

Harrison-Marchand 's group pioneered the first asymmetric synthesis of thiazine derivatives. Their approach involved investigating the cycloaddition reaction of benzothioamide derivative 135 with dienophiles 136 under various conditions. Optimal results were achieved using samarium triflate as a catalyst. A stereoselectivity study of the cycloadducts revealed a temperature-dependent isomerization of 137a to 137b. Specifically, the *cis*-isomer underwent thermal conversion to the corresponding *trans*-isomer (Scheme 43).^124^

**Scheme 44:**
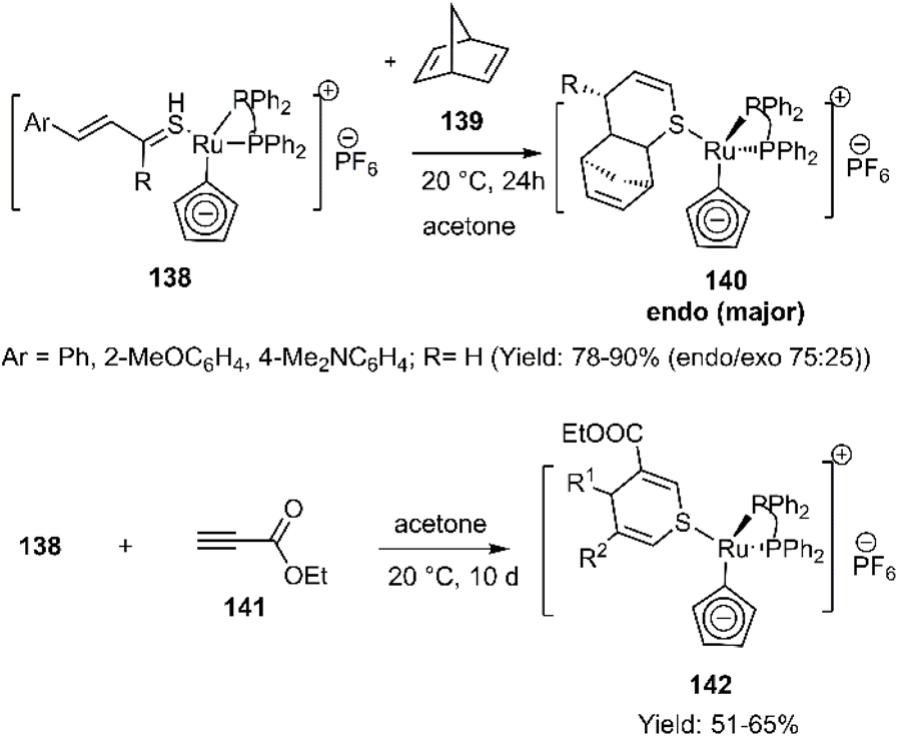
Application of ruthenium complexes of thio cinnamaldehydes 138 in D–A reaction.

Schenk *et al.* explored the stabilization of thioaldehydes within organometallic structures through coordination of the C=S group. They investigated the hetero D–A reaction of thiocinnamaldehydes, employing a strategy involving the formation of ruthenium complexes of thiocinnamaldehydes 138. Two dienophiles, norbornadiene 139 and ethyl propiolate 141, were successfully trapped by the organometallic intermediate during its formation. Steric effects were found to play a dominant role in controlling these reactions (Scheme 44).^125^

**Scheme 45:**
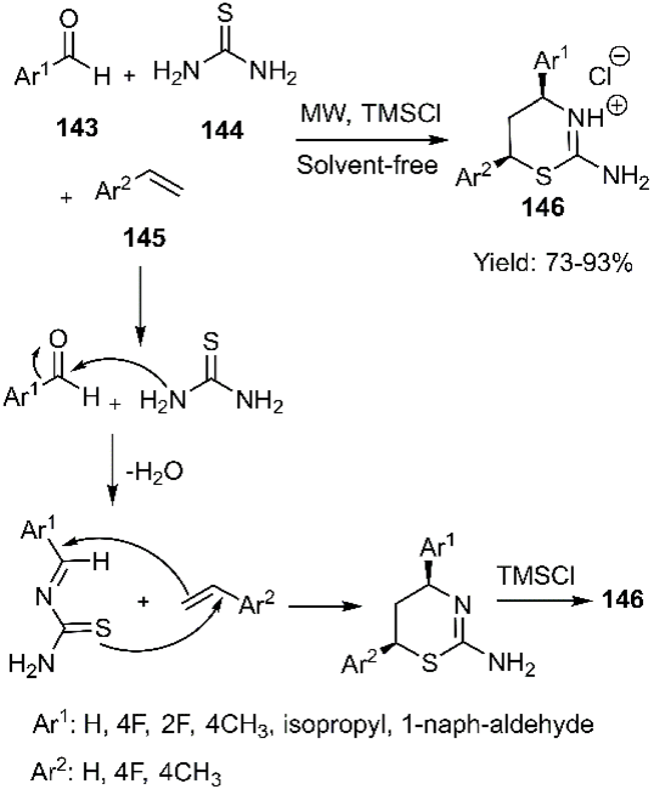
Domino condensation thio D–A reaction.

The dihydro-1, 3-thiazinume derivatives 146 were produced using microwave-promoted MCRs of aryl aldehyde 143, thiourea 144, and vinyl aryls 145 (Scheme 45). This work was reported by Wan and coworkers. In the following, it is stated that the reaction of simple cyclohexene gave no product.^126^

**Scheme 46:**
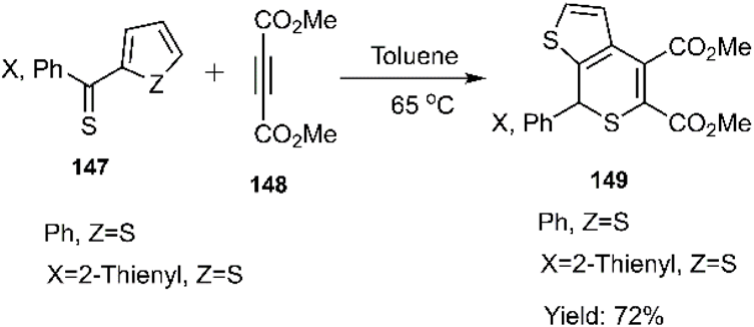
Synthesis of heterocycles 149*via* thio D–A reaction.

**Scheme 47:**
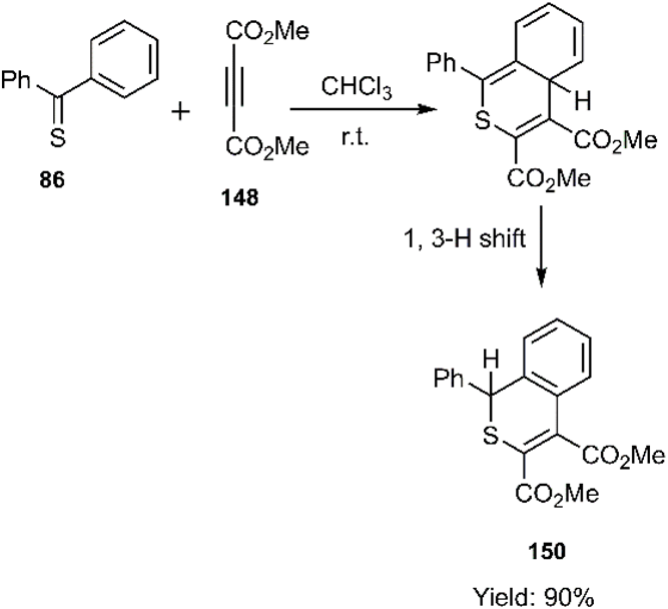
Reaction of thiobenzophenone with dimethyl but-2-ynedioate.

**Scheme 48:**
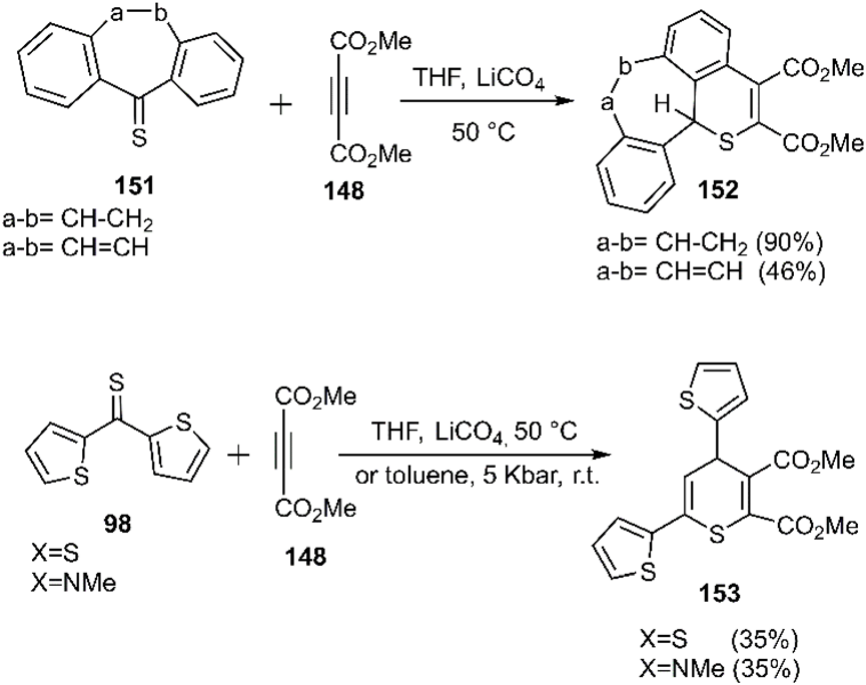
Synthesis of heterocycles 152 and 153*via* thio D–A reaction.

Mlostoń and co-workers related the synthesis of a series of polycyclic compounds through the D–A reaction of various diarylthioketones 147 with dimethyl but-2-ynedioate 148 in toluene at 65 °C (Scheme 46). This group also succeed to establish a mild reaction of thiobenzophenone 86 with dimethyl but-2-ynedioate (Scheme 47). Expanding upon the work of Mlostoń *et al.* on thio D–A reactions, this research group explored the regioselective cycloaddition of aryl thioketones 180a-b with dimethyl but-2-ynedioate 177. This reaction proceeded smoothly under the same conditions when employing thioketones bearing heteroaryl substituents. Subsequently, the cycloadducts underwent oxidation to yield the final sulfone structures. Furthermore, they successfully established a mild reaction protocol for thiobenzophenone 98 with dimethyl but-2-ynedioate (Scheme 48).^105,106,127^

**Scheme 49:**
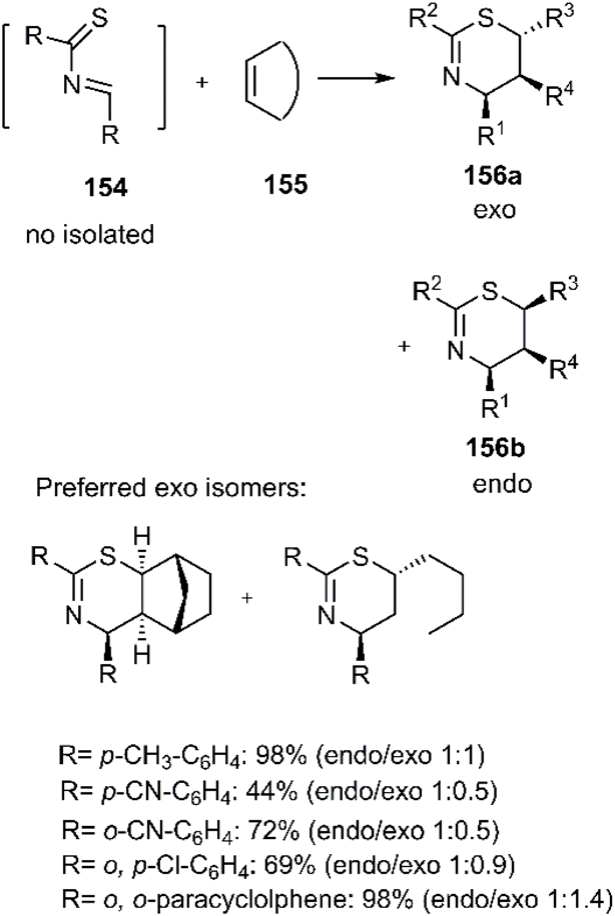
HD–A reaction to generate active optical 1,3-thiazines.

Gulea and her research team developed a microwave-assisted three-component hetero D–A reaction to synthesize optically active 1,3-thiazine derivatives 156. This innovative method involves the amination of aromatic thioamide derivatives with arylaldehydes, leading to the formation of the unsaturated thioamide 154. This intermediate is subsequently reacted with various alkenes, including norbornene, hex-1-ene, chalcone, allyl benzene, and (*Z*)-cyclooctene. The synthetic strategy was validated through comprehensive COSY, HSQC, HMBC, and NOESY experiments. The resulting products were obtained in varying *endo*/*exo* ratios, with a preference for the *exo*-approach observed in the cases of norbornene and hex-1-ene (see Scheme 49).^128–130^

**Scheme 50:**
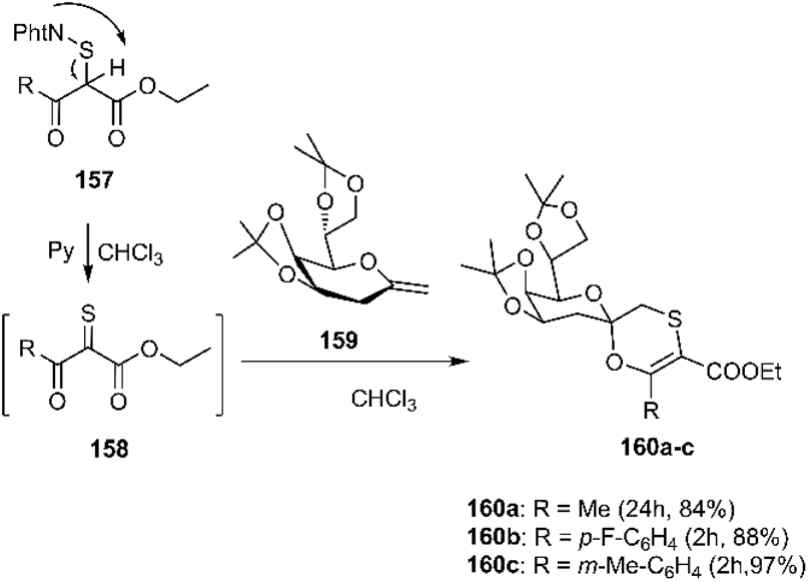
Synthesis of 2,3-dihydro-1,4-oxathiine derivatives 160.

Richichi *et al.* conducted a series of elegant studies that elucidated a stereoselective inverse electron demand [4 + 2] cycloaddition. This innovative approach involved the *in situ* generation of reactive α-dioxothiones 158, which subsequently reacted with glycosyl substrates 159, yielding KDO-based glycosyloxathiins 160 as a promising biological scaffold (see Scheme 50).^131^

**Scheme 51:**
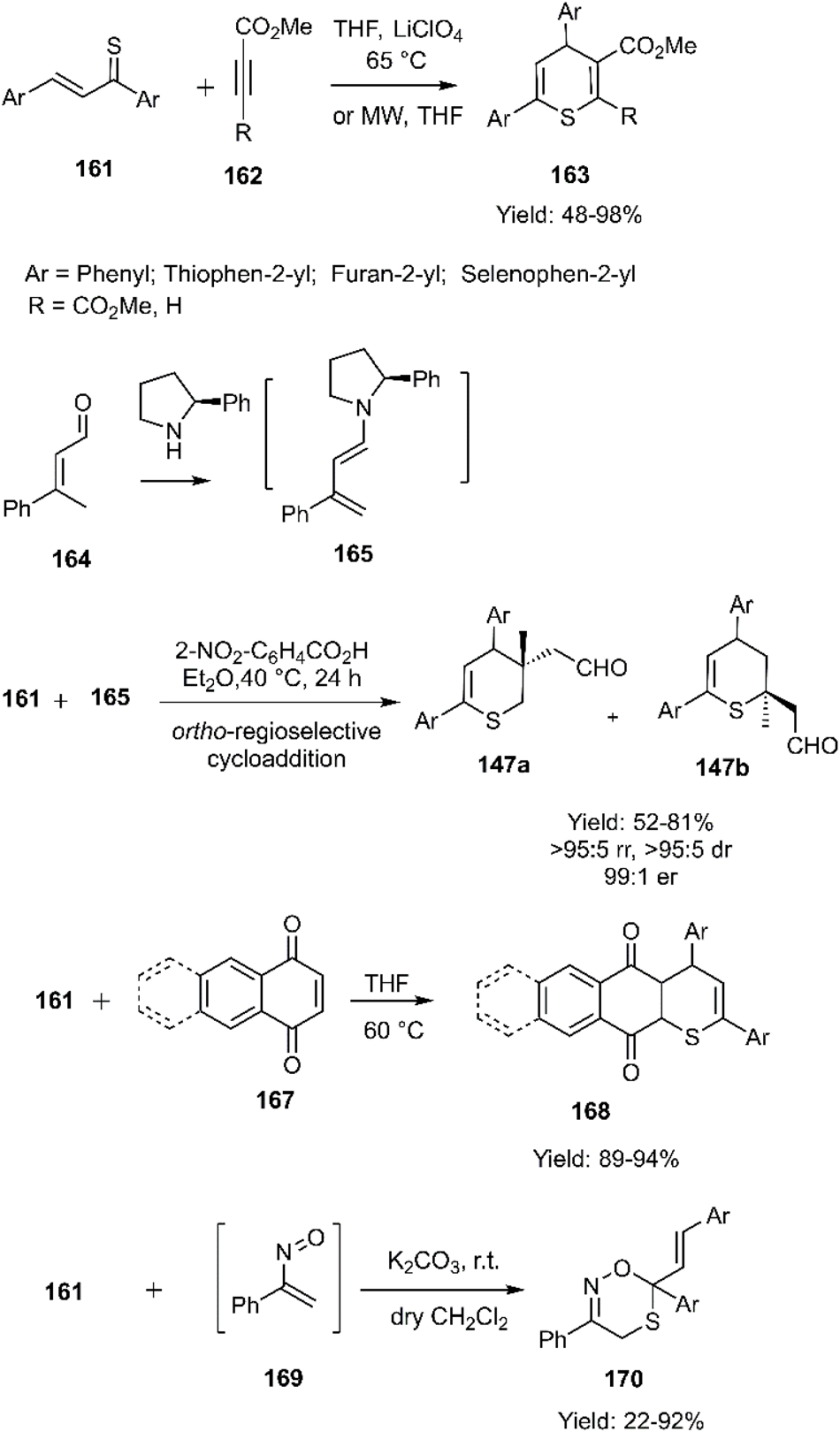
Thio D–A reaction thiochalcones 161.

Mlostoń's group investigated the cycloaddition of thiochalcone derivatives 161 with acetylene carboxylates 162. These reactions were explored under two distinct conditions: in the presence of LiClO_4_ in THF at 65 °C and under microwave irradiation. Notably, microwave irradiation led to enhanced yields and a significant reduction in reaction time compared to conventional heating. Subsequently, Mlostoń, Albrecht, and colleagues^101^ explored the synthesis of bioactive compounds *via* an asymmetric inverse-electron-demand thio D–A reaction of the thiochalcone derivatives with electron-deficient alkenes 165 as reaction partners, affording the desired products in excellent yields. The results demonstrated a high degree of *ortho*-regioselectivity, a phenomenon previously unreported in the literature. In 2018, an unprecedented synthesis of thiochromenedione derivatives 168 was achieved *via* a D–A reaction. Notably, the anticipated oxo D–A reaction pathway was not observed. The proposed mechanism identified intramolecular hydrogen bond formation in the enol form of 1, 4-quinones, which amplified the electrophilicity of the C (3) position and accelerated the reaction. Then, they investigated the cycloaddition of α-nitroso alkene 169 with thiochalcones. Despite the possibility of eight isomeric products, the reaction exclusively produced 170, incorporating all three heteroatoms within the ring. A combination of experimental evidence and theoretical insights suggests that the reaction proceeds under kinetic control (See all three cases respectively in Scheme 51).

**Scheme 52:**
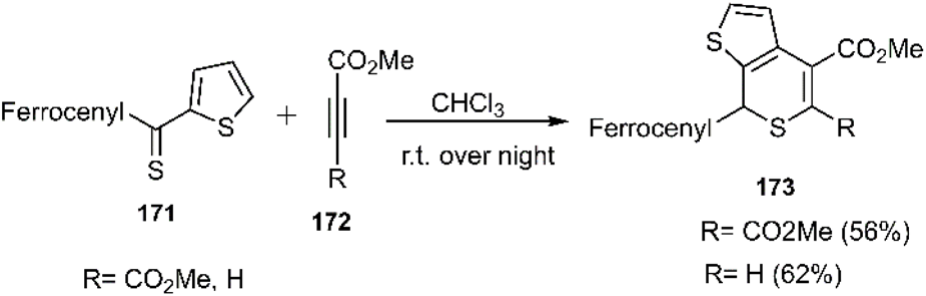
Thio D–A reaction of heteroaryl thine bearing ferrocenyl 171 with dienophile 172.

In continuation of Mlostoń's research in 2018, the scope and versatility of thiodienes were broadened under conventional conditions through the utilization of heteroaryl thiones bearing a ferrocenyl moiety 171 affording products with perfect regioselectivity. Importantly, attempts to synthesize isomeric thiochalcones by altering the aryl group at the C (1) position and the ferrocenyl (Fc) group at the C (3) position proved unsuccessful (Scheme 52).^101,104,105,132^

**Scheme 53:**
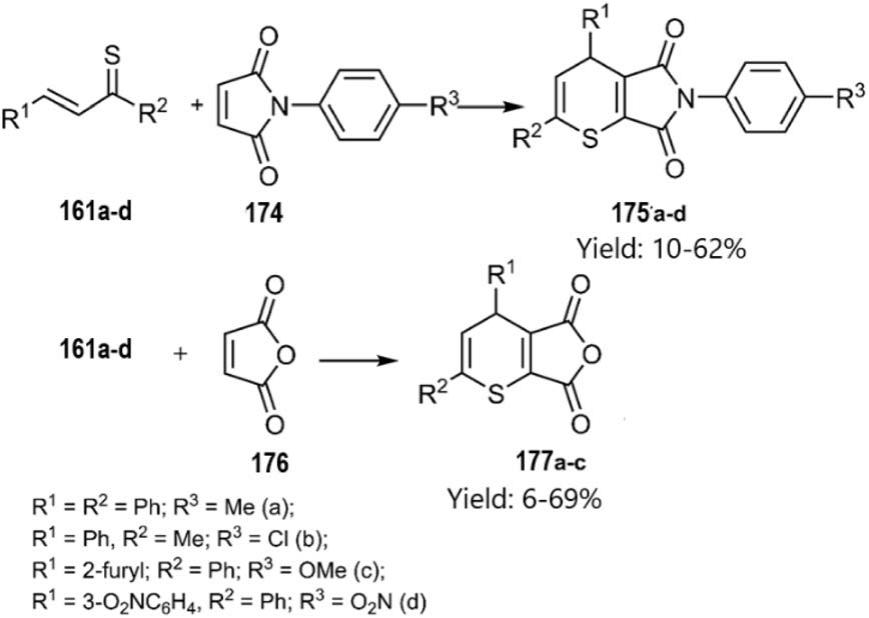
Hetero-D–A reaction of *in situ* thiochalcones 186.

**Scheme 54:**
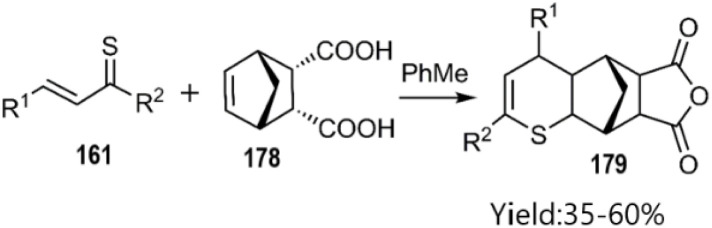
Thio D–A reaction of *in situ* thiochalcones 161.

Merkulova *et al.* extended the scope of hetero-D–A reactions by employing *in situ* generated thiochalcones 161a–d as thio dienes in reactions with *N*-aryl maleimides 174 and furan-2,5-dione 176, leading to the synthesis of thiopyran heterocycles. Lawesson's reagent served as a sulfurization agent for α,β-unsaturated ketones, enabling the *in situ* generation of thiochalcones, which acted as active thiodienes in the D–A reaction (Scheme 53) According of their next report, catalyst-free thio D–A cycloadditions of *in situ* generated thiochalcones with itaconic, maleic, and 5-norbornene-2,3-dicarboxylic anhydride 178 in organic media afforded novel dihydrothiopyran scaffolds in excellent yields. Spectral data confirmed the predominance of the *exo*-product in the cycloaddition with the norbornene derivative (Scheme 54).^133,134^

**Scheme 55:**
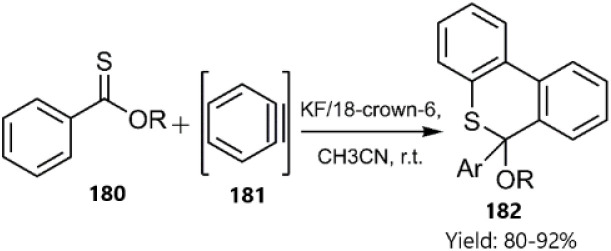
Thio D–A reaction/aromatization of aryl thionoesters 180.

Du *et al.* described a tandem thio D–A reaction/aromatization strategy for the synthesis of 6*H*-benzo[*c*]thiochromene derivatives 182 without the need for a metal catalyst. The proposed mechanism involves the reaction of aryl thionoester derivatives 180 as dienes with *in situ* generated benzyne 181 (Scheme 55).^135^

**Scheme 56:**
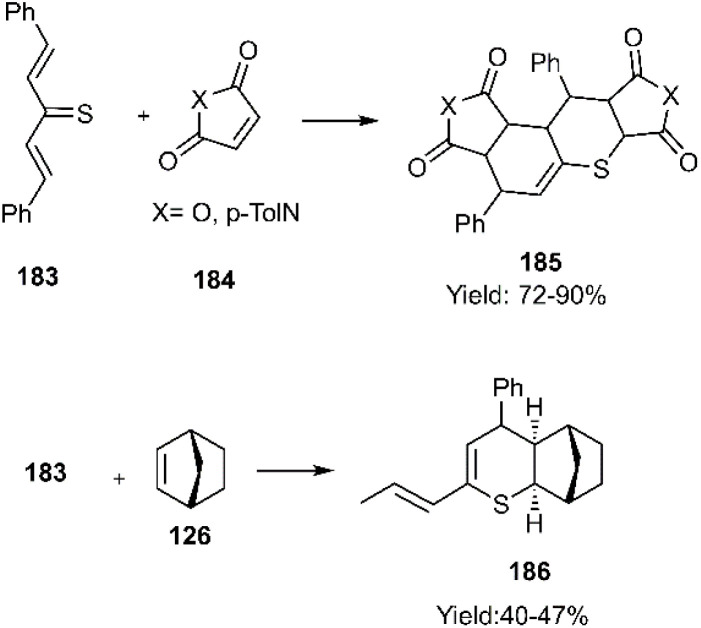
Double D–A reaction of unsaturated thio diene 183.

In 2022, the Merkulova group reported a double *in situ* thio D–A reaction employing a 1,4-diene-3-thione derivative 183 with various dienophiles to construct a series of fused polycyclic structures based on thiopyran. The electronic nature of the dienophile proved crucial, as the use of electron-donating dienophiles resulted in a single cycloaddition with moderate to low yields. When norbornene 126 was employed as the dienophile, spectral data indicated a preference for the *exo*-product (Scheme 56).^136^

**Scheme 57:**
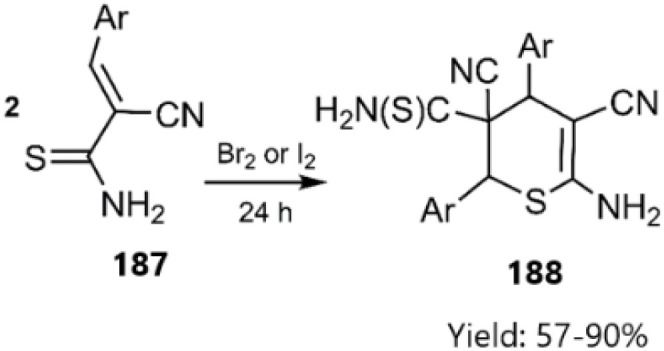
Synthesis of dihydro-2*H*-thiopyran-3-carbothiamides 188.

Recently, Dotsenko's research group unveiled the synthesis of executable access dihydro-2*H*-thiopyran-3-carbothiamide derivatives 188*via* a thio D–A reaction of (*E*)-2-cyano-3-phenylprop-2-enethioamide 187 During this process, the *E*-isomer of the Knoevenagel product 215 underwent dimerization through cycloaddition in the presence of iodide or bromide (see Scheme 57).^137^

**Scheme 58:**
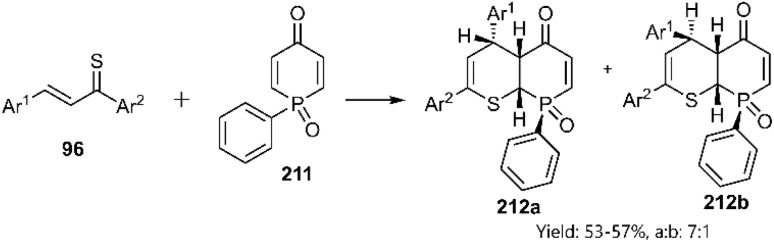
Synthesis of trihydrophosphinino[2,3-*b*]thiopyran-5-one 8-oxide derivatives 190.

In a groundbreaking project, Mlostoń's research team endeavoured to synthesize rare structures from thiopyran scaffolds. They successfully prepared a *P*,*S*-cycloadduct exhibiting an *endo*-attack preference through a thiophilic D–A cycloaddition involving thiochalcones 96 and phosphinine 1-oxides 189 (see Scheme 58).^138^

**Scheme 59:**
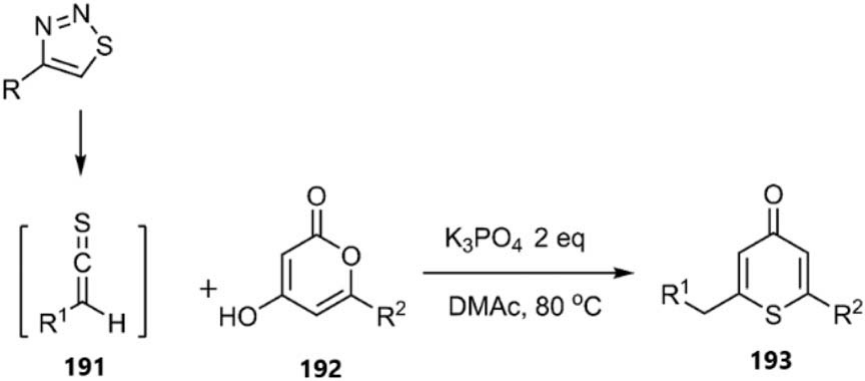
Thio D–A reaction of *in situ* generated alkene-1-thiones 213 with 4-hydroxy-2-pyrones 214.

In 2024, Huang *et al.* introduced an innovative thio D–A process. Utilizing a pentagonal 1,2,3-thiadiazole ring intermediate, they generated *in situ* alkene-1-thiones 191 as vibrant thiodienophiles. These compounds underwent thio D–A cycloaddition with 4-hydroxy-2-pyrones 192 in dimethylacetamide solvent, resulting in the formation of 2-methyl-6-(4-(trifluoromethyl)benzyl)-4*H*-thiopyran-4-one 193 (see Scheme 59).^139^

##### 1.1.3.2 Cyclic thio dienes

These compounds typically consist of unsaturated derivatives of notable thiocycles, including thiochromenes, indoline-2-thiones, *o*-thioquinones, and thiazolones. Alternatively, they may represent unsaturated transition state formed *in situ* from unstable precursors.

**Scheme 60:**
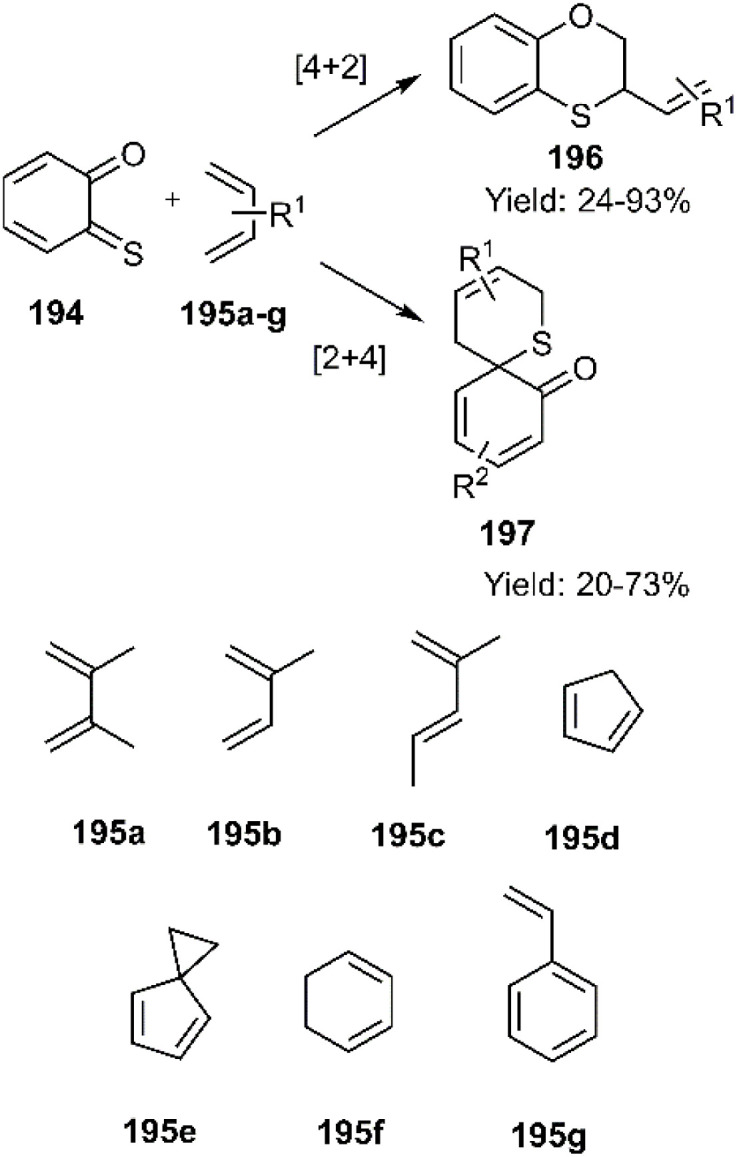
[4 + 2] and [2 + 4] cycloadditions of *o*-thio quinones (*o*-TQ) 194 in both the role of diene and dienophile 195a–g.

Menichetti and coworkers have investigated stereospecific inverse electron demand D–A reactions of *o*-thio quinones (*o*-TQs) 194 with various trapping agents containing cyclic and acyclic dienes 195a–g, over the years consecutively. Interestingly, this research team has introduced (*o*-TQs) in both the role of diene and dienophile which are named [4 + 2] and [2 + 4] cycloadditions respectively. In Scheme 60, an illustrative example is given for this purpose (Scheme 60).^140–142^

**Scheme 61:**
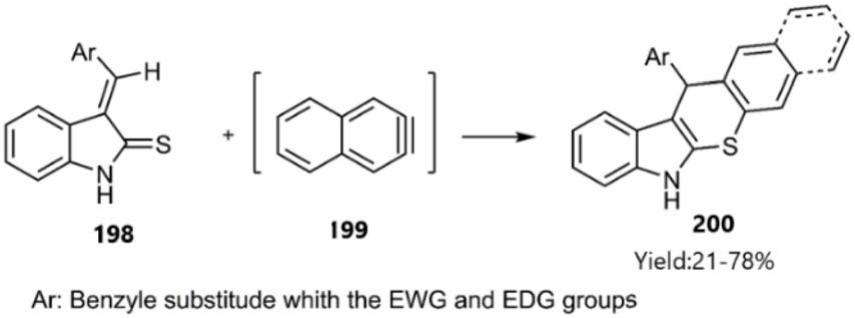
Thio D–A reactions of unsaturated indoline-2-thione 198 and arynes 199.

Biehl and coworkers earned an available π-bond to participate in the Knoevenagel-thio D–A reaction by synthesizing arynes *in situ*. This approach allows for the use of various C=S functionalized molecules, such as unsaturated indoline-2-thione 198, in the synthesis of fascinating polycyclic structures (Scheme 61).^143^

**Scheme 62:**
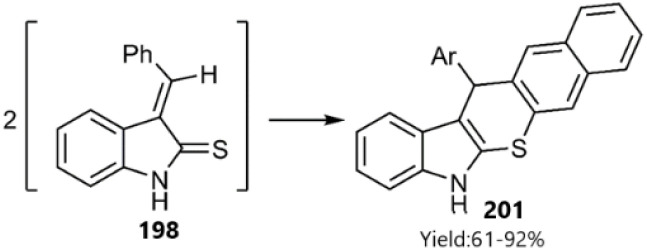
Formation of functionalized dihydrothiopyran 201.

Research conducted by Matloubi Moghaddam 's team revealed that Knoevenagel-thio D–A dimerization facilitated the catalyst-free synthesis of functionalized dihydrothiopyran 201 in an aqueous medium. The self-cycloaddition of unsaturated indoline-2-thione 198 was rationalized through resonance interactions involving the C=S bond and the nitrogen lone pair, with the unsaturated position acting as the dienophile in the corresponding substrate (Scheme 62).^144^ In a subsequent report, this group explored the reaction of unsaturated indoline-2-thiones with fullerene, resulting in a macromolecular D–A reaction that effectively functionalized the fullerene.^145^

**Scheme 63:**
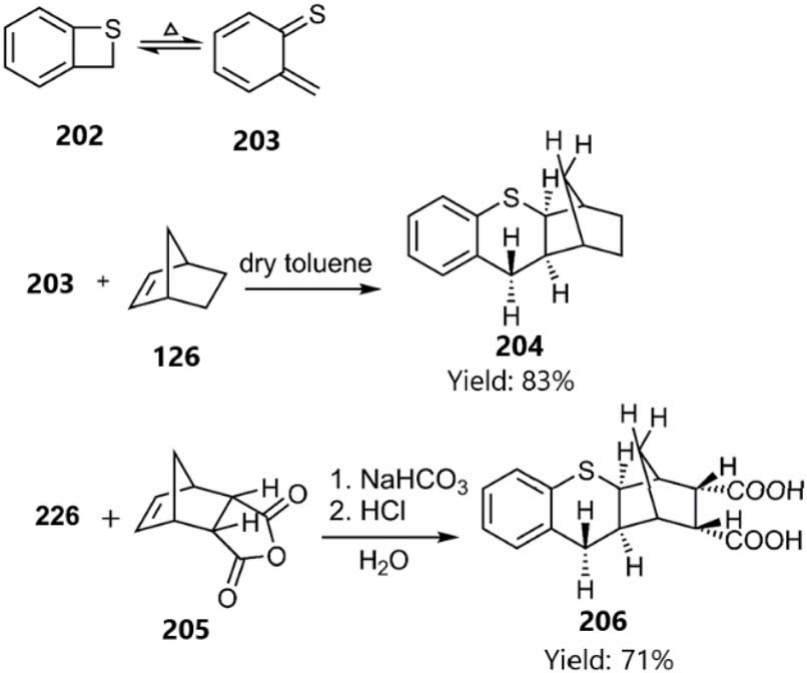
D–A reactions of 6-methylenecyclohexa-2, 4-diene-1-thione 203.

In a report by Meier and colleagues, thio bicyclooctatriene 202 was converted into 6-methylenecyclohexa-2,4-diene-1-thione 203 upon heating. Subsequently, the D–A reaction of the resulting thio diene with norbornene derivatives 126 and 205 yielded polycyclic products 204 and 206, favouring an *exo*-approach in the process (see Scheme 63).^146^ Earlier, Hegab's group had documented the regioselective reaction of indene-1-thione derivatives with 3,4-dichlorofuran-2,5-dione *via* a similar methodology.^147^

**Scheme 64:**
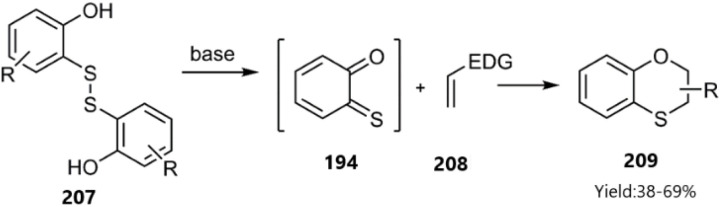
Preparation of 2, 3-dihydrobenzo[*b*][1, 4] oxathiines 209.

Viglianisi's group successfully synthesized *o*-TQs 194 as heterodienes *in situ*, leading to the formation of biologically active 2,3-dihydrobenzo[*b*][1,4]oxathiines 209. Notably, these heterocycles exhibit sweetness significantly surpassing that of sucrose and possess antioxidant properties (see Scheme 64).^148^

**Scheme 65:**
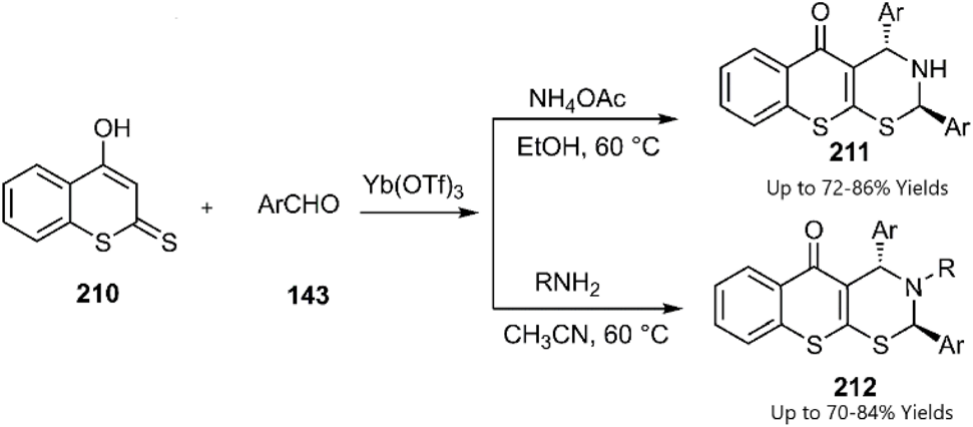
Domino reactions of 4-hydroxydithiocoumarin 210.

After that, Khan's group reported the synthesis of heterocycles 211 and 212 through a domino Knoevenagel thio Diels–Alder reaction (KTDA) (see Scheme 65). This reaction was catalyzed by Yb(OTf)_3_, demonstrating good regioselectivity as confirmed by 2D-NOE spectra. Various benzaldehyde derivatives were employed in condensation reactions with 4-hydroxydithiocoumarin 210, although reactions involving aliphatic and heteroaromatic substrates proved unsuccessful. Following this, the addition of ammonium acetate or primary amines to benzaldehyde resulted in the formation of imines, which subsequently underwent cycloaddition with the generated thio diene.^149^

**Scheme 66:**
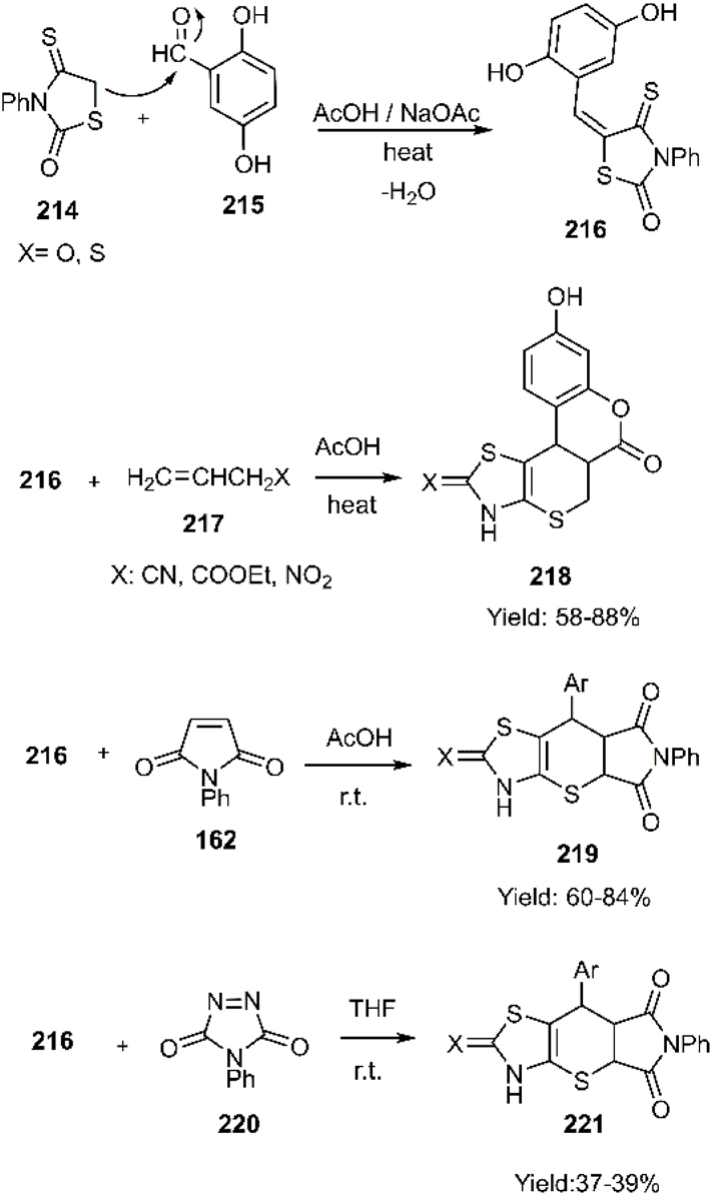
KTDA reaction of unsaturated thiazolidine-2-one derivative 216.

**Scheme 67:**
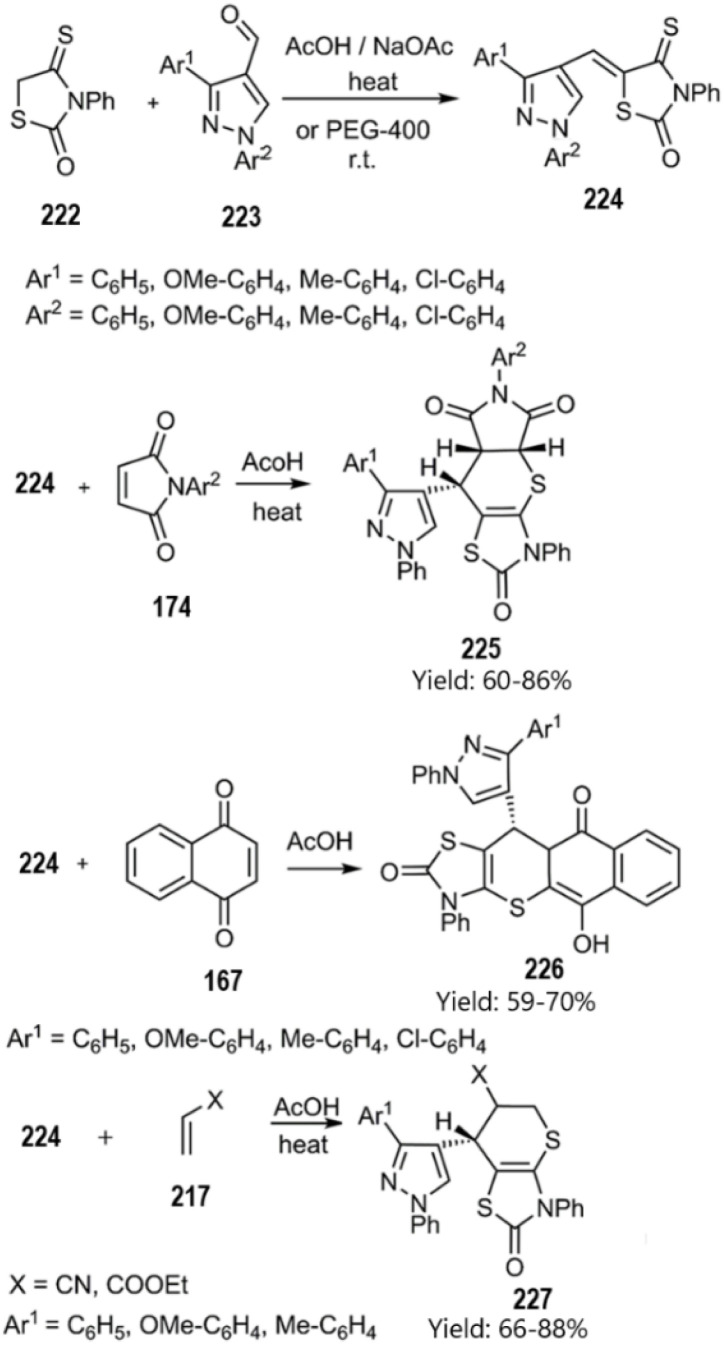
KTDA reaction of unsaturated thiazolidine-2-one derivative 224.

Metwally and colleagues successfully synthesized a series of thiopyrano[2,3-*d*]thiazole derivatives featuring a pyrazole moiety through a domino Knoevenagel thio Diels–Alder reaction. Their findings suggest that these compounds may hold significant potential in cancer treatment. According to their report, the catalyst-free KTD–A reaction of unsaturated thiazolidine-2-one derivatives 216 and 224 with various Michael acceptors, including ethyl acrylate, acrylonitrile, 3-nitroprop-1-ene 217, pyrrole-2,5-dione 162, triazole-3,5(4*H*)-dione 220, and 1,4-naphthoquinone 167 yielded the corresponding products (see Schemes 66 and 67).^150,151^

**Scheme 68:**
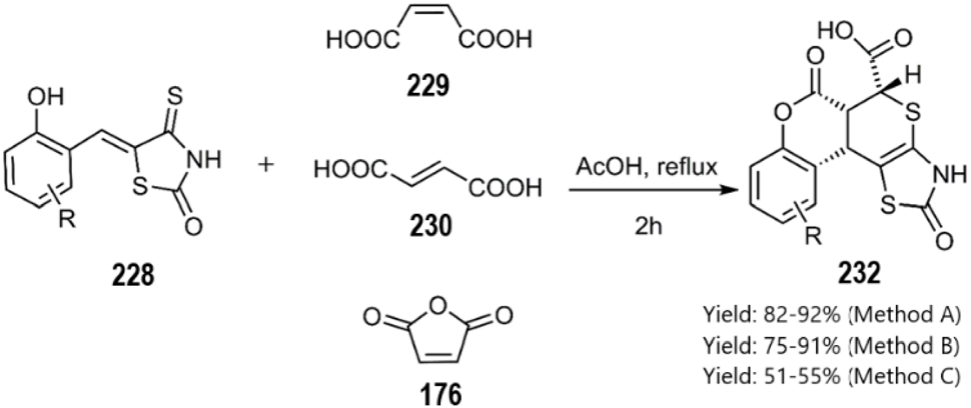
Synthesis of thiopyrano [2,3-*d*] thiazole-5-carboxylic acid bearing chromen derivatives 232.

**Scheme 69:**
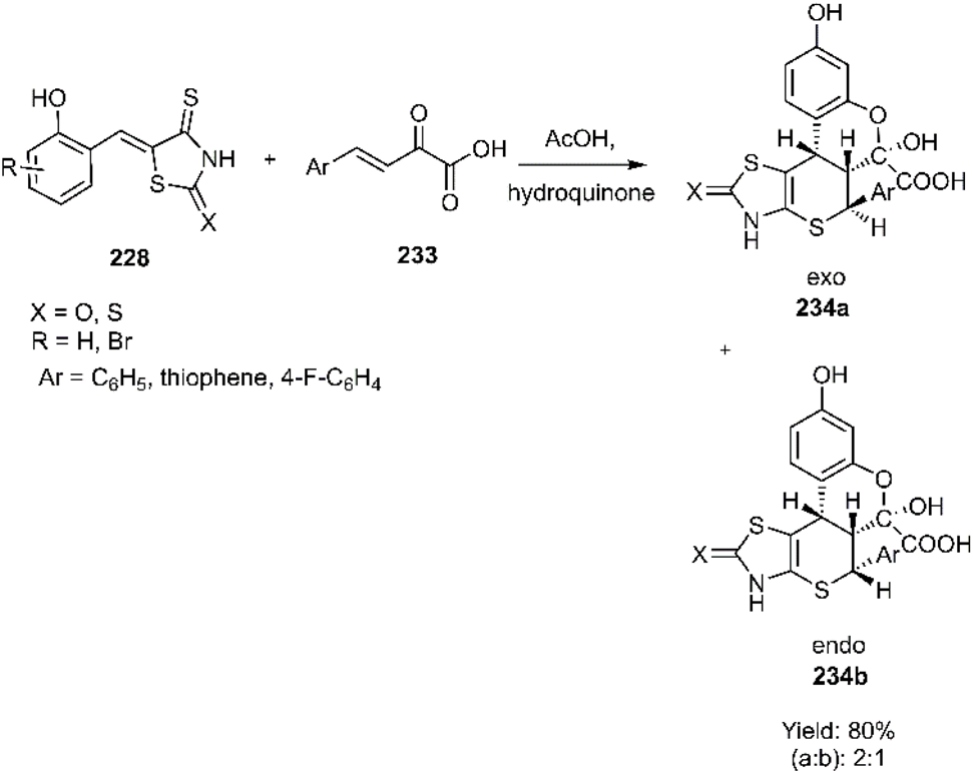
Synthesis of heterocycles based chromeno thiopyrano thiazole 234a-b.

**Scheme 70:**
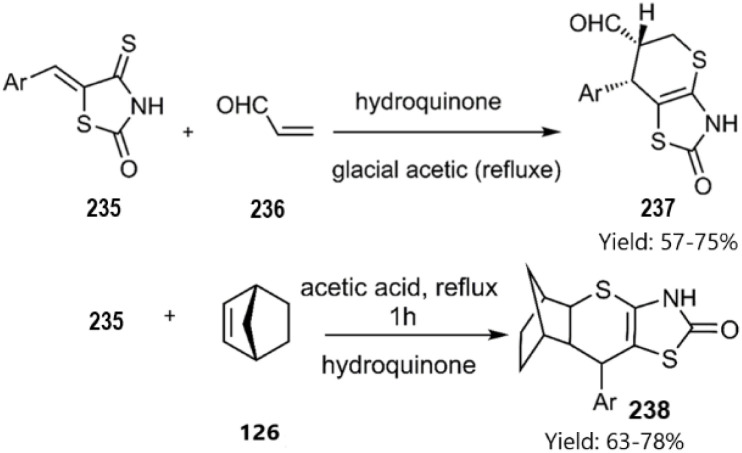
Synthesis of biological thiopyrano [2,3-*d*] thiazoles 237 and 238.

**Scheme 71:**
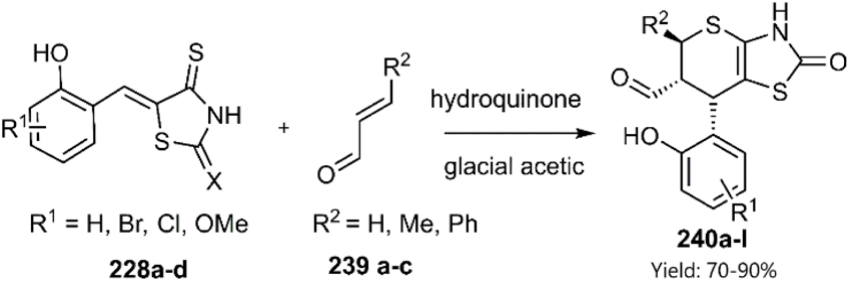
Synthesis of thiopyrane derivatives 240.

**Scheme 72:**
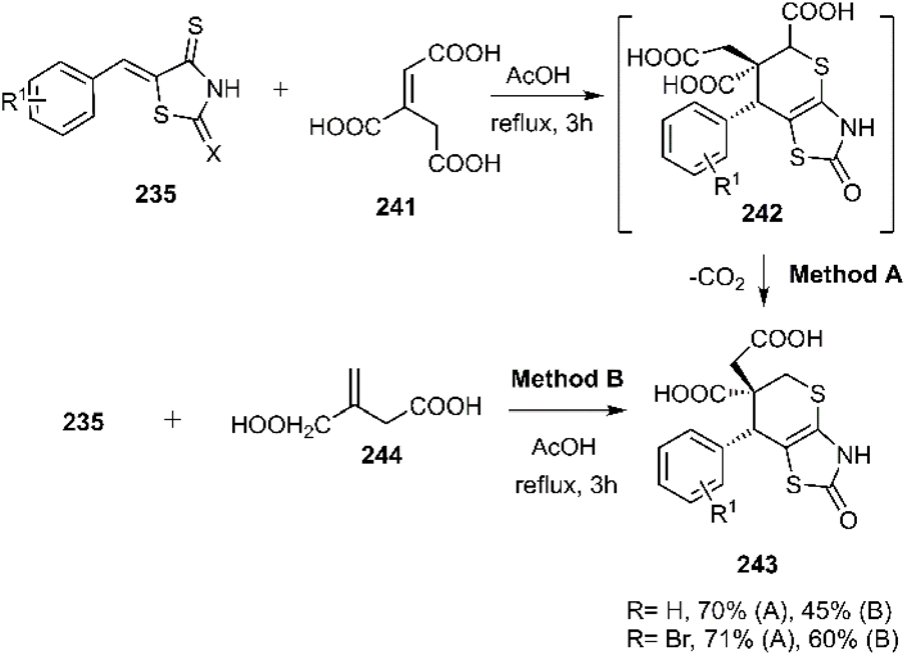
Thio D–A reactions of thioxo-2-thiazolidinones 235.

**Scheme 73:**
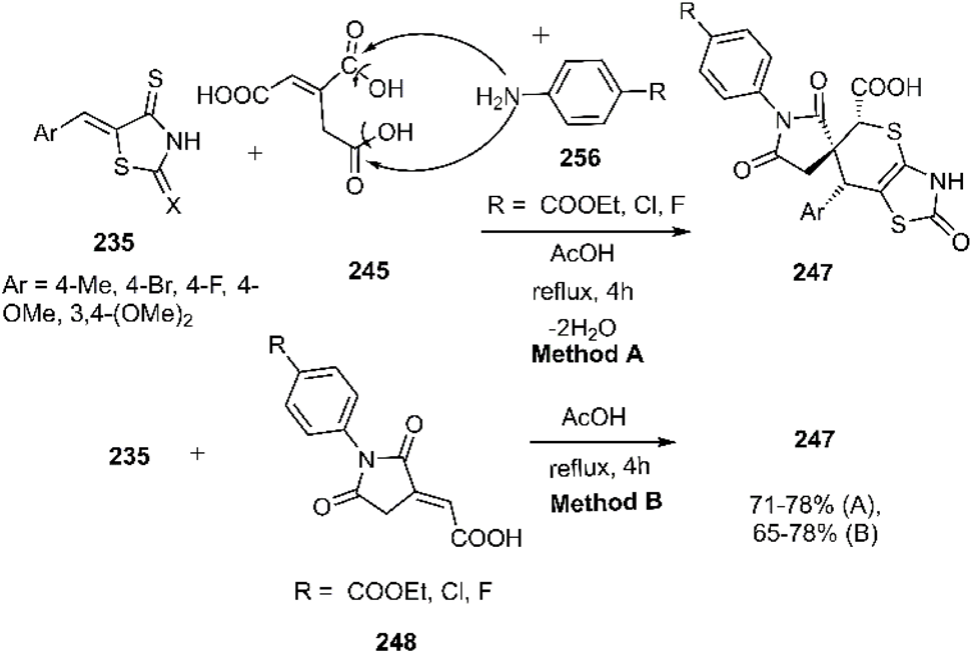
Product observed in D–A reaction of unsaturated thiazolactone 235.

**Scheme 74:**
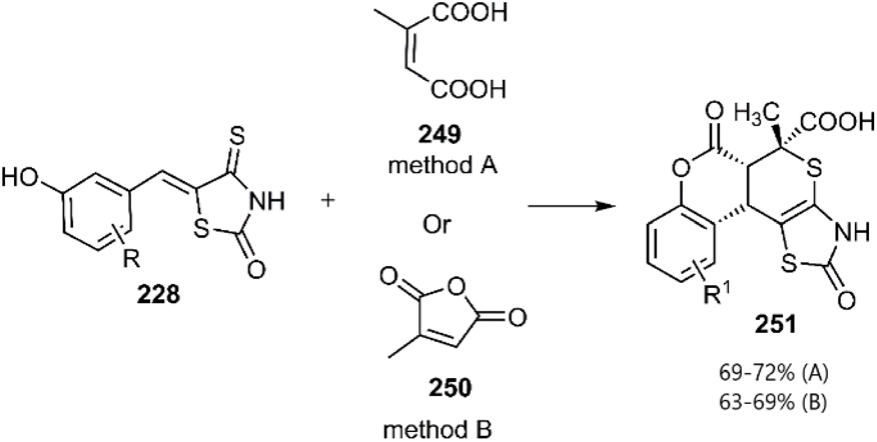
Thio D–A reaction of unsaturated thiazolactone 228.

The Lesyk team selected maleic acid 229, fumaric acid 230, and furan-2,5-dione 176 as dienophiles to engage in thio D–A reactions with EWG substituted (hydroxybenzylidene)-4-thioxothiazolidin-2-ones 228. Over the subsequent years of research on unsaturated thiazolidinones, it was noteworthy that all reactions consistently produced the same product 231, with maleic acid yielding the highest output (see Scheme 68).^152,153^ In the continuation of research on the thioxothiazolidin-2-ones by Leski and his colleagues. A series of thiopyranothiazole structures have been elegantly synthesized through a tandem acylation thio D–A reaction facilitated by hydroquinone. The heightened reactivity of the unsaturated position in (*E*)-2-oxo-4-arylbut-3-enoic acid 233 renders it an ideal dienophile, resulting in its reaction with 5-ylidene-4-thioxo-2-thiazolidinone 228 to produce a mixture of *endo*/*exo*-adducts in a diastereomeric ratio of 2 : 1 (Scheme 69).^154^ In Further research under Lesyk's supervision, they successfully synthesized anticancer and antiviral thiopyranothiazoles 237, 238 (see Scheme 70).^155–158^ In 2017, the research team led by Lesyk investigated the D–A reaction of *o*-phenolic 4-thioxo-2-thiazolidinone derivatives 228 with a variety of unsaturated aldehydes 239, as illustrated in Scheme 71.^159^ Subsequently, they investigated D–A reactions of 4-thioxo-2-thiazolidinone 235 with aconitic acid derivatives (Scheme 72).^160,161^ Also, delineated two efficient synthetic pathways for constructing thiopyranothiazole skeletons and succeeded in the synthesis of spiro-substituted thiopyranothiazoles by devising a multi-component reaction involving unsaturated thioxo-2-thiazolidinones 235, *trans*-aconitic acid 245, and aniline derivatives 246, as illustrated in Scheme 73 (Method A). Remarkably, identical products were also obtained using arylpyrrolidin-3-ylidene derivatives 265a–c (Method B).^160^ In 2021, Lesyk *et al.* reported a thio D–A reaction of unsaturated thiooxazolones 228 with citraconic acid 249, achieving high regio- and stereoselectivity followed by intramolecular lactonization. This approach yielded the same products utilizing 3-methylfuran-2,5-dione 251 as the diene, as depicted in Scheme 74.^162^

**Scheme 75:**
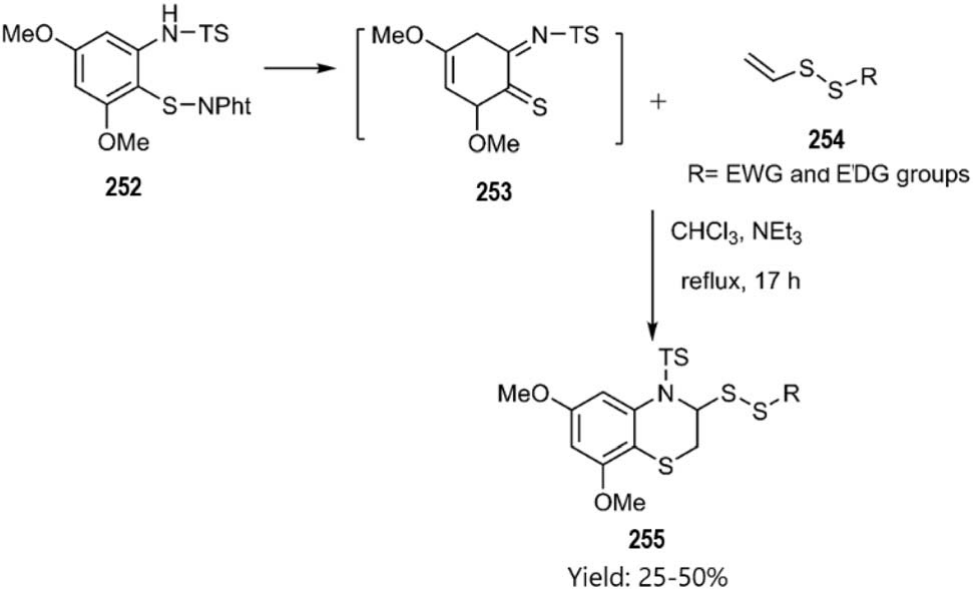
Thio D–A reaction of the derivative 253 with substituted vinyldisulfide 254.

In the same year, Witt research team conducted an investigation that led to an inverse electron-demand [4 + 2] cycloaddition. The corresponding benzo[*b*][1,4]thiazines 255 were synthesized through the cycloaddition of *o*-iminothioquinone intermediate 253 with vinyl disulfide derivatives 254 the under moderate conditions, as illustrated in Scheme 75.^163^

**Scheme 76:**
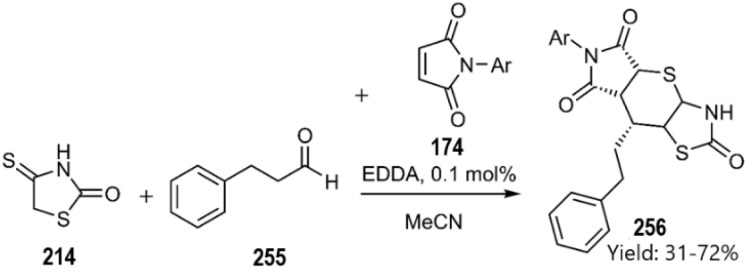
Domino-KTDA reaction in order to synthesis thiopyranothiazole derivatives 256.

In 2022, Lesyk *et al.* explored a novel multicomponent reaction involving 4-thioxothiazolidin-2-one 214 3-phenylpropanal 255 and pyrrole-2,5-dione 174. These chemical motifs were efficiently generated through a domino KTDA reaction, resulting in the successful synthesis of polycycle 256 in a single pot with commendable diastereoselectivity (Scheme 76).^164^

### 1.2 Step-wise reactions

**Fig. 6:**
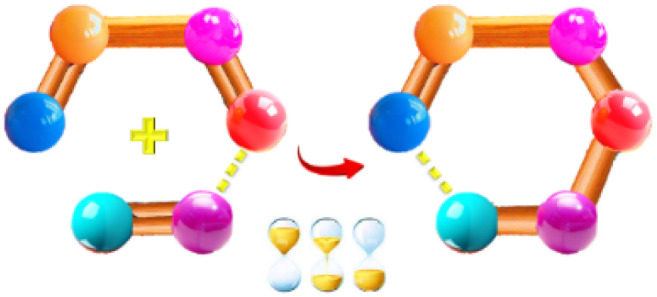
General schematic of stepwise [4 + 2] cycloaddition reactions.

While the Woodward-Hoffmann rules apply exclusively to concerted reactions, certain orbital interactions can prompt a reaction to follow a stepwise pathway, ultimately producing the allowed cycloadduct. The term “stepwise” refers to reactions that yield products through two or more sequential steps (Fig. 6). The stepwise mechanism be accomplished by passing through the of either zwitterion or diradical intermediates.^165,166^ Distinguishing between concerted and stepwise mechanisms remains a key challenge in cycloaddition chemistry, a subject that has been extensively debated by researchers. Numerous theories have been proposed to elucidate the factors governing the progression of cycloaddition reactions along single-step or stepwise pathways. Stereoselectivity has often been highlighted as a critical indicator for determining the reaction mechanism^167^ while steric hindrance can occasionally obstruct concerted pathways.^168,169^ Additionally, the polarity of reaction media to stabilize zwitterionic intermediates and the presence of specific substituents on precursors can push the mechanism toward a stepwise route.^170,171^ Computational chemistry has also provided valuable insights into distinguishing reaction mechanisms.^172^ By calculating the concert energy—defined as the activation energy difference between concerted and stepwise pathways—scientists can predict the preferred reaction pathway.^173^ These computational studies have also identified the presence of anti-transition states in two-step reactions.^67,174,175^ One proposed criterion suggests that if the formation of the second carbon–carbon bond during cycloaddition exceeds 30 femtoseconds, the reaction is classified as stepwise. This body of research underscores the intricate interplay of stereo electronic factors, substituent effects, and computational models in advancing our understanding of cycloaddition mechanisms.^176–178^ Overall, the stepwise cycloaddition exhibits lower stereospecificity than its concerted counterpart.^179^ Over the past two decades, organic chemists have made significant strides in the stepwise synthesis of thiopyran, demonstrating success in both experimental and computational methodologies.^180^

#### 1.2.1 Zwitterion mediated

Zwitterion refers to a structure with both negative and positive charge. Stepwise cycloaddition reactions predominantly proceed through zwitterionic intermediates, particularly when the reaction mechanism is governed by polar interactions. The incorporation of polar solvent or ionic liquids can facilitate the progression of the reaction pathway through these zwitterionic intermediates in a controlled manner. The stepwise mechanism involving a zwitterionic intermediate can be further elucidated by examining the sensitivity of the reaction rate to the presence of EWGs and EDGs.^165,180–185^

**Scheme 77:**
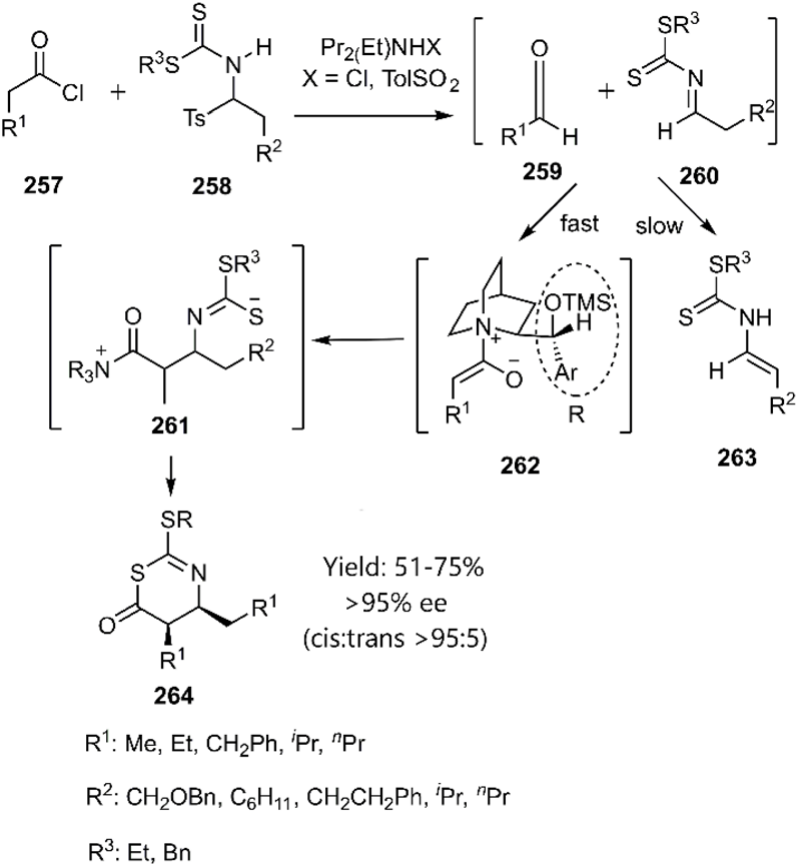
The mechanism for the stepwise [4 + 2] cycloadditions of the dithioesterter 260.

Nelson and co-workers reported an *in situ* thio D–A reaction between ketene derivatives and *N*-thioacyl imines, facilitated by an alkaloid catalyst. This catalyst promoted the reaction through coordination to the TS, stabilizing a pseudo-chain-conformer geometry 263 (Scheme 77).^186^

**Scheme 78:**
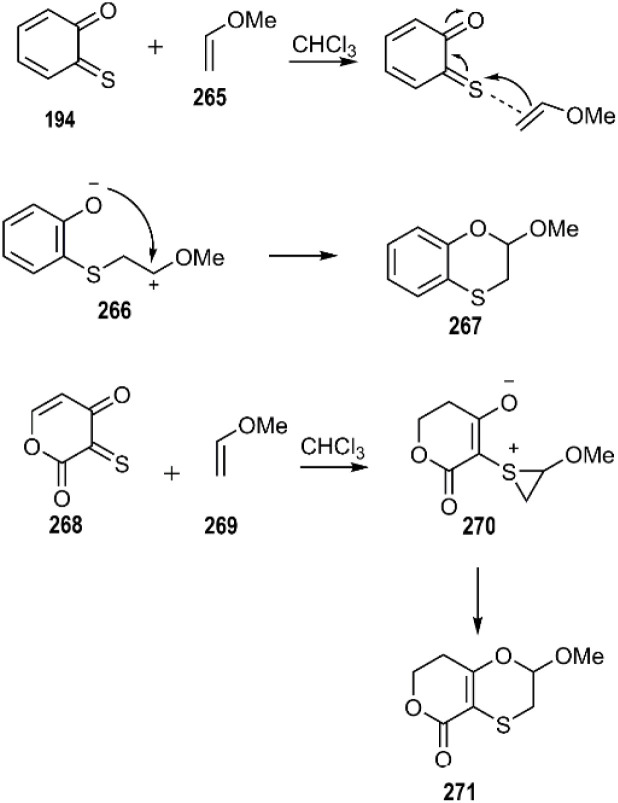
Possible mechanism of the [4 + 2] cycloaddition of α-oxothiones.

Toma and colleagues employed computational methods to investigate HD–A reaction of *o*-thioquinoline 194 with methyl vinyl ether 265. Their findings suggest a two-step mechanism involving the formation of a zwitterionic intermediate 266. Furthermore, they explored the cycloaddition of 3-thioxo-2*H*-pyran-2,4(3*H*)-dione 268 as a heterodiene, comparing its reactivity to that of *o*-thioquinolines. These studies relied on DFT calculations to elucidate the reaction pathway (Scheme 78). Notably, they identified a unique intermediate 270 along the reaction coordinate, a feature potentially attributed to the enhanced electron density on the sulfur atom.^187^

**Scheme 79:**
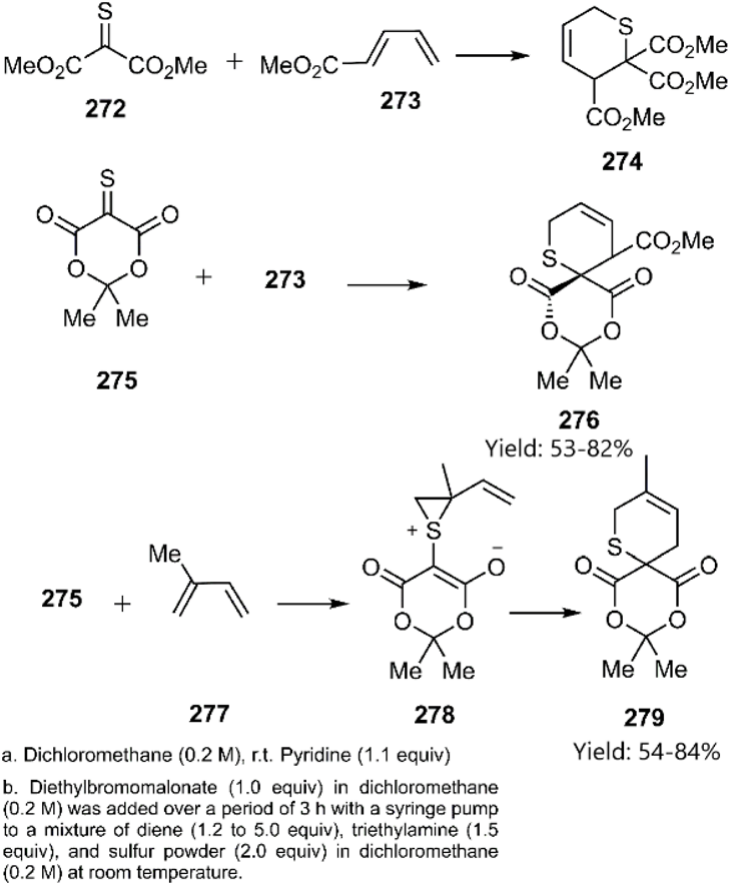
Examples of [4 + 2] cycloaddition of thio-carbonyl derivatives through zwitterionic intermediate.

Dory and co-workers investigated the mechanism and regioselectivity of the [4 + 2] cycloaddition reactions of thiono-analogs, specifically acetic 2-methoxy-2-oxoethanethioic anhydride 272 and thio-Meldrum's acid 275, with 1,3-dienes. They proposed a stepwise mechanism involving the formation of a zwitterionic intermediate 278, accessed *via* an inverse [2 + 1] cycloaddition pathway (Scheme 79).^188^

**Scheme 80:**
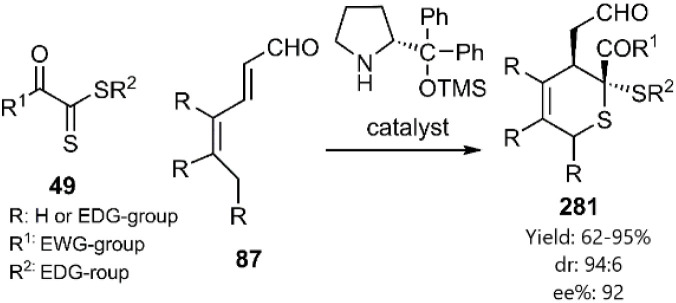
D–A reaction of dithioesters 49 with diene 87.

Jørgensen *et al.* developed a novel approach to enhance the enantioselectivity and reactivity of dithioesters 49 with hexadienal derivatives 87 through the *in situ* generation of catalyst-bound dienes. This methodology facilitates the formation of thio D–A adducts *via* a zwitterionic intermediate. The authors demonstrated that EWG-activated thiodienophiles significantly improves both diastereo- and enantioselectivity in the D–A reaction (Scheme 80).^189^

**Scheme 81:**
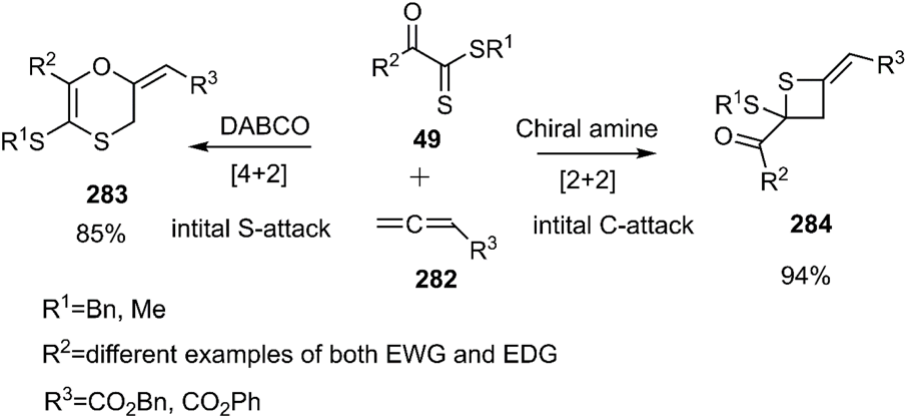
D–A reaction of allenoate 282 with dithioesters 49.

**Scheme 82:**
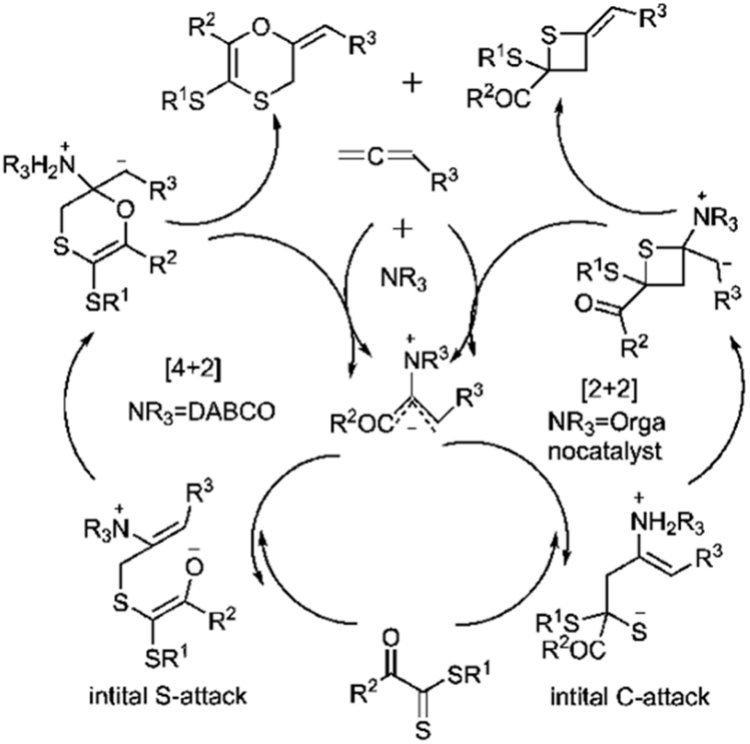
Proposed mechanism of both [2 + 2] and [4 + 2] cycloaddition.

Wei and coworkers reported the first example of a controllable and stereoselective [4 + 2] and [2 + 2] cycloaddition reaction between allenoates 282 and dithioesters 49 (Scheme 81). They demonstrated that the choice of nucleophilic amine catalyst controlled the selectivity between these two cyclization pathways. The formation of a zwitterionic intermediate enables both [4 + 2] and [2 + 2] cycloadditions, depending on the site of attack. For instance, DABCO catalysis Favors the formation of the [4 + 2] cycloadduct 283, proceeding through an initial sulfur (S)-attack. Conversely, chiral amine catalysts promote an initial carbon (C)-attack, leading to the [2 + 2] cycloaddition reaction (Scheme 82).^190^

**Scheme 83:**
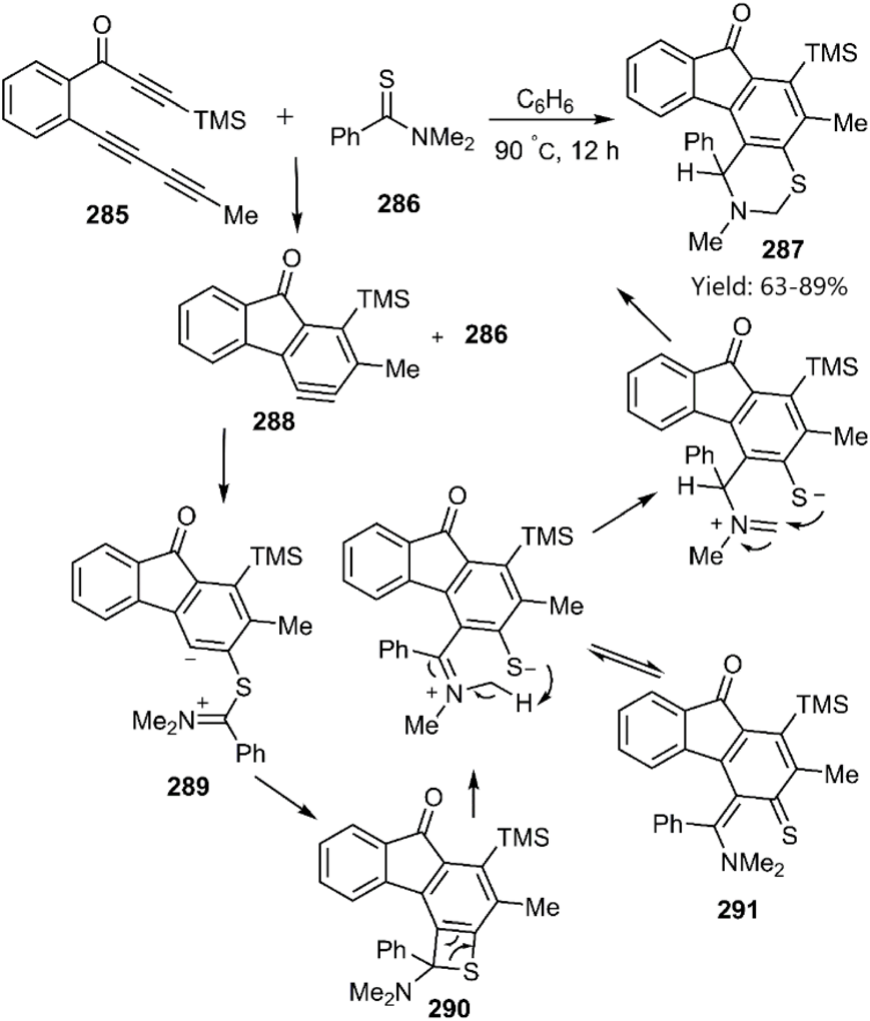
Step-wise D–A reactions of *N*,*N*-dimethylbenzothioamide 286.

Hoye and coworkers explored alternative pathways for [4 + 2] cycloaddition reactions. Their approach utilized the HDDA strategy, generating a reactive benzyne intermediate 288. Subsequent reaction of this intermediate with a thioamide derivative 286 facilitated the formation of the [4 + 2] cycloadduct through electron transfer. Initially, a competing [2 + 2] cycloaddition pathway yielded a four-membered heterocycle 290. A subsequent 1,3-hydrogen atom shift in diene 291 led to the formation of product 287 (Scheme 83).^191^

**Scheme 84:**
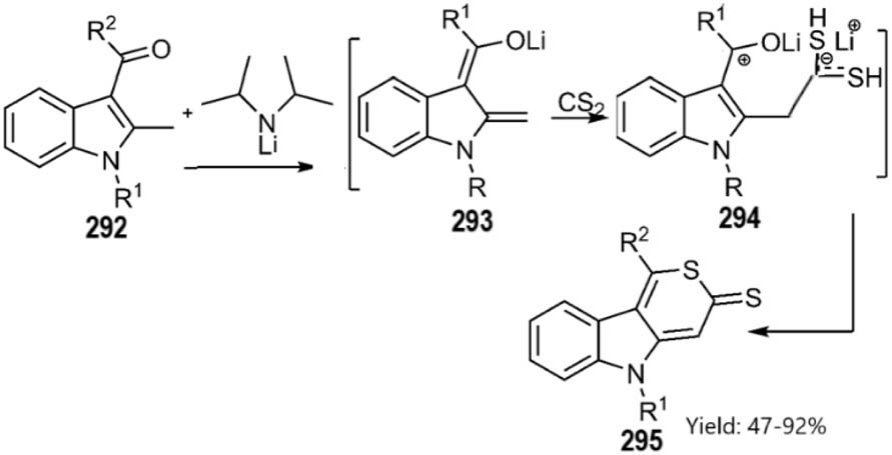
Step-wise cycloaddition to synthesis of the cycloadduct 295.

An unprecedented approach to forming important thiopyranoindolethione compounds *via* [4 + 2] cycloaddition and C–S bond formation was reported by the research team of Vyalyh. This approach used *in situ* trapping of lithium indole-2, 3-dienolate mediates 293 with carbon disulfide, followed by aromatization and the release of lithium hydroxide. The authors estimated the reaction mechanism to be stepwise cycloaddition with ion pair mediate relied on the evaluation of several possible mechanisms through DFT studies (Scheme 84).^192^

**Scheme 85:**
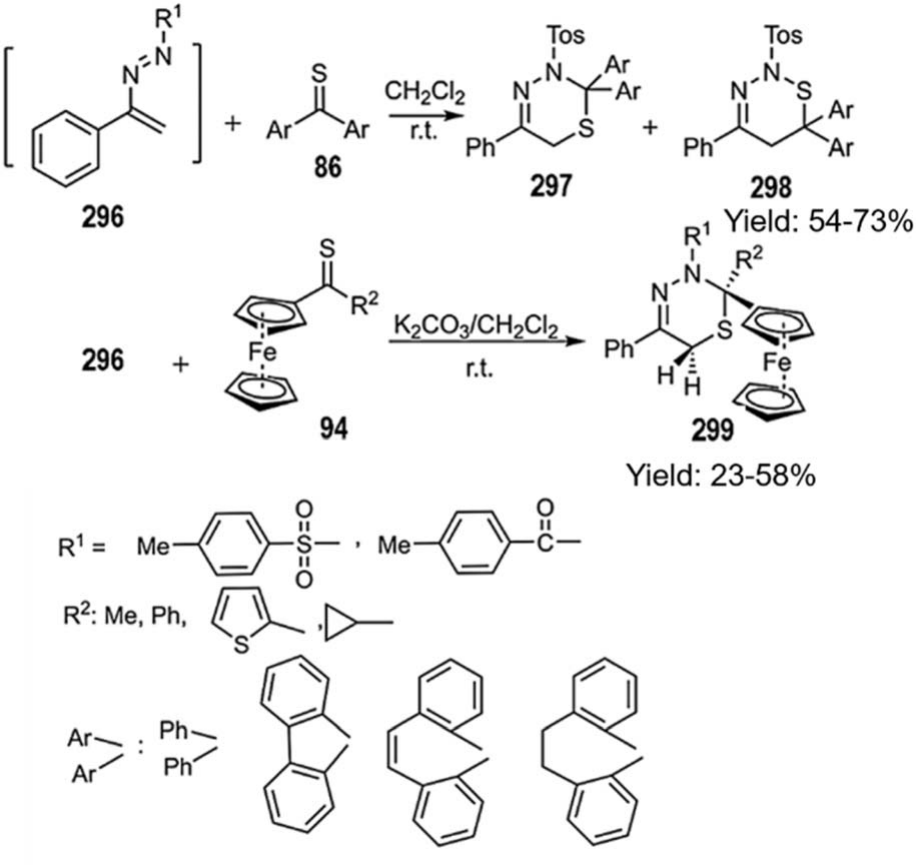
Thio D–A reaction of -phenylvinyldiazene structures 296 with thioketones.

A highly efficient method for the synthesis of valuable 1,5,2-oxathin derivatives has been developed through the thio D–A reaction of phenylvinyldiazene structures 296 thioketones. DFT revealed that, within this protocol, thioketone derivatives with steric hindrance perform the reaction in two steps by proceeding through a zwitterionic intermediate. In contrast, derivatives free from steric pressure perform a single-step D–A reaction. Key advantages of this approach include mild reaction conditions, excellent yields, and the regioselective synthesis of a Hexagon ring incorporating three heteroatoms (Scheme 85).^193^

#### 1.2.2 Diradical intermediate

In contrast, diradicals are reactive intermediates that feature two unpaired electrons and typically exist in a high-energy state.^194^ These intermediates are characteristic of stepwise mechanisms. The generation of diradicals may occur through thermal activation, photoinduced processes, or specific catalytic pathways, often involving homolytic bond cleavage in precursor molecules.^171,195–202^ Difficalt rotation around double bonds can hinder access to stable products, contributing to the formation of diradical intermediates.^67,166^ The TS for such mechanisms often involves the cleavage of three-center bonds. If the concert energy is large, the reaction proceeds along the coordinated path. While, if the reactions with lower energy, it will be accompanied by the formation of biradical intermediates.^194,197^ Once formed, diradicals may recombine with another reactant or themselves to yield the final cycloadduct. The stability of diradical intermediates is pivotal for the success of stepwise cycloadditions, with factors such as steric effects, electronic properties, and resonance playing a stabilizing or destabilizing role. For instance, in [4 + 2] cycloadditions, a diradical intermediate may form when a diene interacts with an electron-deficient dienophile *via* a stepwise pathway.^203,204^ The initial interaction generates the diradical intermediate, which subsequently rearranges into the final cyclic product. Bulky substituents can impede concerted mechanisms, favouring stepwise pathways that involve diradicals. Its notable that the literature has reported limited cases on the formation of diradical intermediates in [4 + 2] cycloadditions.^65,205^

**Scheme 86:**
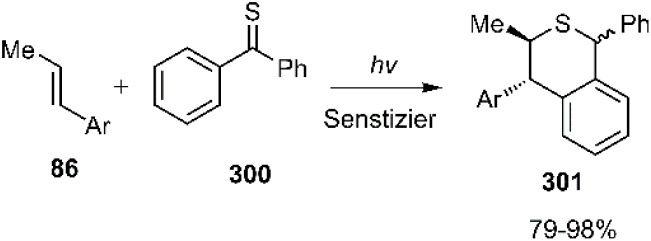
D–A reaction of thiobenzophenone 86 with the aryl alkenes 300.

Miranda's research group elucidated the thio D–A reaction of thiobenzophenone 86 with diverse aryl alkenes 300, encompassing both EWGs and EDGs. This reaction proceeds through the intermediate with a radical nature, as depicted in Scheme 86. Subsequent, comprehensive DFT studies have predicted a stepwise mechanism initiated by an ion-molecule complex.^206^

**Scheme 87:**
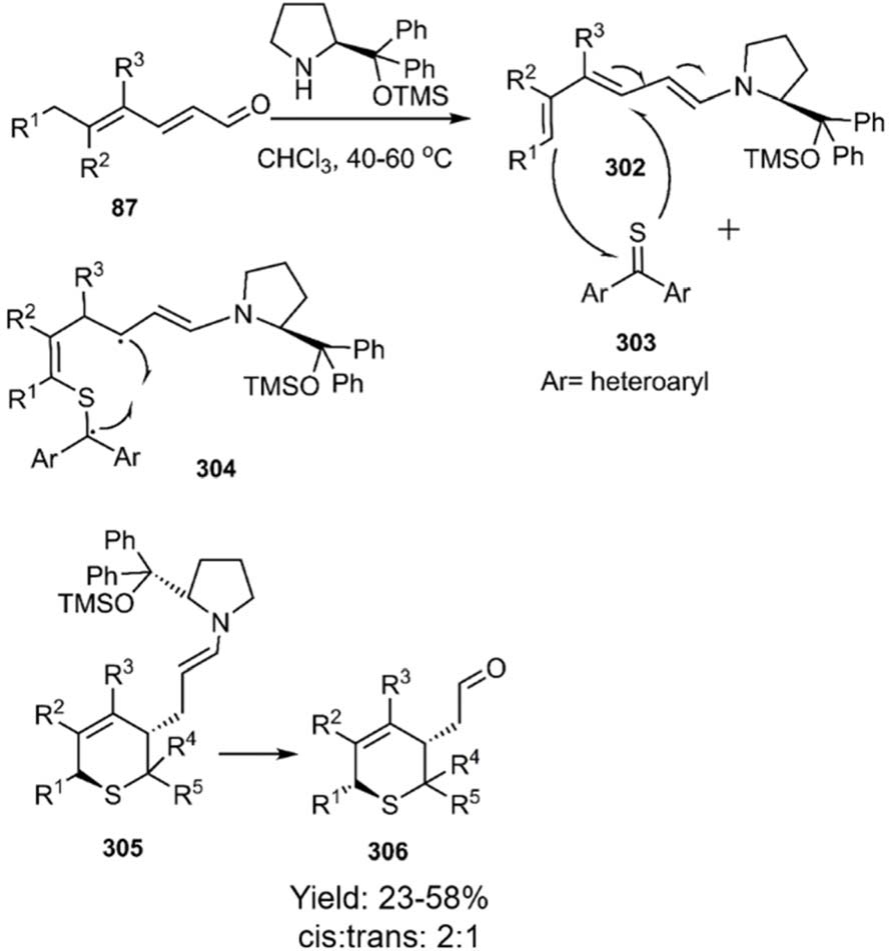
Mechanism of enantioselective synthesis of thiopyrans 306.

An elegant approach for the enantioselective synthesis of thiopyran derivatives 349*via* a step-wise mechanism was reported by Mlostoń *et al.* A detailed mechanism was shown to two diradicals intermediate in equilibrium. Chiral organocatalysts based on pyrrolidine promote the cycloaddition of related thioketones 303 with diene 87, affording a diverse array of thiopyrans incorporating heteroaryl moieties (Scheme 87).^207^

**Scheme 88:**
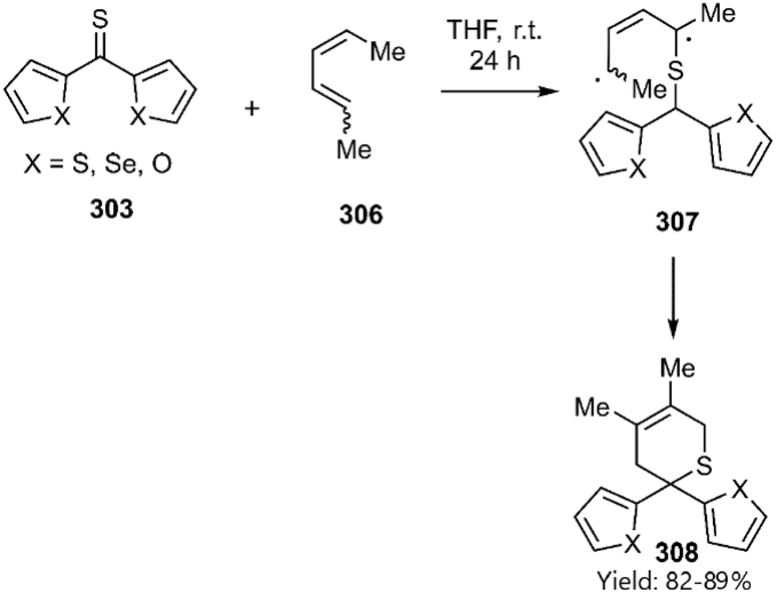
Step-wise D–A reaction of hetero aryl thioketones 303.

According to a report released by Mlostoń *et al.*, the hetero-D–A reaction of heteroaryl thioketones 303 with dimethyl butadiene isomers likely proceeds through a stepwise diradical mechanism. Heteroaryl thioketones exhibit exceptional dienophile character, readily reacting with inactivated dienes. The observed restriction of rotation around the C–C bond leading to the exclusive formation of the *cis*-thiopyran isomer 308 at ambient temperature, strongly supports this mechanism and facilitates the synthesis of more sustainable products (Scheme 88).^103^

**Scheme 89:**
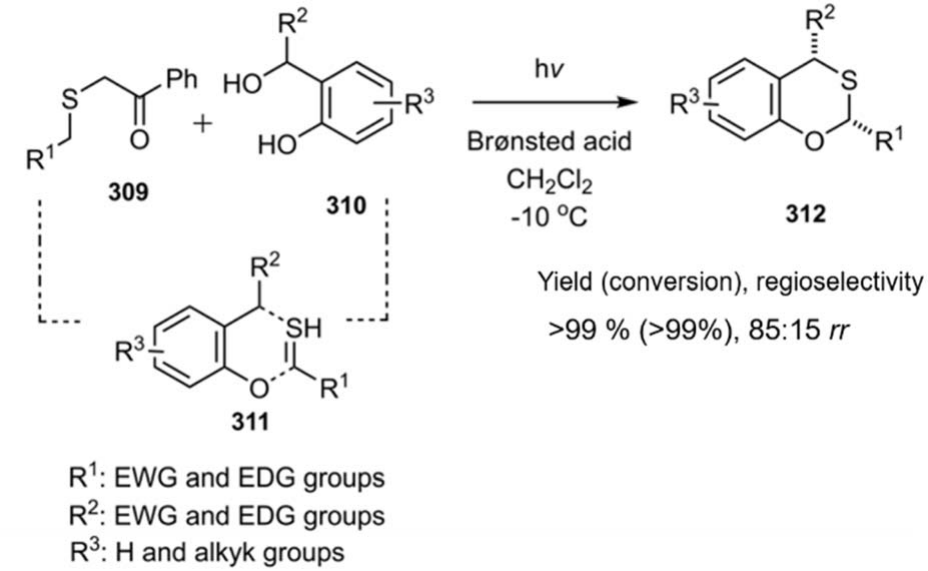
Preparation of 3,6-dihydro-2*H*-thiopyrans 312*via* thio D–A reaction.

Schneider and colleagues employed an innovative approach for the *in situ* generation of the desired thiodienophile from its active precursor 309. This strategy involved the photochemical synthesis of a thioaldehyde, which was subsequently trapped intramolecularly *via* a thio D–A reaction, enabling the efficient and large-scale preparation of 3,6-dihydro-2*H*-thiopyrans 312 (Scheme 89).^207^ Owning to expand their idea, this group evaluated reactions compounds 309 exposed various electron-deficient dienes. Ultraviolet irradiation and the presence of a diphenyl phosphate acid catalyst accelerated the reaction, leading to the synthesis of diverse product derivatives in high yields. Although for the authors, the step-by-step reaction was not proven. But according to the reaction conditions and relying on similar works in the literature, the existence of diradical intermediates is more probable.^208^

## 2 Intramolecular cycloaddition reaction

Intramolecular [4 + 2] cycloaddition reactions are characterized by their rapid reaction kinetics, enhanced frontier orbital overlap, and favourable stereoselectivity, owing to the inherent proximity of the D–A partners within the same molecule. This process affords the concerted formation of two rings in a single step. In addition to the six-membered ring arising from the [4 + 2] cycloaddition, the product incorporates a second ring whose size is dictated by the tether length connecting the diene and dienophile moieties. The intramolecular D–A reaction is typically irreversible and proceeds efficiently in the absence of significant steric hindrance. However, in certain instances, bond formation and cleavage are not strictly concerted, and the intermediary of radical species has been validated. This reaction constitutes a pivotal step in numerous total syntheses and the preparation of complex heterocyclic frameworks, frequently featuring as a key component in domino Knoevenagel thio D–A sequences.^188,189,208–215^

**Scheme 90:**
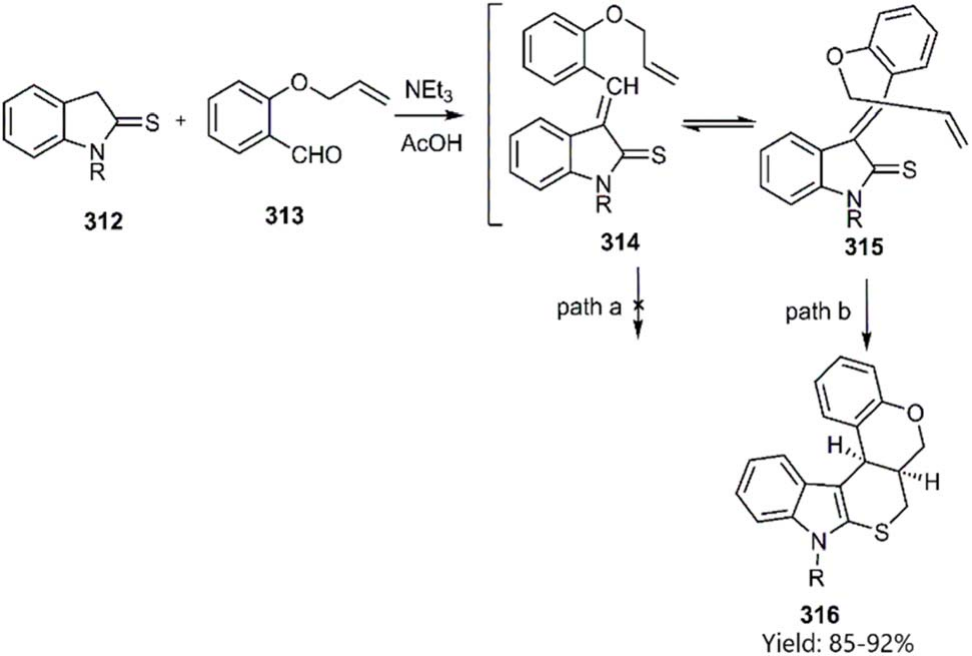
Syntheses of the polycycles 316.

A novel pentacyclic scaffold was synthesized *via* a domino Knoevenagel-thio D–A employing indoline-2-thione 312 and *o*-alkylated aromatic aldehydes 313. The formation of the *endo*-cycloadduct as the major product 316 is attributed to a favoured TS (path b). In contrast, the *exo*-addition was hindered by the sp^2^-geminal effect and 1,3-allylic strain, as reported by Majumdar and co-worker (Scheme 90).^216,217^

**Scheme 91:**
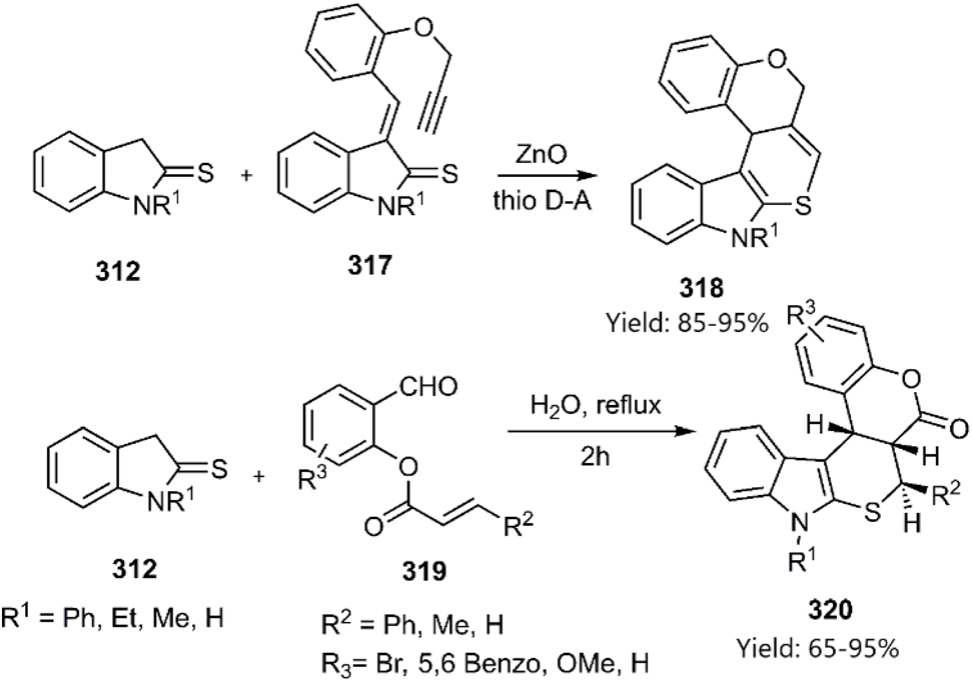
Synthesis of thiopyran-based heterocycles 318 and 320*via* knoevenagel thio D–A reaction.

Moghaddam's research group has extensively investigated the synthesis of thiopyran derivatives through KTDA reactions. They harnessed the reactivity of unsaturated indoline-2-thione to access this motif. Treatment of indoline-2-thione 312 with *o*-propargylated salicylaldehydes 317 and *o*-acrylated salicylaldehydes 319 generated the Knoevenagel adducts Subsequent intramolecular cycloaddition of this intermediates afforded the pentacyclic products 318 and 320 in satisfactory yields (Scheme 91).^21,218^

**Scheme 92:**
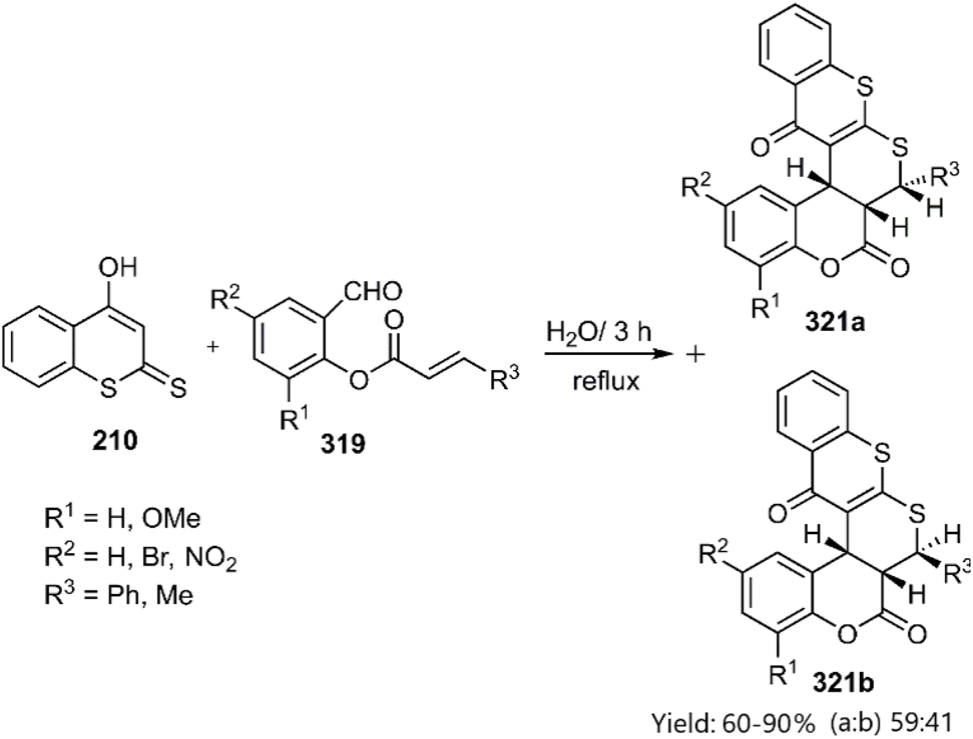
Synthesis of hybrid analogs of thiopyrans 321*via* intramolecular KTD reaction.

Moghaddam and coworkers explained a green and convenient Knoevenagel thio D–A reaction to achieve pentacyclic products. In the current process, the 4-hydroxydithiocoumarin 210, while subjected to a reaction with *o*-acrylated salicylaldehyde 319 gave a mixture of diastereomers 321a and 321b (Scheme 92).^219^

**Scheme 93:**
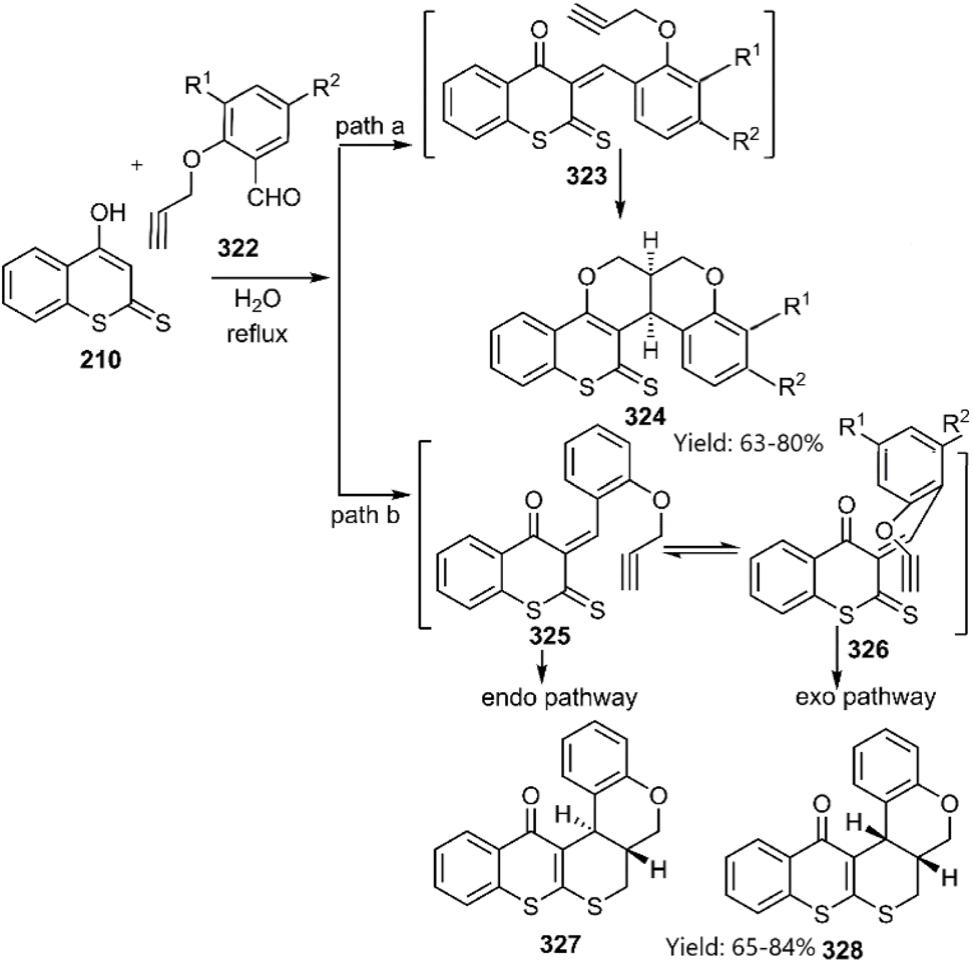
Application of 4-hydroxy-2*H*-thiochromene-2-thion 210 in KTD reaction.

Majumdar's group reported the synthesis of benzopyran-fused thiopyrano[2,3-*b*]thiochromen-5(4*H*)-ones from 4-hydroxy-2*H*-thiochromene-2-thione 210*via* an intramolecular thio D–A reaction (Scheme 93).^220,221^ This reaction proceeds through a catalyst-free *endo*-selective cycloaddition of a condensate intermediate in an aqueous medium. The authors attributed the *endo*-selectivity to the sp^2^-geminal effect and 1,3-allylic strain, favouring the *endo*-orientation (path b).

**Scheme 94:**
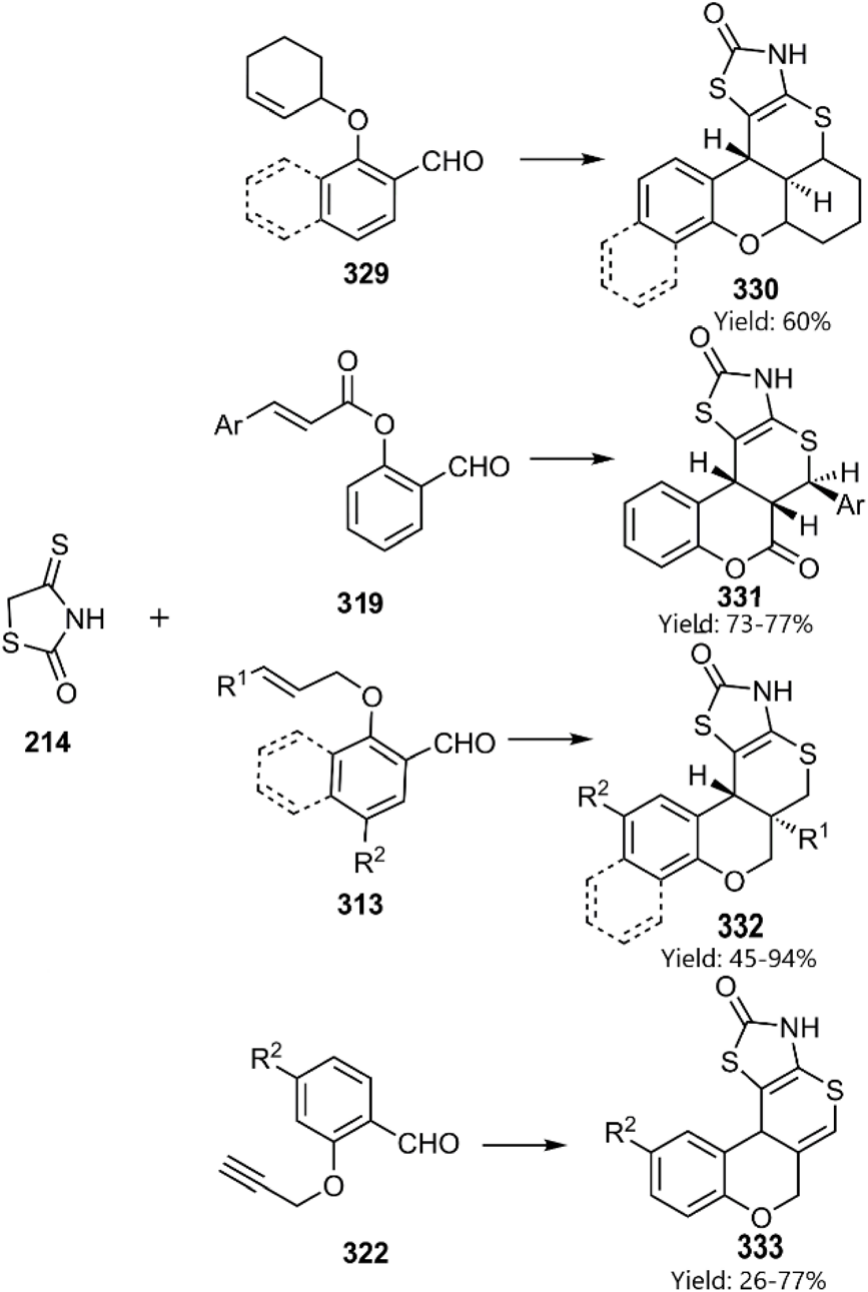
KTDA reaction of thioxothiazolidin-2-one 214.

Research Obushak *et al.*, conducted a series of continuous studies exploring KTDA reaction thiazolidin-2-ones. Based on this, they reported the synthesis of biologic polycyclic systems incorporating a thiopyran moiety. Their approach involved the condensation of 4-thioxo-1,3-thiazolidin-2-one 214 with functionalized aldehydes, followed by an intramolecular cycloaddition *via* a half-chair TS. This strategy preferentially yielded the stereoselective products (Scheme 94).^222–224^ However, during successive research activities, Lesyk and colleagues focused on the Investigating the therapeutic properties of heterocycles with thiopyrano[2,3-*d*]thiazole scaffolds. They proved that N–H proton facilitated the functionalization of this position, which was found to influence the anticancer activity of the resulting fused thiopyrano[2,3-*d*]thiazole compounds.^160,223^

**Scheme 95:**
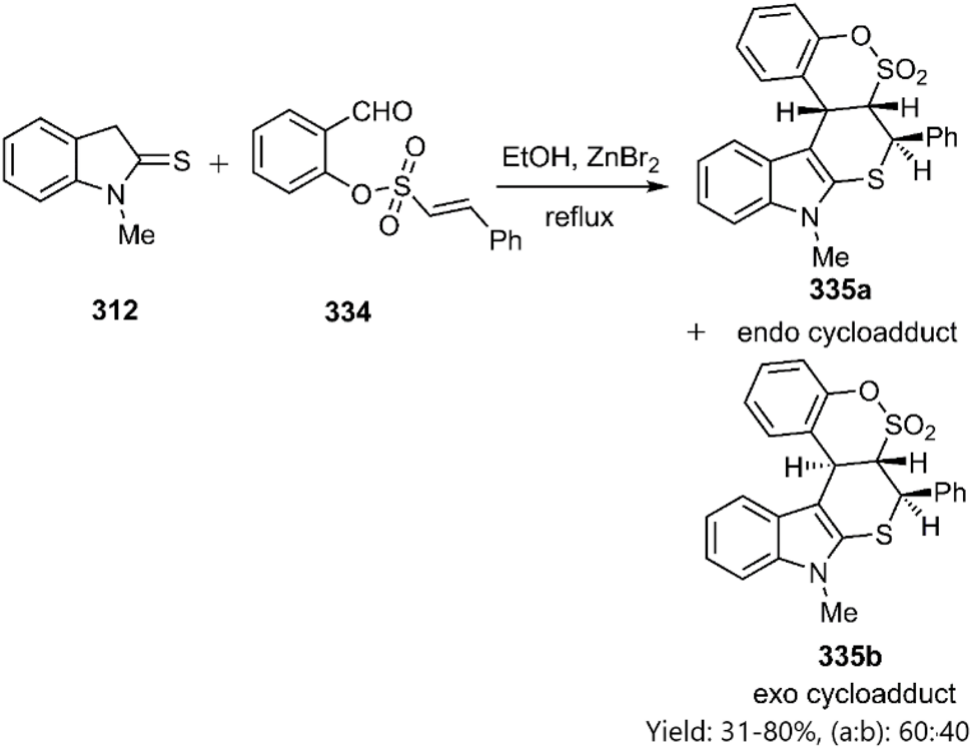
KTDA reaction of indoline-2-thione 312.

Moghaddam *et al.* employed 2-formylphenyl (*E*)-2-phenylethenyl sulfonates 334 to the synthesize novel thiopyranoindole-fused benzo-*d*-sultone derivatives 335. The observed diastereoselectivity indicated a preference for the *endo*-approach (Scheme 95)^225^

**Scheme 96:**
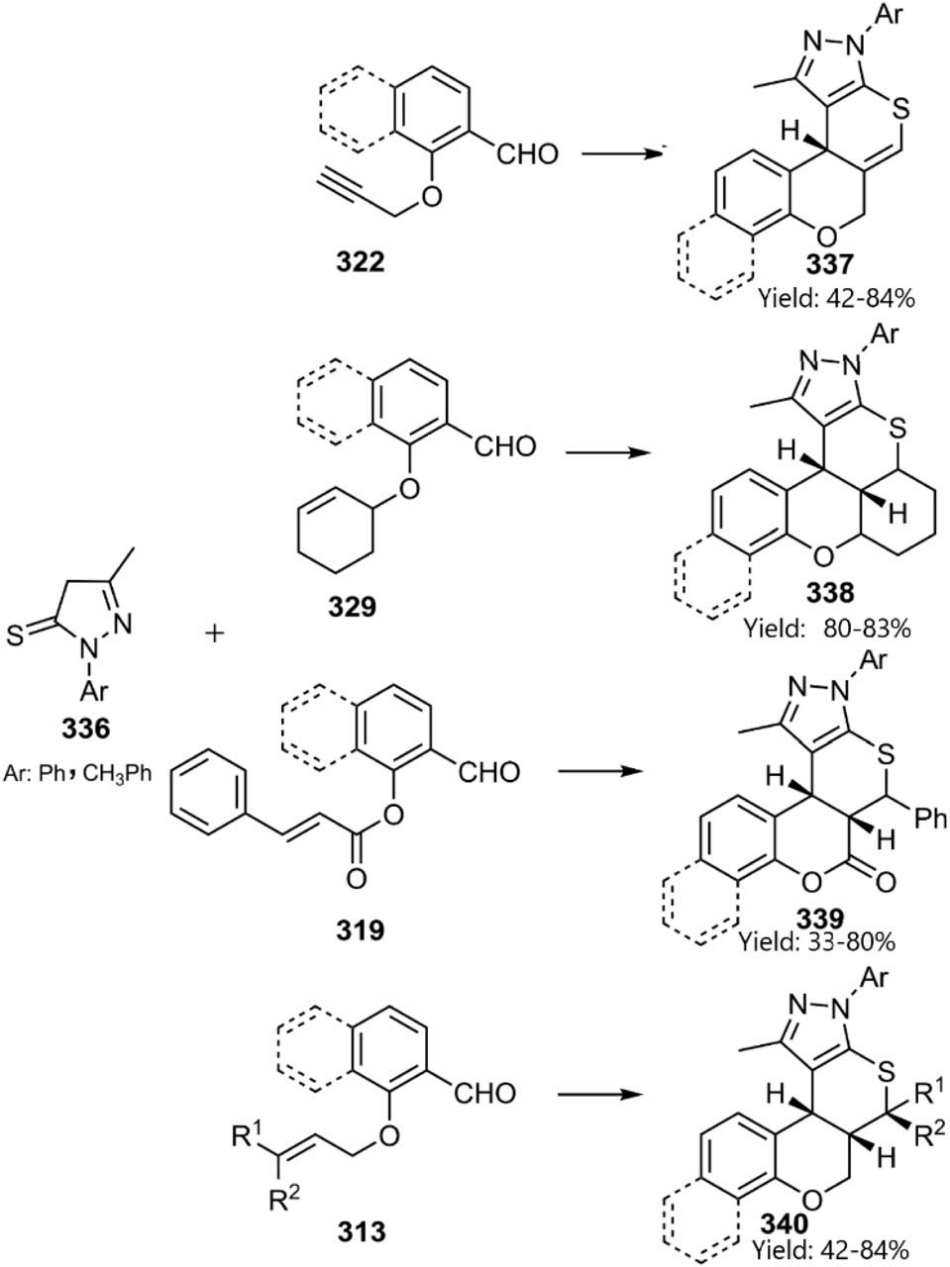
Intramolecular thio D–A reaction of unsaturated pyrazol-5-thiones.

Parmar and colleagues developed KTDA reactions involving pyrazol-5-thiones 324 and functionalized salicylaldehydes in an ionic liquid. They noted that excellent diastereoselectivity is governed by the stereobarrier imposed on the rotational TS (Scheme 96).^226,227^

**Scheme 97:**
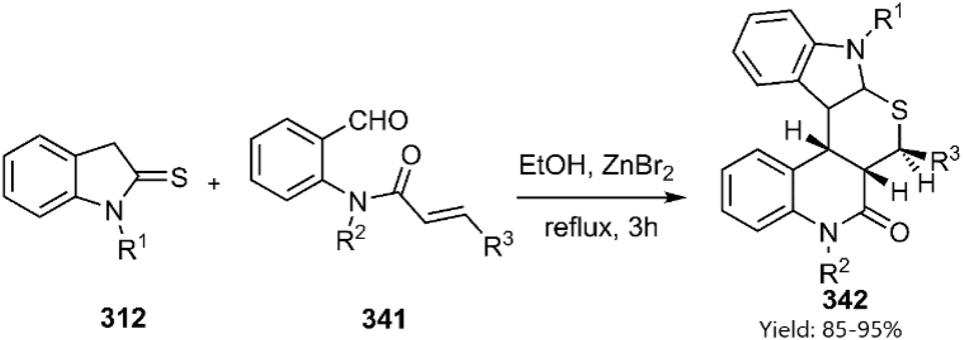
KTDA reaction of indoline-2-thione 312.

Kiamehr *et al.* have designed an efficient one-pot stereo and regioselective domino KTDA reaction. In this manner, synthesis of pentacyclic thiopyranoindol fused [3, 4-*c*] quinolone structures 399 has been accomplished from indoline-2-thions 365 and provided *N*-acrylated anthranilaldehyde derivatives 398 under direction of ZnBr_2_ in refluxing ethanol (Scheme 97).^228^

**Scheme 98:**
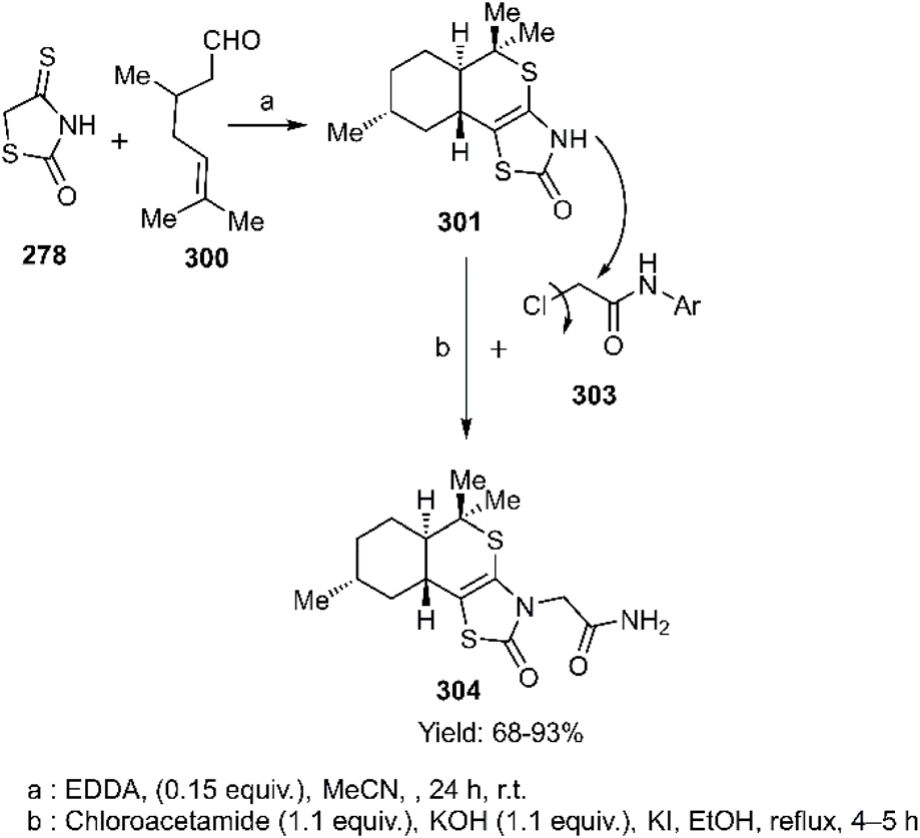
Synthesis of amide-substituted isothiochromenothiazole derivatives 346.

Lesyk and colleagues developed a concise synthesis of amide-substituted isothiochromenothiazole derivatives 346 through base-promoted alkylation of the D–A cycloadduct led to the reconnaissance of an anticancer agent featuring the functionalized isothiochromenothiazole moiety (Scheme 98).^56,229^

**Scheme 99:**
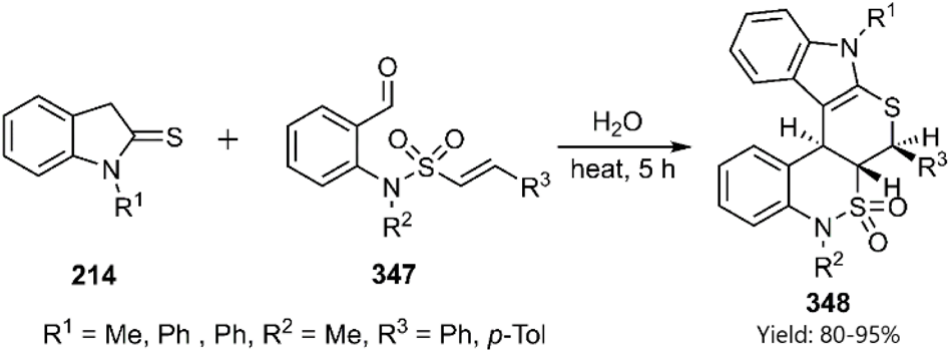
Synthesis of heterocycles containing benzosultam-annulated thiopyranoindole 348.

Expanding upon pervious their work, Kiamehr research group employed formylphenylsulfonamides 347 to synthesize novel benzosultam-annulated thiopyranoindole derivatives 348. These products are conveniently generated through a KTDA reaction (Scheme 99).^230^

## 3 Conclusion

The widespread use of sulfur-containing heterocyclic drugs, ranging from penicillin to olanzapine, has inspired organic synthesis chemists to explore novel structures of sulfur-based pharmaceuticals. The therapeutic potential of sulfur heterocycles underscores the need for innovative strategies to optimize the production of these compounds. Among these, thiopyran derivatives have demonstrated significant medicinal properties, making their synthesis an appealing endeavour. The development of new strategies for synthesizing thiopyran derivatives represents a promising frontier in organic chemistry. One of the most effective methods for transforming organosulfur compounds into thiopyran derivatives is through cycloaddition reactions. These reactions provide a controllable and accessible means of forming heterocycles, with [4 + 2] cycloaddition standing out as a particularly attractive route for organochemists seeking to synthesize six-membered thiocycles. In this review, we present a comprehensive overview of the synthesis of thiopyran compounds, categorizing and analyzing the subject from multiple perspectives. Our approach encompasses both intramolecular and intermolecular [4 + 2] cycloaddition reactions, and we further classify these processes into concerted and stepwise mechanisms based on their reaction pathways. It seems that intramolecular Diels–Alder reactions often proceed by a concerted mechanism. While the Diels–Alder reaction is traditionally known for its concerted nature, recent literature has introduced the concept of stepwise D–A reactions. Understanding reaction pathway allows chemists to design new synthetic pathways for complex molecules, including natural products and pharmaceuticals. Concerted mechanisms have high stereospecificity. Depending on the nucleophilic and electrophilic characteristics of the D–A partners, reaction condition and the steric hindrance present in the precursors, the reaction pathway may deviate from the conventional D–A mechanism. Any factor that increases concerted energy leads the reaction to a stepwise mechanism. Our objective is to elucidate the formation of thiopyran derivatives through both concerted and stepwise pathways, facilitating an indirect comparison between these two approaches in the literature. The stepwise D–A mechanism may involve zwitterionic intermediates or diradicals, contingent upon the polarity and steric effects within the TS. Notably, the mechanisms governing these reactions necessitate more extensive computational analyses than those required for concerted processes. It seems that the burgeoning interest in stepwise cycloaddition reactions has captivated researchers in the field. Scientists are actively seeking to manipulate these reaction processes through various substitutions, adjustments to reaction polarity, and the application of different catalysts— a pursuit that remains ripe for exploration and realization.

## Abbreviations

HOMO: Highest occupied molecular orbital
LUMO: Least unoccupied molecular orbital
D–A: Diels–Alder
HD–A: Hetero-Diels–Alder
HDDA: Hexadehydro-Diels–Alder
ELF: Electron localization function
DFT: Density functional theory
TS: Transient transition state
RAFT: Radically mediated chain transfer
EWG: Electron-withdrawing group
EDG: Electron-donating group
MCRs: Multi component reactions
COSY: COrrelation spectroscopY
HSQC: Heteronuclear single quantum coherence
HMBC: Heteronuclear multiple bond coherence
NOESY: Nuclear overhauser spectroscopy
KDO: Deoxy manno octulosonic acid
*o*-TQs: *o*-Thio quinones
KTD–A: Knoevenagel thio Diels–Alder reaction

## Data availability

All data analyzed in this review are publicly available from various sources, including published articles and books. The specific studies and datasets referenced in this review can be found in the citations provided throughout the manuscript. No new data were generated during this review, apart from the conclusion and analysis of the collected data set.

## Conflicts of interest

There are no conflicts to declare.
